# Single-cell gene-expression measurements in *Vibrio cholerae* biofilms reveal spatiotemporal patterns underlying development

**DOI:** 10.1101/2024.07.17.603784

**Published:** 2024-07-17

**Authors:** Grace E. Johnson, Chenyi Fei, Ned S. Wingreen, Bonnie L. Bassler

**Affiliations:** 1. Department of Molecular Biology, Princeton University, Princeton, NJ 08544, USA.; 2. The Howard Hughes Medical Institute, Chevy Chase, MD 20815, USA.; 3. Lewis-Sigler Institute for Integrative Genomics, Princeton University, Princeton, NJ 08544, USA.; 4. Princeton Center for Theoretical Science, Princeton University, Princeton, NJ 08544, USA.; 5. Lead Contact

## Abstract

Bacteria commonly exist in multicellular, surface-attached communities called biofilms. Biofilms are central to ecology, medicine, and industry. The *Vibrio cholerae* pathogen forms biofilms from single founder cells that, via cell division, mature into three-dimensional structures with distinct, yet reproducible, regional architectures. To define mechanisms underlying biofilm developmental transitions, we establish a single-molecule fluorescence in situ hybridization (smFISH) approach that enables accurate quantitation of spatiotemporal gene-expression patterns in biofilms at individual-cell resolution. smFISH analyses of *V. cholerae* biofilm regulatory and structural genes demonstrate that, as biofilms mature, matrix gene expression decreases, and simultaneously, a pattern emerges in which matrix gene expression is largely confined to peripheral biofilm cells. Both quorum sensing and c-di-GMP-signaling are required to generate the proper temporal pattern of matrix gene expression, while c-di-GMP-signaling sets the regional expression pattern without input from quorum sensing. The smFISH strategy provides insight into mechanisms conferring particular fates to individual biofilm cells.

## Introduction

Bacteria commonly exist in spatially-organized, surface-attached communities called biofilms. Biofilms provide advantages to the cells residing within them, including protection from environmental insults such as antimicrobials, predators, and mechanical perturbation.^1–4^ Critical to the biofilm lifestyle is the production of an extracellular matrix that attaches cells to the surface and to one another.^5^ Biofilm cells can degrade the matrix, exit the community, and return to the individual, planktonic lifestyle in a process called dispersal.^6,7^ The ability to transition between biofilm and planktonic lifestyles allows bacteria to respond to changing environments and, moreover, is required for many pathogens to successfully cause disease.^6^ For example, *Vibrio cholerae*, the etiological agent of the disease cholera and the model organism used in this work, forms biofilms both in its marine niche and in human hosts, and successive rounds of biofilm formation and dispersal are key to transmission of cholera disease.^8,9^

**Fig. 1.**
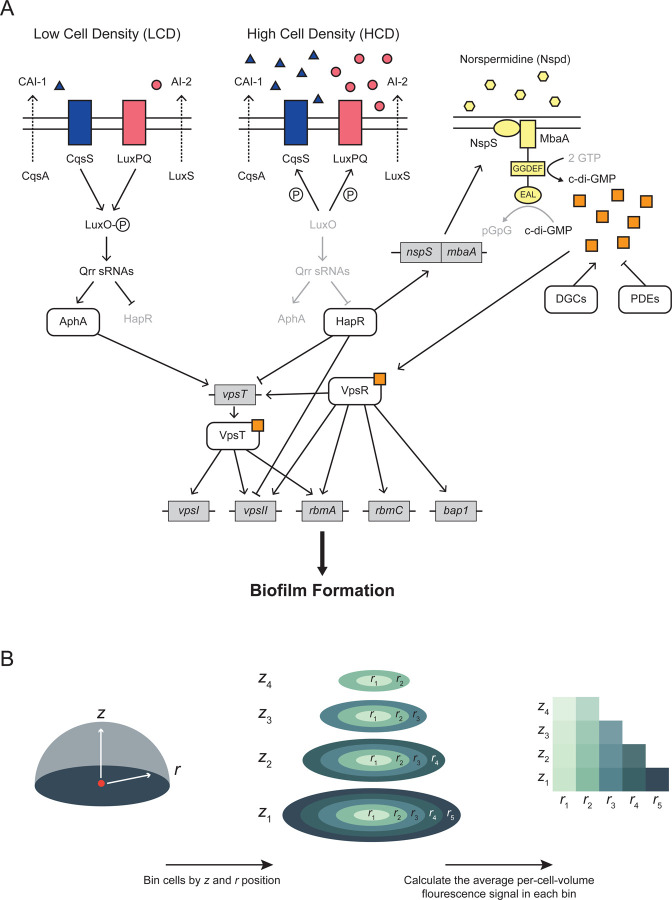
Regulation of biofilm formation in *V. cholerae*. (A) Simplified model of regulation of biofilm formation by quorum sensing (QS), c-di-GMP signaling, and norspermidine (Nspd). See text for full details. Blue triangles represent CAI-1; pink circles represent AI-2; yellow hexagons represent Nspd; orange squares represent c-di-GMP. The P in a circle represents phosphate. The GGDEF and EAL designations represent the motifs on MbaA that confer the DGC and PDE activities, respectively. (B) Schematic overview of quantitation of spatial gene expression patterns in biofilms. Briefly, cells are grouped by their *r* and *z* positions, and the average per-cell-volume fluorescence signal in each group is calculated. For full details, see Methods: Analysis of spatial gene-expression patterns at single-cell resolution.

In *V. cholerae*, the major component of the matrix is an exopolysaccharide called *Vibrio* polysaccharide (VPS).^10^ The enzymes necessary for VPS synthesis are encoded in two operons, *vpsI* and *vpsII*. In addition to VPS, the *V. cholerae* extracellular matrix contains three major proteins: RbmA, which promotes cell-cell adhesion, Bap1, important for cell-surface attachment, and RbmC, which, along with Bap1 and VPS, forms envelopes that encapsulate cell clusters.^11–13^ Expression of the two *vps* operons, *rbmA*, *bap1*, and *rbmC*, and correspondingly, the production of matrix, is regulated by two sensory inputs, the small second messenger molecule cyclic diguanylate (c-di-GMP) and quorum-sensing-mediated chemical communication (QS).^14–16^

c-di-GMP controls *V. cholerae* matrix production by binding to and activating two transcription factors, VpsR and VpsT, which in turn, drive transcription of matrix genes (Fig. 1A).^15^ c-di-GMP is synthesized and degraded by enzymes with diguanylate cyclase (DGC) and phosphodiesterase (PDE) activities, respectively.^17^ *V. cholerae* DGC and PDE activities are controlled by environmental stimuli, including, and germane to the present work, the polyamine norspermidine (Nspd). Nspd, via its NspS receptor, drives increases in c-di-GMP production by interacting with the bifunctional enzyme MbaA, activating its DGC activity and suppressing its PDE activity (Fig. 1A).^18^

QS is the process of bacterial communication in which cells produce, release, and detect extracellular signal molecules called autoinducers to regulate group behaviors, including biofilm formation (Fig. 1A).^19,20^ *V. cholerae* possesses multiple QS autoinducer-receptor pairs, two of which are relevant to the present work. The first autoinducer is *cholerae* autoinducer-1 (CAI-1; (*S*)-3-hydroxytridecan-4-one), which is synthesized by CqsA and detected by the CqsS receptor.^21,22^ The second autoinducer is autoinducer-2 (AI-2; (2S,4*S*)-2-methyl-2,3,3,4-tetrahydroxytetrahydrofuran borate), which is synthesized by LuxS and detected by the LuxPQ receptor.^23,24^ At low cell densities (LCD), when autoinducer concentrations are low, the unbound CqsS and LuxPQ receptors act as kinases and shuttle phosphate to the LuxO response regulator.^25,26^ LuxO~P activates expression of genes encoding a set of small RNAs (sRNAs) called the Qrr sRNAs, which activate production of the master LCD regulator AphA and repress production of the master high-cell-density (HCD) regulator HapR.^27,28^ Conversely, at HCD, when CAI-1 and AI-2 have accumulated, the autoinducer-bound receptors act as phosphatases, LuxO is dephosphorylated, and transcription of the *qrr* genes terminates.^25–27^ As a result, AphA production ceases, and HapR production commences.^28^ HapR represses transcription of *vpsT* and the *vpsII* operon.^29,30^ Thus, at LCD, when HapR levels are low, matrix production, and consequently, biofilm formation, occur, while at HCD, when HapR levels are high, matrix production is repressed, promoting biofilm disassembly and cell dispersal (Fig. 1A).^31^

Analogous to eukaryotic embryos, bacterial biofilms often arise from single cells that develop into multicellular structures via cell division.^32^ Recent advances in microscopy have made it possible to image and track individual live bacteria over the initial phase of biofilm development, revealing that cells reproducibly adopt unique cell fates depending on their locations in the emerging biofilm.^32–34^ We know that cells are organized into distinct clusters surrounded by non-uniform distributions of matrix components.^12^ Furthermore, individual-cell characteristics, such as orientation, packing with other cells, and trajectories from birth to final destination have been quantified and exhibit location-dependent differences.^33,34^ Nonetheless, a mechanistic understanding of how individual cell fates arise is still lacking because accurately measuring individual-cell gene-expression patterns in space and time in developing biofilms has remained a hurdle.

Here, we describe the development and application of a biofilm-specific single-molecule fluorescence in situ hybridization (smFISH) approach that overcomes many of the challenges that have previously limited individual cell gene-expression measurements in biofilms. We first deliver the biofilm-specific smFISH strategy and validate that it accurately quantifies individual-cell gene expression in biofilms. Second, we use the smFISH approach to quantify the spatiotemporal expression patterns of biofilm regulators and downstream structural components. We find that QS is required to establish the overall temporal expression pattern of key biofilm matrix genes, while c-di-GMP signaling confines expression of those matrix genes to distinct biofilm sub-regions. Together, this work provides the first measurements of gene expression in single cells in *V. cholerae* biofilms and insight into the molecular mechanisms that confer particular fates to individual cells in biofilms.

## Results

### Developing smFISH for accurate quantitation of single-cell gene expression in bacterial biofilms

Understanding how individual bacteria residing in biofilm communities adopt location-specific cell fates requires measurements of spatiotemporal gene expression at single-cell resolution. Traditional fluorescent reporters are inadequate for this task because their output is dampened by the low oxygen levels that exist in biofilms, which prohibits proper reporter maturation.^35^ Indeed, in *V. cholerae* biofilms, signal from multiple fluorophores rapidly declines over time, well before biofilms reach maturity. Reductions in fluorescence output occur on a timescale comparable to the cell doubling time (Fig. S1A), making fluorescent reporters poor proxies of gene expression in *V. cholerae* biofilms. One solution to this problem employed recently is to grow biofilms under flow, and thus supply a constant source of oxygen.^36,37^ While this strategy eliminates fluorescent signal decay, it prevents the natural accumulation of small signal molecules, for example QS autoinducers, that are required to properly regulate the biofilm lifecycle.

Compounding issues with traditional fluorescent reporters, biases inherent to confocal imaging preclude accurate quantitation of spatial fluorescence patterns in biofilms.^34^ *z*-positional bias exists because captured fluorescence signal declines as a function of distance from the objective. Radial (*r*)-positional bias is likely caused by the biofilm’s dome shape. Specifically, as the center of the biofilm is thicker and contains more layers of cells than does the edge, background fluorescence from out-of-focus *z* planes is higher at the biofilm center than at the periphery. Indeed, thinner biofilms with fewer *z* planes display less dramatic *r*-positional bias. Consistent with these imaging biases, we show that signal from DAPI stained biofilms, which is expected to be spatially uniform, decreases from the bottom to the top and from the inside to the outside of *V. cholerae* biofilms (Fig. S1B).

With the aim of overcoming the above limitations and developing a robust tool for measuring single-cell gene expression in bacterial biofilms, we adapted smFISH technology. Our logic is as follows: first, smFISH has been successfully used in planktonic bacteria to accurately measure single-cell gene expression at ranges from <1 to 100 mRNA molecules per cell.^38,39^ Second, smFISH does not require an oxygen-dependent maturation step, making it suitable for use in bacterial biofilms. Third, smFISH does not rely on the generation of fusion proteins that can be impaired in their biological functions and can, therefore, fail to accurately report on the underlying biology.^40^ Indeed, sequential smFISH (seqFISH) was recently used to measure the expression of over 100 genes in individual *Pseudomonas aeruginosa* biofilm cells.^41^ The analyses focused on coarse differences in gene expression that occurred at length scales of tens of microns. This first gene-expression map of a bacterial biofilm community revealed neighborhoods of cells with distinct metabolic activities and demonstrated the potential for smFISH to be applied to biofilm cells. However, further refinement of smFISH was required to accurately capture gene-expression changes occurring at the single micron (i.e., one cell) distances necessary to probe *V. cholerae* biofilms, which, at their maximum size prior to initiation of dispersal, are roughly 20–30 µm in radius. Below, we combine smFISH with confocal imaging and a biofilm-specific downstream analysis pipeline to accurately quantify individual-cell gene expression over space and time in *V. cholerae* biofilms. Our approach provides a powerful tool for such analyses, including for transcripts that display only low-level expression and for proteins that are recalcitrant to tagging. Both of these issues have persisted as obstacles that until now have stymied single-cell bacterial gene-expression measurements, including in planktonic samples grown under well-oxygenated conditions.

Our first goal was to assess the imaging artifacts associated with our smFISH strategy independent of any biological gene-expression patterns. To do this, we measured the smFISH fluorescence signal from a gene expressed uniformly across the biofilm. We grew *V. cholerae* biofilms containing arabinose-inducible *mNG* integrated into the chromosome. Previous reports demonstrated uniform cell growth across the biofilm under our conditions^34^, and thus, *mNG* expression levels should track with the exogenously supplied arabinose inducer concentration. To ensure equivalent arabinose penetration throughout the biofilm, it was added at the start of the experiment, prior to biofilm formation. Importantly, while *V. cholerae* cells are able to import arabinose, they are not known to metabolize it, and thus arabinose concentrations should remain spatially uniform and constant over the course of growth.^42^ We grew biofilms in the presence of high and low concentrations of arabinose and measured *mNG* transcripts with smFISH probes labeled with one of the three fluorescent dyes used throughout the remainder of this work. The fluorescence signal from the mNG protein does not survive the fixation and smFISH hybridization process, and thus does not add to nor interfere with quantitation of smFISH fluorescence signal. We quantified smFISH fluorescence signal at roughly single-cell resolution (2.32 µm) by (1) dividing biofilms into cubes with 2.32 µm sides, (2) identifying and assigning cell volume and smFISH fluorescent puncta to these cubes, and (3) calculating the total smFISH fluorescence signal associated with puncta in each cube relative to the fraction of the cube occupied by cell volume (see Methods). We refer to these resulting values henceforth as ‘per-cell-volume fluorescence signal’. This strategy allowed us to ascertain the fluorescence signal biases across entire biofilms for each fluorescent probe, and over a range of transcript levels. *mNG* expression levels assessed by each fluorophore in biofilms were similar to those in planktonic cells grown with the corresponding inducer concentration. (Fig. S2A).

**Fig. 2.**
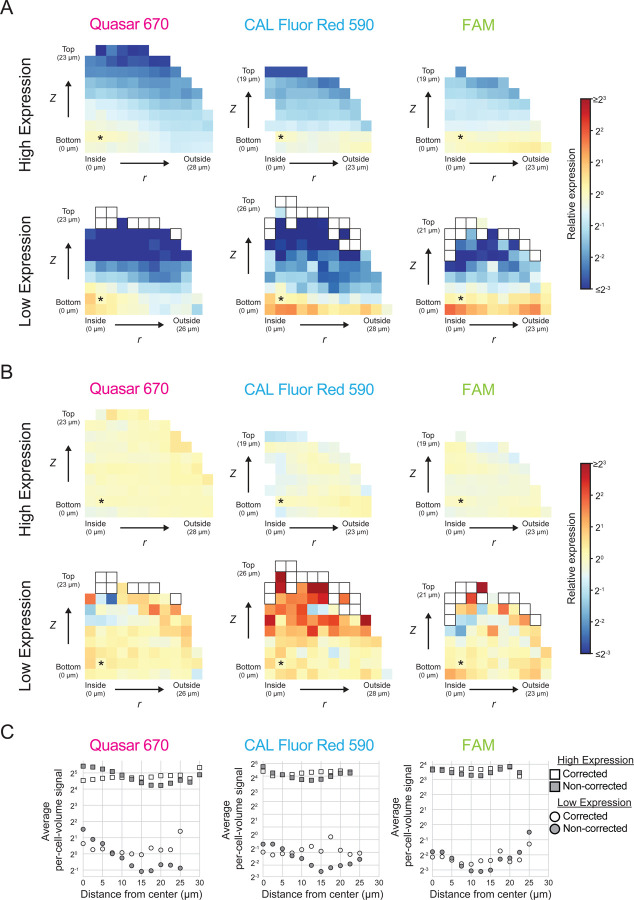
Quantitation of single-cell gene expression in *V. cholerae* biofilms. (A) Heatmaps showing relative, non-corrected spatial *mNG* per-cell-volume fluorescence signal data as a function of position *r, z* in biofilms of *V. cholerae* carrying pBad-*mNG*. Biofilms were grown in the presence of 0.2% or 0.0375% arabinose, denoted High and Low Expression, respectively. 100 µM Nspd was included in all cases. *mNG* expression was measured by smFISH using probes labeled with one of three fluorophores, as indicated. Cells are grouped into bins with size Δ*r,* Δ*z* = 2.32, 2.32 µm. Gene expression in each bin is represented as a relative value, where the average per-cell-volume fluorescence signal across all cells in a bin is normalized to the average per-cell-volume fluorescence signal across all cells in the bin denoted with the asterisk (bin *r, z* = 2.32, 2.32 µm). White boxes represent zero values. (B) Heatmaps as in (A) showing the relative, corrected per-cell-volume fluorescence signal values. (C) Average per-cell-volume fluorescence signal versus distance to the biofilm center (*r, z* = 0, 3.5 µm) is shown for each fluorophore as indicated. The corrected and non-corrected data are shown for biofilms with high and low *mNG* expression. All data represent *n* = 10–12 biofilms. See also Figs. S1, S2.

To quantify spatial smFISH fluorescence signal patterns, we grouped cells across replicate biofilms by their *r* and *z* positions. We calculated the average per-cell-volume fluorescence signal for each group of cells relative to the average per-cell-volume fluorescence signal of a group of cells located near the center of the biofilm (Fig. 1B, Methods). The *r* and *z* positions of each group, and the corresponding average per-cell-volume fluorescence signals relative to that in the reference group of cells with position *r, z* = 2.32, 2.32 µm, can be visualized as a heatmap, as in Fig. 2A. We also calculated the distance of each cell from the center of the biofilm (Methods), and binned cells by this value to determine the average per-cell-volume fluorescence signal as a function of distance from the biofilm center (Fig. 2C). Of note, per-cell-volume fluorescence signal is reported here and throughout this work in arbitrary units (AUs) that are roughly equivalent to the number of transcripts per cell-volume. Transcript numbers can be calculated as described previously by identifying the fluorescence distributions of individual puncta reporting on transcripts that are produced at low levels, where the signal from most puncta is predicted to arise from an individual mRNA molecule.^38^ In our experiments, the individual mRNA puncta intensities cluster around 1, differing by no more than 2-fold across all fluorophores and all genes under study (Fig. S2B). Thus, a cell with fluorescence signal of 1 AU contains approximately one mRNA transcript for the gene whose expression is being quantified.

Absent fluorescence signal biases, and given uniform expression of *mNG*, one would expect unvarying fluorescence signal across all cells in the biofilm. This is not the case. Rather, spatially-dependent fluorescence signal differences are observed, in which average per-cell-volume fluorescence signal is highest in cells closest to the center of the biofilm, and lowest in cells most distant from the center (Fig. 2A, C). The pattern can be described by both *z* and *r* positional biases consistent with the known confocal imaging issues described above. Crucially, the fluorescence signal pattern is the opposite of what would be predicted if the arabinose inducer and/or *mNG* probes could not penetrate the biofilms. In both of those cases, one would expect lower per-cell-volume fluorescence signal in the center of the biofilm and higher per-cell-volume fluorescence signal at the biofilm periphery. This result gives us confidence that the spatial fluorescence signal pattern associated with constitutive gene expression arises entirely from the expected confocal imaging artifacts.

To account for these artifacts, we developed a mathematical model to quantify the above fluorescence signal positional biases for each fluorophore and across different gene-expression levels. When we use this method to correct the *mNG* expression data, we observe uniform average *mNG* per-cell-volume fluorescence signal throughout biofilms, consistent with our expectation for a constitutively expressed gene (Fig. 2B, C). The average per-cell-volume fluorescence signal appears less uniform in biofilms that display low *mNG* expression as a consequence of higher expression variation between cells. The average per-cell-volume fluorescence signal is most noisy in bins far from the surface, the region with the least fluorescence signal. However, 97% of bins across biofilms with low *mNG* expression have a *z*-score between −0.25 and 0.25, i.e., deviate from the average per-cell-volume fluorescence signal of the center bin by no more than one quarter of a standard deviation, demonstrating that there are minimal fluorescence signal output differences between groups of cells. The model is not only able to correct spatial fluorescence signal biases in the large biofilms used to generate the model, but can also be directly applied without modification to accurately correct biases from smaller biofilms grown with identical concentrations of arabinose inducer (Fig. S2C, D). Full details of how we developed the model and how it is applied are provided in Methods. All main text figures show data that has been corrected using our model. All companion plots generated from the non-corrected data are provided as supporting data, aside from select examples that are included in supplementary figures as noted. Descriptions of per-cell-volume fluorescence signal and expression levels henceforth are also based on corrected data, unless otherwise noted. Using our model to correct for spatial fluorescence signal imaging biases overcomes a major limitation of confocal imaging and provides us with a *V. cholerae* biofilm-specific analysis pipeline that delivers accurate spatial and temporal gene-expression patterns at single-cell resolution.

### smFISH accurately measures QS gene expression in biofilms

We validated that our smFISH technique could accurately quantify single-cell gene expression in *V. cholerae* biofilms across space and time. Here, we focus on genes that control the *V. cholerae* biofilm lifecycle, i.e., the genes encoding the QS LCD and HCD master regulators, *qrr*4 and *hapR*, respectively, and the downstream QS-controlled target gene *vpsL*, the first gene in the *vpsII* operon that encodes VPS biosynthetic enzymes. HapR represses *vpsL* at HCD (Fig. 1A). Regarding the strategy of using the *qrr*4 gene as the readout for the QS LCD state, the *aphA* gene encoding the LCD master transcription factor would have been the obvious choice (Fig. 1A). However, AphA is a small protein encoded by a short transcript, and despite multiple attempts, we were unable to design smFISH probes against the *aphA* transcript that yielded measurable fluorescence signal. Attempts to construct AphA fusions to increase transcript length destabilized the mRNAs and they could not be detected by smFISH nor qRT-PCR. The sRNA Qrr4 is also, by definition, a small transcript. Thus, to monitor the QS LCD state, we used smFISH probes against *mNG* encoded under the p*qrr*4 promoter that has previously been demonstrated to accurately report *qrr*4 levels.^43,44^

To validate our approach, we compared the fluorescence signal detected by smFISH in biofilms to that from an established technology, smFISH probing planktonic cultures. We know that QS gene expression in *V. cholerae* biofilms can be dramatically different from that in planktonic cells. Thus, for this series of experiments, we exploited a *V. cholerae* strain in which we could control its QS status. Specifically, the *V. cholerae* strain contains only a single QS receptor, LuxPQ, which detects the autoinducer AI-2. The strain also contains a deletion of the gene encoding the AI-2 synthase, *luxS*. Therefore, the level of expression of QS-regulated genes is set exclusively by exogenously supplied AI-2, the amount of which we can precisely control. From here forward, we call this strain ‘the AI-2 sensor strain’. We grew planktonic and biofilm cultures of the AI-2 sensor strain without AI-2 or with a saturating AI-2 concentration (10 µM).^45^ This approach enables comparison of gene expression between biofilm and planktonic cells because it forces the strain into the LCD and HCD states, respectively, and in turn drives maximal/minimal *qrr*4 and minimal/maximal *hapR* expression. Moreover, because QS gene expression at each AI-2 concentration is fixed and unvarying in the AI-2 sensor strain, we could assess the accuracy of our measurements over time and space in biofilms. Specifically, we confirmed temporal accuracy by measuring expression in biofilms grown without or with AI-2 over a range of biofilm developmental stages, and we confirmed spatial accuracy by measuring the pattern of gene expression across entire biofilms. Important for our strategy, and as mentioned in the Introduction, is that *V. cholerae* forms biofilms at LCD and disperses from biofilms at HCD. Thus, in all cases, the AI-2 sensor strain was grown in the presence of the polyamine norspermidine (denoted Nspd) that prevents biofilm dispersal at HCD.^46^

**Fig. 3.**
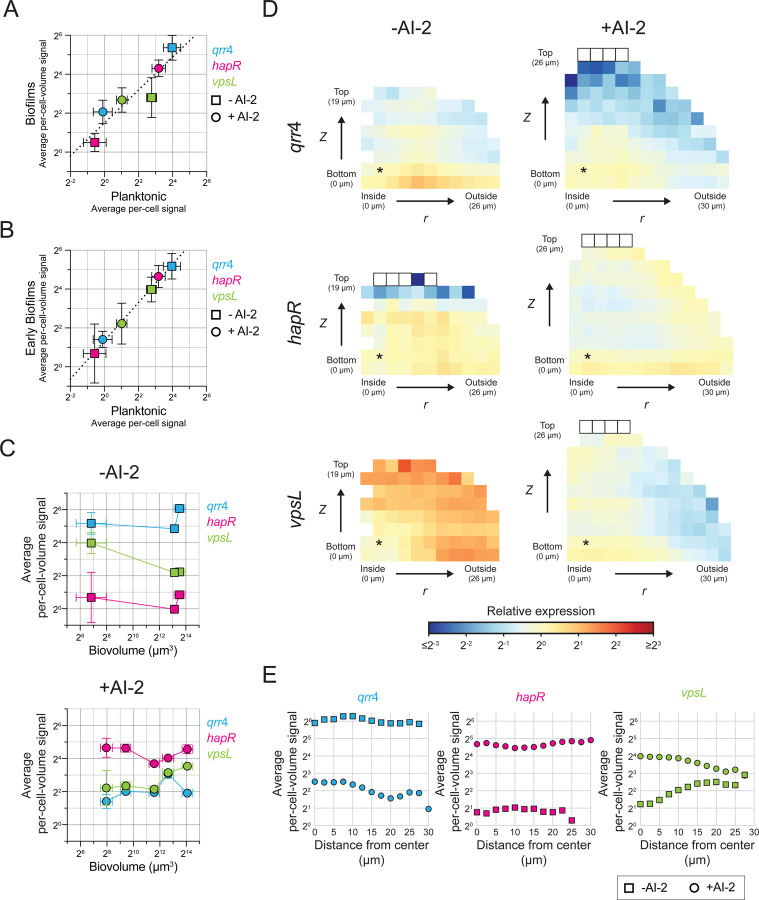
smFISH accurately quantifies QS gene expression in single cells of *V. cholerae* in biofilms. (A) Average per-cell-volume *qrr*4 (*mNG*), *hapR,* and *vpsL* fluorescence signal in *V. cholerae* planktonic and biofilm cells. Values for planktonic samples represent the means of *n* = 3 biological replicates. Values for biofilm samples represent the means across all biofilm sizes, as shown in (C). The slope of the best fit line as shown is 0.80. The slope of the best fit line excluding *vpsL* is 0.88. (B) As in (A) with values for biofilm samples calculated as the means across *n* = 9–10 replicate biofilms with the smallest biovolumes shown in (C). The slope of the best fit line is 0.99. (C) Average per-cell-volume *qrr*4 (*mNG*), *hapR*, and *vpsL* fluorescence signal across replicate *V. cholerae* biofilms grown without (top) or with (bottom) 10 µM AI-2 as a function of the average biofilm biovolume. (D) Heatmaps of relative *qrr*4 (*mNG*), *hapR*, and *vpsL* average per-cell-volume fluorescence signal relative to the average per-cell-volume signal in the bin denoted with an asterisk are shown as in Fig. 2B for *V. cholerae* biofilms grown without (left) and with 10 µM AI-2 (right), for *n =* 10 replicate biofilms with the largest biovolumes shown in (C). (E) Average per-cell-volume fluorescence signal versus distance to the biofilm center, as in Fig. 2C, for the data shown in (D). Error bars denote standard deviations, which are in some cases smaller than the sizes of the symbols used in the plots. Best fit lines were calculated by fitting log_2_ transformed data. All biofilms were grown with 100 µM Nspd. All data have been corrected using the model described in the text (See Methods: Spatial correction model). See also Figs. S3, S4.

First, regarding the QS regulators *qrr*4 and *hapR*, there is good agreement in the AI-2 sensor strain between expression measured in biofilms and planktonic cells by smFISH (Fig. 3A, B). Specifically, high *qrr*4 expression and low *hapR* expression occur in the -AI-2 (LCD) samples, and low *qrr*4 and high *hapR* expression occur in +AI-2 (HCD) samples (Figs. 3A-C, S3A). Importantly, the average per-cell-volume expression of *qrr*4 and *hapR* measured both relative to each other and across the LCD and HCD conditions are consistent between planktonic and biofilm cells (Fig. 3A, slope of best fit line on a log-log scale for *qrr*4 and *hapR* points is 0.88), confirming the accuracy of our smFISH strategy for measuring gene expression in biofilms. Moreover, for any given condition and gene studied, the global average per-cell-volume fluorescence signal differed by no more than 2-fold over time and across a range of biofilm sizes (Fig. 3C). Following application of our spatial correction model to these data (Fig. S3B, C), we find appropriate high or low expression of each gene under each condition, and, crucially, consistent expression across the biofilm (Figs. 3D, E, S4A, B). These data highlight the power of our model to correct for imaging biases irrespective of the specific transcript being measured, and they demonstrate how the correction can be used to obtain accurate single-cell-level spatial gene-expression patterns in biofilms for, in principle, any gene of interest.

The data for expression of *vpsL* in the AI-2 sensor strain differs markedly from that of the two upstream QS regulators. *vpsL* exhibits changes in both its temporal and spatial expression patterns. We come back to the molecular mechanism underlying these results below, but briefly, the patterns occur because *vpsL* is regulated by both QS and by the small molecule second messenger cyclic diguanylate (c-di-GMP)^29^, levels of which vary in space and time in biofilms of the AI-2 sensor strain. A few key points pertinent to the validation of our approach are relevant. First, to confirm the accuracy of measurements of *vpsL* expression, we compared *vpsL* expression levels in planktonic cells to those in the smallest biofilms in our analyses. In nascent biofilms, cells have only recently attached to the surface and begun the transition from the planktonic to the biofilm lifestyle. Thus, the biological state of *V. cholerae* cells in immature biofilms should closely resemble that of planktonic cells. Indeed, there is good agreement between *vpsL* expression levels in planktonic and early-stage biofilm cells under both LCD and HCD conditions (Fig. 3B). Additionally, there is high correlation between expression levels for all three measured genes in planktonic and early-stage biofilm cells (Fig. 3B, slope of best fit line on a log-log scale is 0.99). Thus, our approach also allows comparisons of expression levels across genes probed with different fluorophores. Second, unlike *qrr*4 and *hapR*, at LCD (-AI-2) *vpsL* is expressed more highly at the biofilm periphery than in the biofilm core (Fig. 3D, E). This pattern cannot be an artifact of our spatial correction, as the pattern is present in the non-corrected data (Fig. S3B, C). Again, we explain the biology underlying formation of this pattern below. Taken together, our measurements of *hapR*, *qrr*4, and *vpsL* expression demonstrate that our smFISH strategy for probing single-cell gene expression in *V. cholerae* biofilms can accurately quantify gene expression, both temporally and spatially.

### *V. cholerae* cells transition from the LCD to the HCD QS state as biofilms mature

**Fig. 4.**
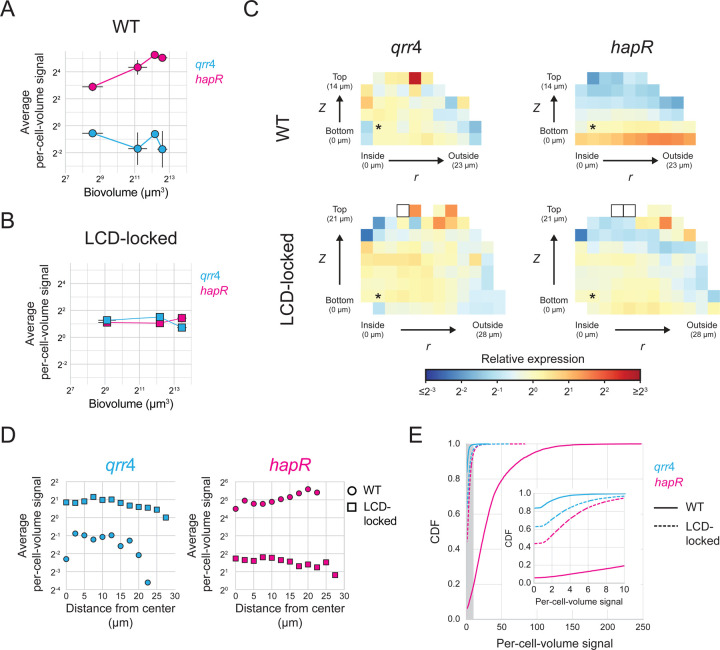
QS signaling over *V. cholerae* biofilm development. (A) Average per-cell-volume *qrr*4 (*mNG*) and *hapR* fluorescence signal across *n* = 10 replicate WT *V. cholerae* biofilms as a function of the average biofilm biovolume. (B) Average per-cell-volume *qrr*4 (*mNG*) and *hapR* fluorescence signal across *n* = 9–10 replicate *V. cholerae* biofilms formed by the LCD-locked *luxO D61E* strain as a function of the average biofilm biovolume. (C) Heatmaps of *qrr*4 (*mNG*) and *hapR* average per-cell-volume fluorescence signal relative to the average per-cell-volume fluorescence signal in the bins denoted with asterisks are shown as in Fig. 2B for *n* = 9–10 replicate WT (top) and LCD-locked (bottom) *V. cholerae* biofilms with the largest biovolumes shown in (A) and (B), respectively. (D) Average per-cell-volume fluorescence signal versus distance to the biofilm center, as in Fig. 2C, for the data shown in (C). (E) Cumulative distributions of per-cell-volume *qrr*4 (*mNG*) and *hapR* fluorescence signals across individual cells in WT biofilms and biofilms of the LCD-locked strain shown in (C). The inlay shows the region of the distribution highlighted in gray. Error bars denote standard deviations, which are in some cases smaller than the sizes of the symbols used in the plots. All data have been corrected using the model described in the text (See Methods: Spatial correction model). See also Fig. S5.

Equipped with our smFISH strategy, we measured the spatiotemporal gene-expression patterns of key biofilm regulators and structural components in wildtype (WT) *V. cholerae* that undergoes the full biofilm lifecycle, from attachment to dispersal. We focused first on QS signaling and used *qrr*4 and *hapR* as readouts of the LCD and HCD QS states of cells, respectively. To capture different biofilm developmental stages, we grew biofilms for different lengths of time, ranging from six to ten hours, before fixing the samples and conducting smFISH. Consistent with previous data^31^, as biofilms mature and biovolumes increase, expression of *qrr*4 decreases and expression of *hapR* increases (Fig. 4A), reflecting the transition from the QS LCD- to the HCD-state. Indeed, in the largest biofilms assayed, *hapR* levels are comparable to those measured in biofilms of the AI-2 sensor strain grown with saturating AI-2 (Fig. 3A-C, +AI-2). We interpret these results to mean that over the course of WT *V. cholerae* biofilm development, autoinducer concentrations accumulate to the level required to fully engage the QS system.

To verify the above results, we performed control analyses in which we measured *qrr*4 and *hapR* levels over time in biofilms of *V. cholerae* carrying the constitutively active LuxO~P phosphomimetic allele, *luxO D61E*. LuxO D61E locks cells into the LCD QS state.^25^ We refer to such samples as ‘LCD-locked’. As expected, given that the LCD-locked strain cannot undergo the QS transition to HCD, expression of *qrr*4 and *hapR* remained consistent across biofilm development (Fig. 4B). Furthermore, *qrr*4 expression was higher and *hapR* expression was lower in biofilms formed by the LCD-locked strain than in WT biofilms. These results confirm that the *qrr*4 and *hapR* expression changes that occur in WT *V. cholerae* biofilms are a consequence of QS. We note that *hapR* levels in the biofilms formed by the LCD-locked strain are identical to those measured in biofilms of the AI-2 sensor strain grown in the absence of AI-2 (i.e. when the AI-2 sensor strain is in the LCD QS state), however, *qrr*4 levels are lower in biofilms formed by the LCD-locked strain than in biofilms formed by AI-2 sensor strain under the no AI-2 condition (Fig. 3A-C, -AI-2). We do not know the mechanism underlying this discrepancy. There exist multiple feedback loops in the QS pathway.^20,43,44^ We speculate that feedback onto *qrr* expression occurring in one of these two mutant strains but not in the other could be responsible for the difference.

### QS autoinducers are spread uniformly across mature *V. cholerae* biofilms

A question that has long perplexed the biofilm field is whether QS autoinducers are present at uniform concentrations across a biofilm, or alternatively, if there exist regions of high and low autoinducer concentrations that could drive regional differences in gene-expression patterns. It is not yet possible to directly measure autoinducer concentrations in biofilms. However, the spatial accuracy that our smFISH method delivers in quantitation of gene expression in biofilms allows us to probe this longstanding question, at least in the case of *V. cholerae* biofilms. Specifically, by comparing expression levels of QS-controlled genes throughout biofilms, we can infer whether autoinducers are uniformly present or vary regionally. As expected, in biofilms containing the LCD-locked strain, there is uniform spatial expression of both *qrr*4 and *hapR* across the biofilm (Fig. 4C, D). Similar constant, location-independent expression patterns of *qrr*4 and *hapR* occur in WT biofilms suggesting that autoinducers can freely diffuse to achieve uniform and/or saturating concentrations across the community of cells (Fig. 4C, D).

Although *qrr*4 and *hapR* do not display regional expression heterogeneity in *V. cholerae* biofilms, there is nonetheless location-independent expression variability across individual cells (Fig. 4E). To investigate the source underlying this heterogeneity, we measured the relationship between *qrr*4 and *hapR* expression across cells (see Methods). Our logic is that, if location-independent non-uniformities in autoinducer concentration exist across the biofilm, the consequence would be differences in the QS states of individual cells. In that case, we would expect *qrr*4 and *hapR* expression to be negatively correlated in WT biofilms, but not in biofilms of the LCD-locked strain that is impervious to autoinducers. We do not detect any correlation between *qrr*4 and *hapR* expression in either strain, a finding consistent with intrinsic noise driving all observed heterogeneity (Fig. S5A, B).^47^ We also measure negative correlations between expression variation (measured by the coefficient of variation, CV) and expression levels for both *qrr*4 and *hapR* across samples, another feature of intrinsic expression noise (Fig. S5C)^47^. This correlation is consistent across biofilms formed from the WT, LCD-locked, and AI-2 sensor strains, and between biofilm and planktonic samples, further suggesting that autoinducer fluctuations, which would be distributed and detected differently among these conditions, do not drive gene-expression heterogeneity.

### *vpsL* is preferentially expressed in cells located at the periphery of mature *V. cholerae* biofilms

Two important changes occur as *V. cholerae* cells transition from the planktonic lifestyle to the biofilm mode: (1) alteration in expression of hundreds of genes that enable biofilm formation and that adapt cells to the new lifestyle, and (2) organization of cells into a stereotypical yet non-uniform architecture. We hypothesized that discrete spatiotemporal patterns of expression of biofilm structural genes could arise, providing a link between gene-expression changes and specific biofilm architectural transitions. To explore this possibility, we focused first on *vpsL*, which, as mentioned, encodes a biosynthetic enzyme required for extracellular matrix polysaccharide production. *vpsL* is regulated by both QS and c-di-GMP signaling (Fig. 1A), allowing us to probe the contributions of both pathways to spatiotemporal gene-expression patterning. Quantitation of *vpsL* expression over time and space was assessed in the same biofilms in which spatiotemporal QS autoinducer input was measured (Fig. 4). We reasoned that measuring *vpsL* patterns in WT *V. cholerae* biofilms would enable us to assess the combined effects of QS and c-di-GMP signaling while measurements in biofilms of the LCD-locked strain would enable quantitation of the contribution of c-di-GMP signaling in isolation.

**Fig. 5.**
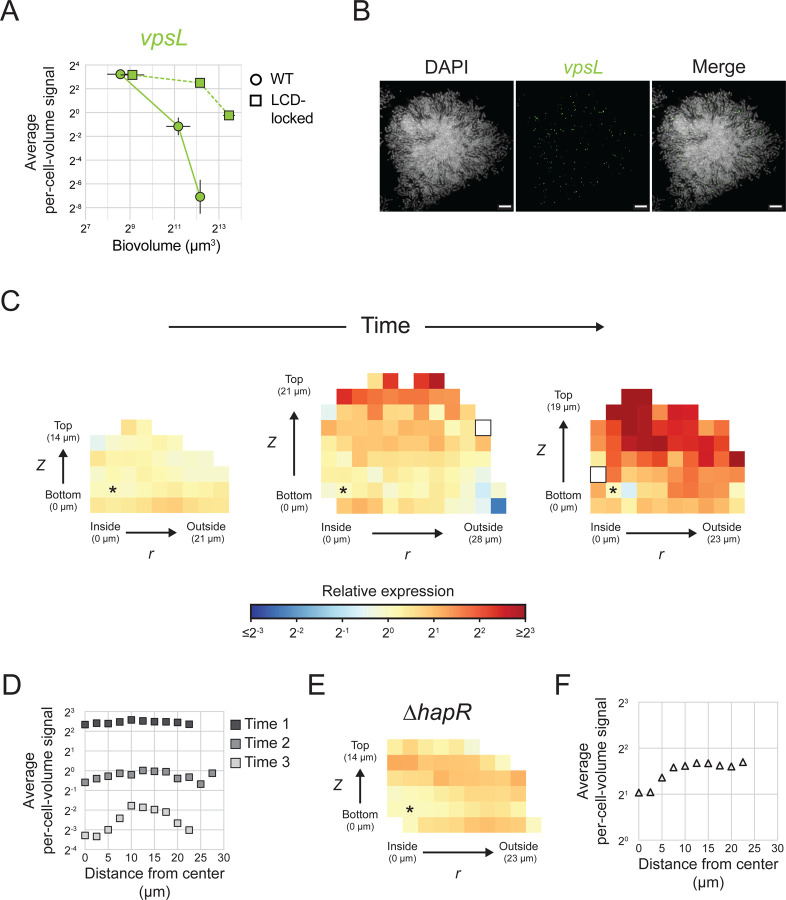
*vpsL* expression over *V. cholerae* biofilm development. (A) Average per-cell-volume *vpsL* fluorescence signal across *n* = 9–10 replicate WT biofilms or biofilms of the LCD-locked strain as a function of the average biofilm biovolume. None of the biofilms shown here have initiated dispersal (therefore, the biofilms in the rightmost panel of (C) are excluded). Error bars represent standard deviations, which are in some cases are smaller than the sizes of the symbols used in the plots. (B) Confocal microscopy images of DAPI and *vpsL* smFISH fluorescence signal in a representative *V. cholerae* biofilm formed by the LCD-locked strain at the timepoint represented by the middle panel in (C). Images are maximum projections of the first four in-focus *z* slices. Scale bar represents 5 µm. (C) Heatmaps of *vpsL* average per-cell-volume fluorescence signal relative to the average per-cell-volume fluorescence signal in the bins denoted with asterisks are shown as in Fig. 2B for *n* = 9–10 replicate biofilms formed by the LCD-locked strain at different timepoints over the course of development. (D) Average per-cell-volume fluorescence signal versus distance to the biofilm center, as in Fig. 2C, for the data shown in (C). Times 1, 2, and 3 correspond to the left, center, and right panels in (C), respectively. (E) As in (C) for *n* = 10 replicate biofilms formed by the ∆*hapR* strain. The color scale bar is as shown in (C). (F) As in (D) for data shown in (E). All data have been corrected using the model described in the text (See Methods: Spatial correction model). See also Fig. S5.

Overall *vpsL* levels decrease during maturation of both WT biofilms and biofilms formed by the LCD-locked strain (Fig. 5A). We expected this temporal pattern of expression because VPS production must terminate to allow cells to disperse from mature biofilms. Expression of *vpsL* declines more dramatically in WT biofilms than in the biofilms of the LCD-locked strain (Fig. 5A). Again, this difference is expected because HapR is a repressor of *vpsL*, and *hapR* is more highly expressed in mature HCD WT *V. cholerae* biofilms than in mature biofilms of the LCD-locked strain (Fig. 4A, B). The decrease in *vpsL* expression in the LCD-locked strain is similar to what occurs in biofilms of the AI-2 sensor strain in the absence of AI-2 (Fig. 5A and 3C, respectively). These temporal reductions in *vpsL* expression over biofilm development cannot be a consequence of QS regulation since both the LCD-locked and AI-2 sensor strains are QS inert, but rather, reflect changes in c-di-GMP signaling.

Our analyses show that *vpsL* expression declines to fewer than 1 mRNA per 100 cells in mature WT *V. cholerae* biofilms (Fig. 5A). Consequently, while we were able to monitor overall changes in *vpsL* levels by averaging over many biofilms and thousands of cells, we are unable to quantify spatial differences in *vpsL* expression in WT *V. cholerae* biofilms because, in local biofilm cell subpopulations that can contain fewer than 100 cells, we often detect zero *vpsL* mRNA transcripts. Fortuitously, mature biofilms of the LCD-locked strain, because they express overall higher levels of *vpsL* per cell than do mature WT biofilm cells, made it possible for us to probe spatial *vpsL* expression. In early-stage biofilms, *vpsL* is uniformly expressed across biofilms (Fig. 5C, left-most panel, and Fig. 5D, Time 1). In mature biofilms, *vpsL* transcript levels are higher in cells at the biofilm periphery than in cells located at the biofilm core, with the pattern becoming more dramatic as biofilms continue to develop (Fig. 5B, Fig. 5C, center and right-most panels, Fig. 5D, Time 2 and 3). Curiously, the most mature biofilms (Fig. 5C, right-most panel) have smaller average biovolumes than biofilms grown for shorter times (Fig. 5C, middle panel). Possibly, cells have begun to disperse from the most mature biofilms, reducing their biovolumes. While beyond the scope of this work, if this is indeed the case, establishing the proper *vpsL* spatial expression pattern may be crucial for *V. cholerae* to launch its dispersal program.

Our findings regarding QS and regulation of *vpsL* spatiotemporal expression in biofilms are as follows: QS is the major driver of temporal changes in *vpsL* expression over *V. cholerae* biofilm development, and QS is responsible for terminating *vpsL* expression at biofilm maturity. While QS-independent *vpsL* expression reductions occur over time in biofilms composed of QS-deficient strains, they are modest compared to those that occur in WT biofilms. We attribute the QS-independent temporal *vpsL* expression changes to declining c-di-GMP levels that occur concurrently with biofilm development, as discussed below. Regarding spatial *vpsL* regulation: QS cannot establish regional differences in *vpsL* expression due to invariant autoinducer concentrations across biofilms (Fig. 4C, D). Nonetheless, *vpsL* develops a distinct spatial expression pattern with higher expression in peripheral biofilm cells than in interior cells. This pattern persists in three QS-inert strains, including the Δ*hapR* strain that lacks the QS regulator of *vpsL* (Fig. 5E, F), further confirming the lack of a role for QS in establishing this spatial pattern. Indeed, no correlation exists between *hapR* and *vpsL* expression levels in groups of individual cells in WT biofilms or biofilms of the LCD-locked strain (Fig. S5D). Thus, any spatial heterogeneity in *vpsL* expression that is present in biofilms must be established independently of QS/HapR activity. c-di-GMP is the only other known regulator of *vpsL*, so we conclude that regional differences in c-di-GMP signaling across the biofilm are responsible for setting up local patterns of *vpsL* gene expression in biofilms. We further validate this assertion below.

### Spatiotemporal expression patterns of genes encoding matrix structural proteins parallel those of *vpsL* and are controlled by c-di-GMP signaling in *V. cholerae* biofilms

To further investigate how spatiotemporal gene-expression patterns are established during *V. cholerae* biofilm formation, we assessed the expression patterns of additional biofilm regulators and structural genes. Specifically, having considered QS regulation above, we now primarily focus on the regulators VpsR and VpsT that link alterations in c-di-GMP levels to changes in gene expression.^15^ VpsR and VpsT are transcription factors that bind c-di-GMP, and are active in their c-di-GMP-bound states. Once liganded, they promote expression of their downstream target genes. Transcription of *vpsT* is activated by the VpsR-c-di-GMP complex and repressed by HapR at HCD. This regulatory arrangement links *vpsT* levels to both c-di-GMP and to QS (Fig. 1A).^29^

We measured expression of *vpsR* and *vpsT* and genes encoding the three matrix proteins RbmA, RbmC, and Bap1 that are downstream targets of VpsR and/or VpsT (Fig. 1A). Our smFISH technology limits us to probing three genes in a given biofilm, so we measured genes in pairs; genes assayed in the same biofilms are displayed together in the figures. We again probe both WT biofilms and biofilms formed from the LCD-locked strain to disentangle contributions from QS from those of c-di-GMP-mediated regulation. We note that the while the regulatory roles of VpsR and VpsT in biofilms are known, the timing with which and location(s) at which they act during biofilm development, to our knowledge, have not been defined.

**Fig. 6.**
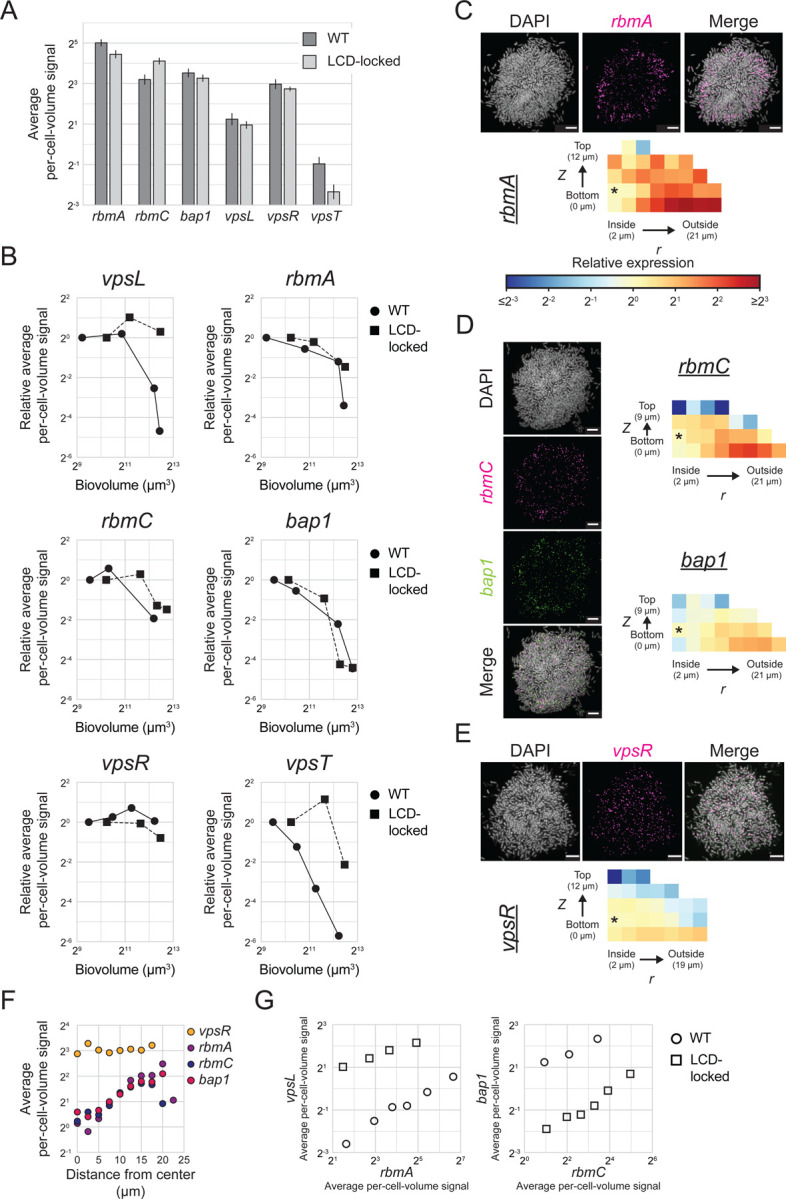
Expression of genes encoding matrix components and regulators over *V. cholerae* biofilm development. (A) Average per-cell-volume fluorescence signal of *rbmA*, *rbmC*, *bap1*, *vpsL, vpsR*, and *vpsT* across *n* = 3–7 replicate WT biofilms or biofilms formed by the LCD-locked strain, for biofilms with the smallest biovolume shown in (B). Error bars represent standard deviations. (B) Relative average per-cell-volume fluorescence signal of *vpsL*, *rbmA*, *rbmC*, *bap1*, *vpsR* and *vpsT* across *n* = 3–7 replicate WT biofilms or biofilms formed by the LCD-locked strain as a function of biovolume. Values are relative to the average per-cell-volume fluorescence signal in biofilms with the smallest biovolume for each background and gene. Pairs of plots displayed next to each other (*vpsL/rbmA*, *rbmA*/*bap1,* and *vpsR*/*vpsT*) represent pairs of genes measured in parallel in the same biofilms. (C) (Top) Confocal microscopy images of DAPI and *rbmA* smFISH fluorescence signal in a representative WT biofilm at the largest biofilm size shown in (B). Images are maximum projections of the first four in-focus *z* slices. Scale bar represents 5 µm. (Bottom) Heatmap of *rbmA* average per-cell-volume fluorescence signal relative to the average per-cell-volume fluorescence signal in the bin denoted with an asterisk are shown as in Fig. 2B for *n* = 5 replicate WT biofilms at the largest biofilm size shown in (B). (D) As in (C) for *rbmC* and *bap1* smFISH fluorescence signals. The color scale bar is as shown in (C). (E) As in (C) for *vpsR* smFISH fluorescence signal. The color scale bar is as shown in (C). (F) Average per-cell-volume fluorescence signal versus distance to the biofilm center, as in Fig. 2C, for the data shown in (C-E). (G) Relationship between average per-cell-volume smFISH fluorescence signal between pairs of genes measured in parallel across groups of cells in WT biofilms and biofilms formed by the LCD-locked strain in (C, D) and (S6A, B), calculated as described in Methods: Correlating gene expression in cell groups. The gene on the *x*-axis represents the reference gene. All data have been corrected using the model described in the text (See Methods: Spatial correction model). See also Fig. S6.

We first established baseline expression values for *rbmA*, *rbmC*, *bap1*, *vpsR*, and *vpsT* in immature biofilms (Fig. 6A). Each gene displayed a similar basal expression level in young WT biofilms and young biofilms of the LCD-locked strain. One could anticipate that the two strains would have similar expression levels given that cells in immature biofilms are in the LCD QS state. *rbmA*, *rbmC*, and *bap1* are all expressed at similar levels, approximately 5-fold higher than *vpsL*. Presumably, *vpsL,* encoding an enzyme, is not required at the quantities needed for matrix structural proteins, at least early in biofilm development. The two regulators also display striking differences in expression, with *vpsR* expressed >15-fold higher than *vpsT*.

We hypothesized that, similar to what occurs for *vpsL* (Fig. 5A), expression of the three matrix-protein encoding genes would decrease over biofilm development. Indeed, decreases do occur in each case as biofilms mature, although the timing and magnitudes of the reductions differ between the three genes (Fig. 6B). In all three cases, the changes that occur in biofilms composed of the LCD-locked strain mirror those that occur in WT biofilms, indicating that their expression timing is regulated by c-di-GMP signaling and not by QS. *rbmC* and *bap1* are controlled by c-di-GMP though VpsR; they are not regulated by VpsT and they are not known to be controlled by QS. *rbmA* is controlled by c-di-GMP through VpsR and by both c-di-GMP and QS through VpsT. Consistent with regulation of *vpsT* by both QS and c-di-GMP, larger decreases in *vpsT* occur in WT biofilms than in biofilms of the LCD-locked strain (Fig. 6B). Given that VpsT controls *rbmA*, it is therefore surprising that the decrease in expression of *rbmA* that occurs is equivalent in the WT and LCD-locked strain (Fig 6B). We account for this unexpected result as follows: we calculate basal expression of *vpsT* to be <1 mRNA copy per cell in both WT biofilms and in biofilms of the LCD-locked strain (Fig. 6A). Likely, there is insufficient VpsT present in each biofilm cell, irrespective of the strain background or biofilm development stage, for it to act as a potent regulator of *rbmA*. Indeed, the role of VpsT in control of *V. cholerae* biofilm development has been primarily studied using a rugose variant that possess higher basal c-di-GMP levels, and consequently higher *vpsT* levels than WT and LCD-locked *V. cholerae* strains^15,48,49^. Thus, our examination of WT *V. cholerae* that possesses native c-di-GMP levels suggests that the role of VpsT in connecting c-di-GMP and QS changes to *rbmA* expression is largely inconsequential. For this reason, below we focus exclusively on the role of VpsR in connecting changes in c-di-GMP levels to changes in target gene-expression in biofilms.

The reductions in *rbmA*, *rbmC*, and *bap1* that occur over biofilm development could be a consequence of decreases in c-di-GMP concentrations, decreases in *vpsR* expression, or both. *vpsR* expression remains constant over the course of biofilm formation in biofilms formed by both the WT and the LCD-locked strain (Fig. 6B). These results suggest that, over the time of *V. cholerae* biofilm development, decreases in c-di-GMP levels occur that lower VpsR activity. Loss of active VpsR, in turn, results in lower expression of the three downstream biofilm matrix genes.

One prediction stemming from our assertion that differences in c-di-GMP levels across biofilms drive the regionally-distinct pattern of *vpsL* expression is that *rbmA*, *rbmC*, and *bap1*, which we find are regulated by c-di-GMP and not QS, should behave similarly. To test this hypothesis, we measured the spatial expression patterns of the three matrix-protein encoding genes. Fortuitously, *rbmA*, *rbmC*, and *bap1* are more highly expressed than *vpsL,* enabling us to probe their spatial expression patterns in WT *V. cholerae* biofilms, which as mentioned, we could not do for *vpsL*. A clear pattern, with higher expression in peripheral cells than in cells at the core, exists for all three matrix genes in WT biofilms (Fig. 6C, D, F). The magnitude of the spatial variability is distinct for each gene, presumably a consequence of differences in the strength of c-di-GMP-mediated regulation to which each gene is subject. The same spatial pattern of expression occurs in biofilms formed by the LCD-locked strain (Fig. S6A, B, D), consistent with exclusive c-di-GMP regulation and no QS input into control of these three genes in either time or space in biofilms.

In addition to unvarying temporal *vpsR* expression (Fig. 6B), uniform *vpsR* expression also occurs across space in WT biofilms (Fig. 6E, F), demonstrating that the regional patterns of expression that exist for the three VpsR target genes are not driven by regional differences in *vpsR* expression. Together, the results support our model that spatial differences in c-di-GMP levels exist across biofilm cells and, by modulating VpsR activity, establish specific spatial gene-expression patterns of target matrix genes. Further supporting the claim that differences in c-di-GMP concentration exist across cells, a positive correlation exists between expression of VpsR target genes, namely between *rbmA* and *vpsL* and between *rbmC* and *bap1,* in biofilms formed by both the WT and the LCD-locked strain (Fig. 6G, S6E, F). These relationships are unlikely to result from extrinsic noise driving global cell-to-cell differences in gene expression, as no such correlation exists for other pairs of genes under study (Fig. S5A, D). Rather, we suggest that the correlation stems from cell-to-cell differences in c-di-GMP concentrations driving cell-to-cell differences in VpsR activity and, in turn, expression of target genes.

### Perturbing c-di-GMP signaling disrupts the spatial patterning of *vpsL* expression in *V. cholerae* biofilms

**Fig. 7.**
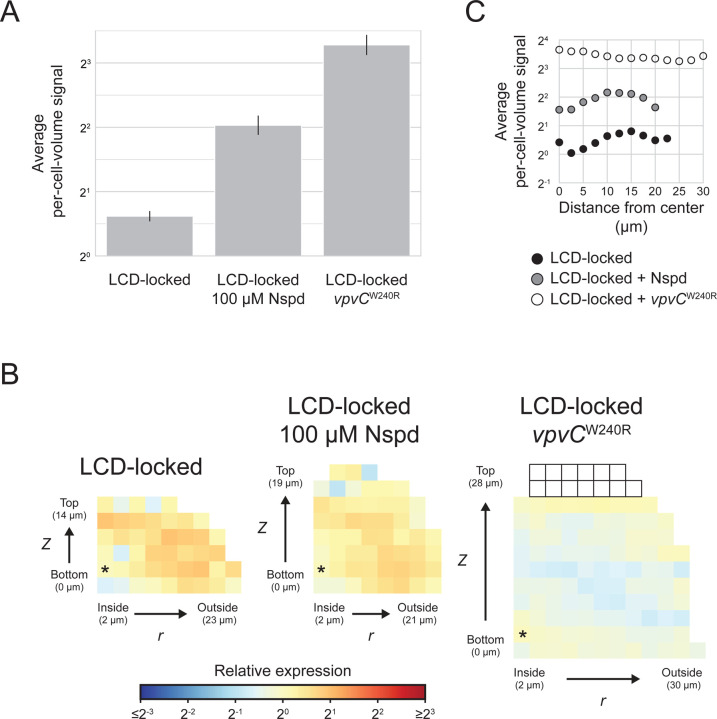
Effect of perturbation of c-di-GMP signaling on the *vpsL* spatial gene expression pattern. (A) Average per-cell-volume fluorescence signal of *vpsL* across *n* = 5 replicate biofilms formed by the LCD-locked strain, LCD-locked strain grown with 100 µM Nspd, or LCD-locked, *vpvC*^W240R^ strain. Error bars represent standard deviations. (B) Heatmaps of *vpsL* average per-cell-volume fluorescence signal relative to the average per-cell-volume fluorescence signal in the bins denoted with asterisks are shown as in Fig. 2B for *n* = 5 replicate biofilms formed by the LCD-locked strain (left), LCD-locked strain grown with 100 µM Nspd (center), or LCD-locked, *vpvC*^W240R^ strain (right). (C) Average per-cell-volume fluorescence signal versus distance to the biofilm center, as in Fig. 2C, for the data shown in (B). All data have been corrected using the model described in the text (See Methods: Spatial correction model).

To bolster our hypothesis that variations in c-di-GMP levels across *V. cholerae* biofilms are responsible for patterning matrix gene expression, we perturbed the endogenous c-di-GMP levels and investigated whether that altered matrix gene-expression patterns. We modulated c-di-GMP levels in biofilms by two mechanisms. First, we grew *V. cholerae* biofilms in the presence of 100 µM Nspd, which, as discussed in the Introduction, increases c-di-GMP by modulating the activity of MbaA (Fig. 1A). Second, we exploited a mutant *vpvC* gene, denoted *vpvC*^W240R^ and often referred to as the rugose mutation, that confers constitutive VpvC DGC activity.^50^ Both Nspd addition and the *vpvC*^W240R^ mutation have been shown previously to increase cytoplasmic c-di-GMP levels, and correspondingly, matrix gene expression, with the *vpvC*^W240R^ mutation being the more potent activator of c-di-GMP production.^51^ Consistent with these earlier findings, we show that *vpsL* expression is highest in biofilms formed by the LCD-locked strain containing the *vpvC*^W240R^ mutation, displays intermediate expression in biofilms of the LCD-locked strain to which Nspd has been administered, and is lowest in biofilms of the LCD-locked strain with unperturbed, endogenous c-di-GMP levels (Fig. 7A). The spatial pattern in which cells at the biofilm periphery display higher *vpsL* expression than cells at the core, while less dramatic than in the LCD-locked strain, remains present in biofilms of the LCD-locked strain following treatment with Nspd (Fig. 7B, C). However, further increasing c-di-GMP levels through introduction of the *vpvC*^W240R^ mutation eliminates the *vpsL* spatial pattern (Fig. 7B, C). These results suggest the following model: Globally increasing c-di-GMP levels overrides the naturally occurring differences in c-di-GMP concentrations that exist in *V. cholerae* cells in biofilms. Consequently, the c-di-GMP-controlled pattern of *vpsL* gene expression is lost.

Differences in spatial *vpsL* expression patterns in biofilms of the AI-2 sensor strain grown in the presence of Nspd and in the absence or presence of AI-2 confirm the above mechanistic interpretation for how the *vpsL* biofilm spatial pattern is established. Regarding mechanism, HapR activates transcription of *nspS*-*mbaA* encoding the Nspd receptor-effecter pair. Consequently, at HCD (i.e. +AI-2), when HapR is abundant, Nspd potency increases due to increased NspS-MbaA-mediated detection. Under this condition, MbaA DGC activity increases to a higher level than when Nspd is supplied in the absence of AI-2.^46^ Consistent with this mechanism, in biofilms of the AI-2 sensor strain grown with Nspd but without AI-2, *vpsL* is preferentially expressed in peripheral biofilm cells. By contrast, cells of the AI-2 sensor strain in biofilms grown with Nspd and AI-2 display uniform *vpsL* expression across space (Fig. 3D, E). Again, these results show that globally increasing c-di-GMP in *V. cholerae* biofilms abolishes the c-di-GMP-directed *vpsL* spatial gene-expression pattern. Moreover, the mechanism by which the c-di-GMP levels are altered is not relevant.

## Discussion

In this study, we present a biofilm-specific smFISH technology that enables accurate quantitation of spatiotemporal gene-expression patterns at single-cell resolution and across biofilm development. This technology satisfies an urgent need in the biofilm field by providing the means to probe both the mechanistic underpinnings that drive the formation of global and regional biofilm architectures and the determinants that specify individual cell fates in biofilms. Our quantitative approach can, in principle, be applied to any gene of interest, including genes that have only low levels of expression. Our approach does not provide the throughput of other smFISH strategies such as seqFISH; however, it benefits from high accuracy and individual cell resolution, expanding the types of research questions one can address. One drawback of smFISH is that it must be performed on fixed samples, which limits the temporal resolution obtainable in a single experiment. Despite this shortcoming, in this work, we were nonetheless able to reveal previously unknown spatial and temporal gene-expression patterns in *V. cholerae* biofilms. The smFISH signal decays substantially as a function of *z*, which imposes an upper bound on the thickness of biofilm samples that can be assayed (25–30 µm). In the context of the present study, *V. cholerae* cells disperse from biofilms before the communities reach 25 µm, so this issue was not a limitation for our analyses. Modifications to this technology will be required to probe thicker, non-dispersing samples.

We used spatiotemporal expression patterns of genes specifying *V. cholerae* biofilm regulators and matrix components as our test case for the smFISH technology. Our analyses reveal new information concerning how these elements are regulated in space and time. Importantly, much of the previous work on *V. cholerae* biofilm formation exploited conditions or mutants that prevented biofilm dispersal and, in so doing, interfered with native QS and/or c-di-GMP signaling. These earlier investigations were seminal in that they identified key structural and regulatory components in *V. cholerae* biofilms, but by necessity, they prohibited study of endogenous signal transduction dynamics. While we did not focus on the biofilm dispersal process per se in this first work, our approach enabled us to study WT *V. cholerae* cells grown such that important signaling molecules that drive biofilm establishment and dispersal could vary, presumably as they do during biofilm growth in nature and during infection. Indeed, under our conditions, at late times, cells do disperse from biofilms. Our results suggest that as biofilms reach maturity, expression of genes specifying matrix components must cease in order for cells to become capable of dispersal. *V. cholerae* cells use two mechanisms to accomplish this transition. In the case of genes that are QS-repressed at HCD, namely the *vpsII* operon-encoded VPS biosynthetic genes, the timing and strength of repression is determined predominately by the HapR QS master regulator. As biofilm cells transition from the LCD to the HCD QS state, *hapR* expression increases, and *vpsII* expression is repressed. By contrast, for non-QS controlled matrix genes, the key player is c-di-GMP. Specifically, expression of genes encoding the three major matrix proteins, *rbmA*, *rbmC*, and *bap1*, is regulated exclusively by changes in c-di-GMP levels that control the activity of the transcriptional activator VpsR. For these genes, as biofilms mature, *vpsR* expression remains constant, but decreases in c-di-GMP levels result in decreased VpsR activity, and consequently suppression of the target genes.

The timing of gene expression changes differs among the matrix genes. *bap1* expression is suppressed more strongly and earlier in the biofilm lifecycle than are *rbmA* and *rbmC*. Bap1 is important for cell-surface interactions, while RbmA and RbmC are needed for continued cell-cell and cell-matrix contacts. One possible explanation for the timing differences is that Bap1 completes its primary function early in biofilm formation, such that at later times in biofilm development, lower concentrations of Bap1 suffice. Differences in the strength of regulation by c-di-GMP, through VpsR, between the promoters driving these genes likely contribute to these temporal distinctions. Future work is required to confirm this hypothesis and further connect gene expression timing to distinct biofilm developmental events.

The smFISH approach allows measurements at single-cell resolution, revealing that expression of genes encoding all the matrix components studied here is higher in cells residing at the biofilm periphery than in cells in the interior. Our data suggest this pattern is a consequence of differences in c-di-GMP levels across the biofilm, with higher c-di-GMP levels in peripheral cells driving higher VpsR activity and, thus, higher matrix component gene expression. Unfortunately, we are unable to directly measure differences in c-di-GMP concentrations in biofilm cells to bolster our mutant and imaging data because currently available reporters of c-di-GMP levels^52^ are not sufficiently sensitive. However, in support of our model, our results show that this inside-to-outside spatial pattern does not depend on QS, as the pattern is preserved in biofilms of the QS-deficient, LCD-locked strain. QS is the only known regulatory pathway other than c-di-GMP signaling that feeds into control of matrix genes. Additionally, we can completely eliminate the matrix gene-expression spatial pattern by perturbing c-di-GMP levels through a variety of mechanisms.

Local differences in c-di-GMP levels that drive particular spatial and temporal patterns of biofilm matrix component production could be key to appropriately distributing tasks among individual cells in biofilm communities. We suggest that, as the biofilm matures, cells at the core have already become surrounded by extracellular matrix and so there is no need for those cells to continue to produce matrix to remain members of the biofilm community. By contrast, cells at the outside edge of the biofilm must produce and secrete matrix components to become or stay bound to the community. Further work, perhaps combining smFISH with matrix protein labeling, could connect our current findings concerning gene expression to distributions of secreted components in biofilms. Most of what is known about biofilm matrix component localization comes from field-founding studies of the rugose variant and derivatives of it^12^. As mentioned, in the rugose (VpvC^W240R^) *V. cholerae* strain, uniform expression of *vpsL* occurs across biofilms. Thus, comparisons of our data with previous findings will not yield the needed connections between gene expression and regional protein localization in biofilms, and additional studies will be necessary. Analyses focusing on times beyond those assessed in this work to monitor cells actively undergoing biofilm dispersal are also required to understand if and how spatiotemporal patterns of matrix gene expression trigger this final phase of the biofilm lifecycle.

A key finding here is that bacterial cells residing only a few microns apart experience environments sufficiently different to promote distinct modulation of their c-di-GMP levels. We currently do not know what external cue or cues are responsible for these differences. In addition to the stimuli known to modulate the activity of c-di-GMP metabolizing enzymes, for example norspermidine, sugars, and amino acids, the stimuli to which the vast majority of c-di-GMP producing/degrading enzymes respond remain unknown.^18,53,54^ Thus, understanding the origin of the c-di-GMP-driven spatial patterning of matrix biofilm genes remains mysterious. Nonetheless, our smFISH approach, by enabling the spatiotemporal quantitation of genes encoding regulators, enzymes, and structural proteins provides a new and hopefully important tool for addressing questions concerning gene-expression patterns in three-dimensional living, growing bacterial communities.

## Methods

### Resource Availability

#### Lead contact

Further information and requests for resources and reagents should be directed to and will be fulfilled by Dr. Bonnie L. Bassler (bbassler@princeton.edu)

#### Materials availability

Strains and reagents used in this study are available upon request from Dr. Bonnie L. Bassler.

#### Data and code availability

- Raw images will be made available prior to publication
- Original code used in this study will be made available prior to publication
- Any additional information required to reanalyze the data reported in this paper is available from the lead contact upon request.

### Experimental Model and Subject Details

#### Bacterial strains and growth conditions

The parent *V. cholerae* strain used in this study is O1 El Tor biotype C6706str2 ∆*vpsS* ∆*cqsR* (BB_Vc416). All strains used in this study are listed in the Key Resources Table. *V. cholerae* biofilms were grown as follows: Cultures were seeded from single colonies into 1 mL LB and grown at 37 °C for 3 h with shaking, to an approximate OD_600_ = 0.1. Cultures were back diluted to an OD_600_ = 6 x 10^-6^ in M9 medium (1× M9 salts, 100 μM CaCl_2_, 2 mM MgSO_4_, 0.5% dextrose, and 0.5% casamino acids). 200 µL of cultures were dispensed onto No. 1.5 glass coverslip bottomed 96-well plates (MatTek, Ashland, MA, USA), and grown statically at 37 °C. When indicated, 0.0375%, or 0.2% arabinose, 100 µM norspermidine, and/or 10 µM AI-2 was added to LB and M9 medium. When AI-2 was provided, media also contained 0.1 mM boric acid. In every case in which data for planktonic cultures are reported, the cultures were grown in the identical medium as that used for biofilm growth and the same cultures used to seed biofilm growth were used to seed planktonic cultures. Cultures were back diluted to OD_600_ = 0.0002 in 10 mL M9 medium and grown with shaking at 37 °C.

### Method Details

#### Single-molecule RNA FISH (smFISH) probe design and hybridization

Custom Stellaris RNA FISH probes were designed against genes of interest using the Stellaris RNA FISH Probe Designer v.4.2 (LGC, Biosearch Technologies). Probes were labeled with Quasar 670, CAL Fluor Red 590, or FAM (see Table S1 for a complete list of probe sequences and associated fluorophores). For biofilm samples, probes were hybridized following the manufacturer’s instructions for adherent cells in 96-well glass bottom plates, with minor modifications. The full protocol is available online at www.biosearchtech.com/stellarisprotocols. Briefly, following growth, medium was decanted, and adhered biofilms were fixed for 10 min at room temperature with 200 µL 1x PBS and 3.7% formaldehyde. The fixed biofilms were washed four times with 200 µL 1x PBS. To permeabilize cells, the samples were incubated overnight, or up to 24 h, at 4 °C in 200 µL 70% EtOH. Samples were incubated for 5 min at room temperature with 200 µL RNA FISH wash buffer A (Biosearch Technologies, prepared following the manufacturer’s instructions) containing 10% formamide. Probes were hybridized with 75 µL RNA FISH hybridization buffer (Biosearch Technologies) containing 10% formamide and 1.5 µL of each probe stock (12.5 µM), followed by overnight incubation at 37 °C. Samples were washed twice for 30 min at 37 °C with 200 µL RNA FISH wash buffer A containing 10% formamide. Cells were stained with 50 µg mL^–1^ DAPI in wash buffer A for 20 min at 37 °C. Following a final wash with 200 µL Stellaris RNA FISH wash buffer B, 50 µL VectaShield mounting medium was added, and the samples were immediately imaged. Regarding planktonic cells, probe hybridization was performed as previously described^38^, with minor modifications as detailed in ^55^.

#### Confocal imaging

Imaging was performed with a Nikon Eclipse Ti2 inverted microscope equipped with a Yokogawa CSU-W1 SoRa confocal scanning unit. Samples were imaged with a CFI Apochromat TIRF ×60 oil objective lens (Nikon, 1.49 numerical aperture) with excitation wavelengths of 405 (DAPI), 488 (mNG and FAM), 561 (mScarlet and CAL Fluor Red 590), and 640 nm (Quasar 670) and with 0.5 µm *z*-steps. Images were captured through a ×2.8 SoRa magnifier.

#### smFISH image analysis

Images were processed using Nikon NIS-Elements Denoise.ai software. Denoised images of planktonic cells were segmented using Fiji software^56^ from maximum *z*-projections of the DAPI channel. A gaussian blur was applied to images, and cells were identified using an intensity threshold. Cells that could not be accurately resolved from neighboring cells were excluded from downstream analyses. Using the DAPI channel, denoised images of biofilm cells were segmented with BiofilmQ into cubes with side lengths of 2.32 µm.^57^ The center *x, y, z* coordinates of each cell cube and the volume fraction of each cube occupied by cell mass were exported for use in downstream analyses. We interchangeably use “cell cube” and “cell” to refer to this resulting cube-based segmentation.

smFISH data were analyzed using custom python scripts. Briefly, images were convolved with a Gaussian function to remove noise. Puncta were detected as local maxima with intensities greater than a threshold that was established based on a set of negative control images specific to each probe set. For *hapR* and *mNG* probes, negative control images were acquired using biofilms containing cells lacking *hapR* and *mNG* (BBVc_801). For *vpsL* probes, mature WT biofilms (BBVc_416), i.e., at a stage when *vpsL* is no longer expressed, were hybridized with *vpsL* to acquire negative control data. The *vpsR, vpsT, rbmA, rbmC,* and *bap1* genes could not be deleted, because without them, biofilms do not form. Thus, as a negative control for these probe sets, WT (BBVc_416) biofilms, i.e. biofilms lacking *mNG*, were hybridized with *mNG*-targeting probes labeled with the fluorophore matching each probe set of interest. Each punctum was fitted with a 3D Gaussian function to determine the integrated, background-subtracted punctum intensity. In instances with multiple puncta residing in close proximity, a 3D multi-Gaussian fit was performed. Puncta were assigned to cells (planktonic samples) or to cell cubes (biofilm samples) and the total fluorescence signal per cell or cube was calculated as the sum of all puncta intensities. For biofilm samples, this fluorescence signal was further normalized by the Cube_VolumeFraction, which is the fraction of the cube volume occupied by biomass, calculated by BiofilmQ.

#### Spatial correction model

Spatial correction factors were calculated using data from the largest biofilms formed by the parent strain carrying ∆*vc1807*::pBad-*mNG* (BB_Vc813) imaged under each condition. Unique correction factors were derived for each of the three fluorophores, at both high and low expression levels of *mNG*, and for all positions (*r, z*). As biofilms are not perfect half spheres, the *r* position of each cell cube was calculated in a *z*-dependent manner. Specifically, a unique center position was determined for each *z* bin as the volume-weighted average *x*, *y* coordinate across all cells within that *z* bin. For *z* bins with *z* ≤ 391 pixels (15.2 µm) and *z* > 391 pixels (15.2 µm), this procedure shifted the calculated center position relative to the *x, y* center of the biofilm as a whole by an average of 4.8 µm (SD = 2.6 µm) and 13.0 µm (SD = 5.6 µm), respectively. The *r* position for each cell cube was subsequently calculated as the distance between the cell cube’s *x, y* location and the center *x, y* position corresponding to that cell cube’s *z* location. The correction factors use experimental data to describe both *z* and *r* positional biases, as detailed below. To calculate *z* bias, the average per-cell-volume smFISH fluorescence signals from cell cubes with radial position *r* = 311 pixels (12.1 µm) were calculated as a function of cell cube *z* position and normalized to the average per-cell-volume smFISH fluorescence signal of cell cubes with *z, r =* 91, 311 pixels (3.55, 12.1 µm). These data were fit with the ad-hoc Equation (1) to determine the correction factor as a function of *z*:

(1) $$\text{Per-cell-volume fluorescence correction }=ae^{\frac{-z}{b}}+\left(1-a\right)e^{\frac{-z}{c}}.$$

To calculate *r* bias, the average per-cell-volume fluorescence signal of cell cubes with *z* position *z* = 91 pixels (3.55 µm) were calculated as a function of cell cube *r* position. We observed that the average smFISH fluorescence signal decreased linearly as a function of *r* before plateauing at *r* = *r*_*i*_, with the position of *r*_*i*_ differing for each sample. For each of the six samples, we chose an *r*_*i*_ value by visual inspection of the data. The average smFISH fluorescence signal was normalized to the average smFISH fluorescence signal of cell cubes with *r* = *r*_*i*_, *z* = 91 pixels (3.55 µm) and fit to the ad-hoc Equation 2:

(2) $$\text{Per-cell-volume fluorescence correction }=\left\{\begin{array}{c} a_{r}r+b_{r},r\leq r_{i}\\ a_{r}r_{i}+b_{r},r>r_{i} \end{array}\right..$$

Using the specific fit parameters for each fluorophore and for high and low gene-expression levels, the correction factor for all positions (*r, z*) was calculated for each condition as the product of Equation (1) and Equation (2). These values form the basis for calculating interpolative equations used to correct all datasets, as described below.

In order to acquire appropriate correction factors for smFISH fluorescent signals from genes with any expression level, not only those matching the *mNG* high and low expression conditions, and also for biofilms of any size, we developed a generalized interpolation model as follows: for a given fluorophore, for each position (*r, z*), a linear equation *Y*(*X*) connecting points (*X*_1_, *Y*_1_) and (*X*_2_, *Y*_2_) was calculated, in which *Y*_1_ and *Y*_2_ are the correction factors derived above from the low- and high-gene-expression conditions, respectively, and *X*_1_ and *X*_2_ are the log_10_ values of the average summed smFISH fluorescence signals across the biofilms that were used to calculate the above correction factors in the low- and high-gene-expression conditions, respectively. With this model, for any given biofilm or group of replicate biofilms, unique correction factors can be determined for each position (*r, z*) based on the total smFISH fluorescent signal in the biofilm(s). Specifically, for each position (*r, z*), the log_10_ of the average summed smFISH fluorescence signals across replicate biofilms was taken as an *X* value and introduced into the corresponding (*r, z*)- dependent linear equation *Y*(*X*) generated above to calculate an (*r, z*)-specific correction factor for those biofilms. To use these calculated correction factors, for each cell cube within the biofilm, the per-cell-volume smFISH fluorescence signal, calculated as described above, was divided by the correction factor based on its position (*r, z*). The resulting calculated values were used for all subsequent analyses, unless otherwise noted. Values for all fit parameters, (*r, z*)-dependent correction factors, and resulting (*r, z*)-dependent interpolative linear equations can be found in Table S2. The code detailing generation and application of the model, and accompanying sample plots of fit parameters, will be deposited and available prior to publication.

#### Analysis of spatial gene-expression patterns at single-cell resolution

Spatial gene-expression patterns in individual cells were assessed in two ways. First, cell cubes across replicate biofilms were grouped by their center *r* and *z* positions. Radial positions for each cell cube were calculated as described above. The average per-cell-volume fluorescence signal for each group of cells was calculated and normalized to the group of cells with center position *r, z* = 91, 91 pixels (3.5 µm, 3.5 µm). Resulting data for groups containing ≥ 25 cells were plotted as heatmaps using bins with Δ*r,* Δ *z* = 2.32, 2.32 µm. Second, the distance of each cell cube from the center of the biofilm was calculated from the center *r, z* position of that cell cube to the center position *r, z* = 0, 91 pixels (0, 3.5 µm). Based on this calculated distance, cells across replicate biofilms were grouped into 2.5 µm sized bins, and the average per-cell-volume fluorescence signal per bin was calculated. Resulting data were plotted as a function of the left boundary of each bin.

#### Correlating gene expression in cell groups

The relationship between gene-expression levels measured in parallel across cells was assessed as follows: For a given ‘reference gene’, all cells with zero signal corresponding to that gene were omitted from analysis. The remaining cells were ordered by expression level of the reference gene and grouped into bins of 500 cells each. The average per-cell-volume fluorescence signal was calculated for each bin for both the reference gene and a second ‘comparison gene’, and these two average signal values were plotted against one another for each bin.

#### Quantitation and statistical analyses

Standard deviations were calculated using custom Python scripts from log_2_ transformed data. *z*-scores of average per-cell-volume fluorescence signal in *r, z* bins were calculated using the following formula, $\frac{{\bar{x}}_{i}-{\bar{x}}_{c}}{S}$where ${\bar{x}}_{i}$ represents the mean per-cell-volume fluorescence signal across cells in the bin, Sis the standard deviation of the per-cell-volume fluorescence signal across cells in the bin, and ${\bar{x}}_{c}$ is the mean per-cell-volume fluorescence signal in the bin with *r, z* = 91, 91 pixels (3.5 µm, 3.5 µm).

## Supplementary Material

Supplement 1 **Table S1.** smFISH probe sequences, related to STAR Methods.

Supplement 2 **Table S2.** Values of fitting and correction parameters generated by the spatial correction model, related to STAR Methods.

Supplement 3 **Data S1.** Companion plots generated from non-corrected data, related to Fig. 3–7, S1, S3, and S5.

Supplement 4 **Fig. S1. Confocal microscopy biases regarding constitutive fluorescent reporters and stains, related to** Figure 2. (A) Maximum projection confocal microscopy images of mScarlet and mNG fluorescence over time in a representative *V. cholerae* biofilm harboring pTac-*mScarlet* and pTac-*mNG*. (B) Signal from DAPI staining in the first in-focus *z* slice and *xz* and *yz* cross sections of a mature *V. cholerae* biofilm. All scale bars represent 5 µm.
**Fig. S2. Validation of fluorescent signal quantitation in *V. cholerae* biofilms, related to** Figure 2. (A) Average per-cell-volume *mNG* smFISH fluorescence signal measured in *V. cholerae* planktonic and biofilm cells. 0.2% or 0.0375% arabinose was provided, as indicated by shaded blue boxes denoted High and Low Expression, respectively. 100 µM Nspd was included in all cases. *mNG* expression was measured by smFISH using probes labeled with one of three fluorophores, as indicated. Values for biofilm cells represent the mean normalized per-cell-volume fluorescence signal across *n* = 10–12 biofilms. The small, medium, and large circular symbols represent biofilms with approximate biovolumes of 2^9^, 2^11^, and 2^14^ µm^3^, respectively. Error bars denote standard deviations, which are in some cases smaller than the sizes of the symbols used in the plots. Values for planktonic cells represent the average across all cells in single replicate experiments; error bars are excluded. (B) Probability density curves of the distributions of individual smFISH puncta intensities for the *mNG* transcript measured under the Low Expression condition, *hapR* measured at LCD, and *qrr*4 and *vpsL* measured at HCD. The peaks of the probability curves were used to estimate the intensities of a single mRNA molecule for each transcript. The minimum (0.5) and maximum (1.7) puncta intensities are highlighted with vertical lines. (C) Heatmaps as in Fig. 2A of the main text showing the relative corrected data for the three fluorophores for biofilms of additional sizes. Data represent *n* = 10–12 replicate biofilms. (D) As in Fig. 2C of the main text, for the data shown in (C). Black symbols represent biofilms in the left column and white symbols represent biofilms in the right column of (C).
**Fig. S3. Validation of temporal and spatial QS gene expression patterns in *V. cholerae* biofilms, related to** Figure 3. (A) Representative images of DAPI signal, and *qrr*4 (*mNG*), *hapR*, and *vpsL* smFISH fluorescence signals in biofilms of varying sizes grown without (left) or with (right) 10 µM AI-2. All biofilms were grown with 100 µM Nspd. Images represent maximum projections of the first four in-focus *z* slices. Scale bars represent 5 µm. (B, C) The non-corrected data used to generate the spatially corrected fluorescence signal values shown in Fig. 3D, E, respectively.
**Fig. S4. Spatial QS gene expression patterns across biofilm sizes, related to** Figure 3. (A) Heatmaps showing the relative *qrr*4 (*mNG*), *hapR*, and *vpsL* per-cell-volume fluorescence signals as in Fig. 3D of the main text for additional biofilm sizes. *n* = 9–10 replicate biofilms. (B) Average per-cell-volume fluorescence signal versus distance to the biofilm center is shown, as in Fig. 3E of the main text, for the data represented in (A). Light symbol coloring for +AI-2 samples represents the smaller biofilms (left in (A)) and dark symbol coloring for +AI-2 samples represents larger biofilms (right in (A)). All data have been corrected using the model described in the text (See Methods: Spatial correction model).
**Fig. S5. Relationship between *qrr*4, *hapR*, and *vpsL* expression across biofilm cells, related to** Figs. 4, 5. (A) (Left) Average *qrr*4 (*mNG*) per-cell-volume fluorescence signal as a function of *hapR* per-cell-volume fluorescence signal for groups of cells in WT biofilms and biofilms of the LCD-locked strain shown in Fig. 4C. Values calculated as described in Methods: Correlating gene expression in cell groups, using *qrr*4 as the reference gene. (Right) As on the left, with *hapR* as the reference gene. (B) As in (A) for additional biofilm sizes. Dark to light shading represents biofilms of decreasing size. (C) The relationship between the coefficient of variation (CV) for *qrr*4 (*mNG*) or for *hapR* per-cell-volume fluorescence signal across cells in individual biofilm or planktonic samples, as indicated, as a function of the average *qrr*4 (*mNG*) or *hapR* per-cell-volume signal in the sample. Empty symbols represent biofilms of the WT (circles) or of the LCD-locked strain (squares) (data from Fig. 4). Slopes of the best fit lines for *qrr*4 and *hapR* for these samples are −0.42 and −0.17, respectively. Filled colored symbols represent planktonic samples of the AI-2 sensor strain grown with (circles) or without (squares) AI-2 (planktonic data from Fig. 3). Slopes of the best fit lines for *qrr*4 and *hapR* for these samples are −0.43 and −0.37, respectively. Black points represent both *qrr*4 (*mNG*) and *hapR* expression in biofilms of the AI-2 sensor strain grown with or without AI-2 (biofilm data from Fig. 3). Slopes of the best fit lines for *qrr*4 and *hapR* for these samples are −0.28 and −0.37, respectively. (D) As in (A), comparing *vpsL* and *hapR* per-cell-volume fluorescence signal across cells groups. Left, *hapR* used as reference gene. Right, *vpsL* used as reference gene. All data have been corrected using the model described in the text (See Methods: Spatial correction model).
**Fig. S6. Spatial patterns of genes encoding matrix components in biofilms of the LCD-locked *V. cholerae* strain, related to** Fig. 6. (A) (Left) Confocal microscopy images of DAPI and *rbmA* and *vpsL* smFISH fluorescence signal in a representative biofilm made from the LCD-locked strain at the largest biofilm size shown in Fig. 6B. Images are maximum projections of the first four in-focus *z* slices. Scale bar represents 5 µm. (Right) Heatmap of *rbmA* and *vpsL* average per-cell-volume fluorescence signal relative to the average per-cell-volume fluorescence signal in the bin denoted with an asterisk are shown as in Fig. 6C for *n* = 5 replicate biofilms formed by the LCD-locked strain at the largest biofilm size shown in Fig. 6B. (B) As in (A) for *rbmC* and *bap1* smFISH fluorescence signal. *n* = 5 replicate biofilms. (C) As in (A) for *vpsR* smFISH fluorescence signal. *n* = 6 replicate biofilms. (D) Average per-cell-volume fluorescence signal versus distance to the biofilm center, as in Fig. 6F, for the data shown in (A-C). (E) As in Fig. 6G, using the reciprocal gene, as indicated on the *x*-axis, as the reference gene. (F) As in in Fig. 6G for additional biofilm sizes. Dark to light shading represents biofilms of decreasing sizes. All data have been corrected using the model described in the text (See Methods: Spatial correction model). Regarding panel C, *vpsR* appears to be preferentially expressed in peripheral cells. The core regions of these biofilms contained fewer cells than all other biofilms analyzed in this work and consequently, there was low *vpsR* smFISH fluorescence signal output from the core. That feature biased the quantitation making it seem a spatial pattern exists.

## Figures and Tables

**Table:** Key Resources Table

REAGENT or RESOURCE	SOURCE	IDENTIFIER
Bacterial and virus strains		
*Vibrio cholerae* C6706; ∆*vpsS* ∆*cqsR* ∆*Vc1807*::Kan^R^	Bridges et al., (2019)	BB_Vc416
BB_Vc416; ∆*Vc1807::*pTac*-mScarlet; ∆lacIZ::*pTac*-mNG*	This work	GJ022
BB_Vc416; ∆*Vc1807*::pBad-*mNG*	This work	BB_Vc813
BB_Vc416; ∆*lacIZ*::p*qrr*4-*mNG* ∆*Vc1807*::Cm^R^	Bassler Lab Collection	BB_Vc403
BB_Vc416; ∆*cqsS* ∆*luxS* ∆*lacIZ*::p*qrr*4-*mNG* ∆*Vc1807*::Spc^R^	This work	BB_Vc806
BB_Vc416; *luxO D61E* ∆*lacIZ*::p*qrr*4-*mNG* ∆*Vc1807*::Spc^R^	This work	BB_Vc807
BB_Vc416; ∆*hapR* ∆*Vc1807*::Spc^R^	This work	BB_Vc801
BB_Vc416; *luxO D61E* ∆*Vc1807*::Kan^R^	This work	BB_Vc804
BB_Vc416; *luxO D61E vpvC W240R* ∆*Vc1807*::Kan^R^	This work	BB_Vc811
Chemicals, peptides, and recombinant proteins		
4,5-Dihydroxy-2,3-pentanedione (DPD)	Jubilant Biosys	Custom synthesis
Bis(3-aminopropyl)amine	Sigma-Aldrich	Cat#I1006-100G-A
Stellaris® RNA FISH Hybridization Buffer	LGC Biosearch Technologies	Cat#SMF-HB1-10
Stellaris® RNA FISH Wash Buffer A	LGC Biosearch Technologies	Cat#SMF-WA1-60
Stellaris® RNA FISH Wash Buffer B	LGC Biosearch Technologies	Cat#SMF-WB1-20
Formaldehyde 37% in aqueous solution, microfiltered	VWR	Cat#100503-912
Formamide	Millipore	Cat#4610-100ML
Deposited data		
Raw images	This work	Will be deposited prior to publication
Oligonucleotides		
smFISH probes	This work	See Table S1
Software and algorithms		
Original code (model generation, image analysis, quantification)	This work	Will be deposited prior to publication
BiofilmQ	Hartmann et al., (2021)	https://drescherlab.org/data/biofilmQ/docs/
Fiji	Schneider et al., (2012)	https://imagej.net/software/fiji/
Python version 3.11.7	Python Software Foundation	https://www.python.org/downloads/release/python-3117/
