# Supplementary figures and images for: Cell-scale gene-expression measurements in Vibrio cholerae biofilms reveal spatiotemporal patterns underlying development
Source: bioRxiv. 2025 Jan 16:2024.07.17.603784. Originally published 2024 Jul 17. Preprint. [Version 2] doi: 10.1101/2024.07.17.603784 (PMC11275835; doi:10.1101/2024.07.17.603784)

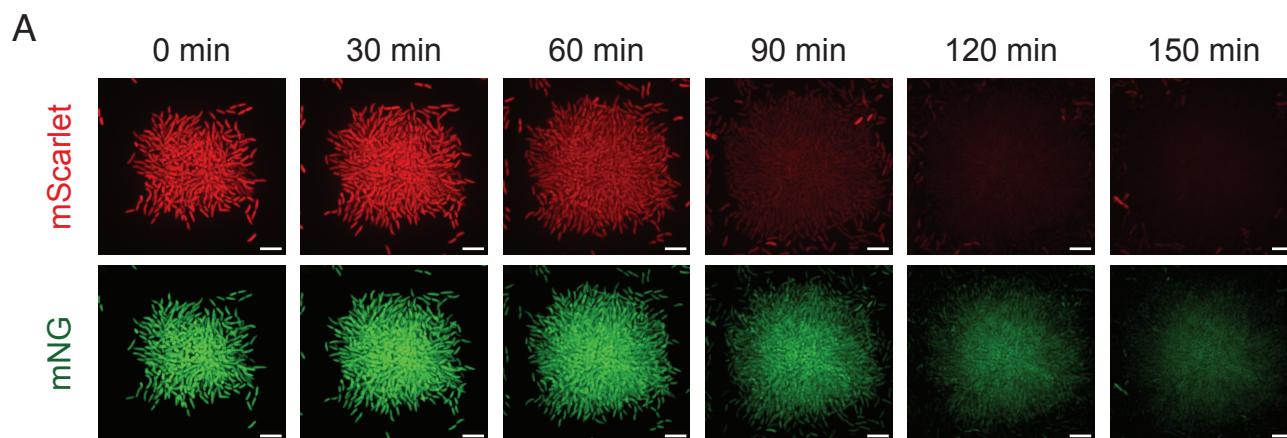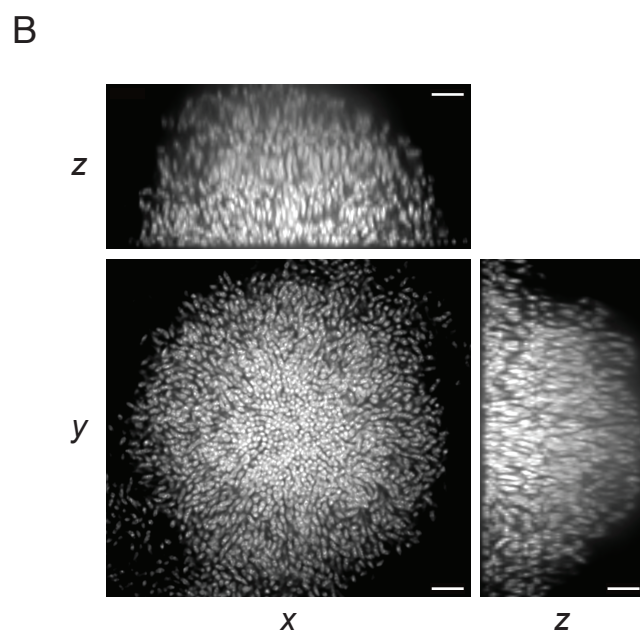

A

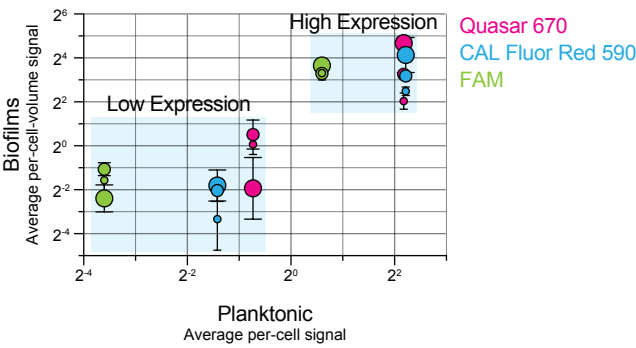

C

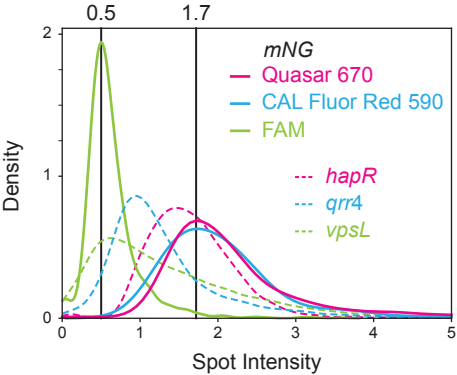

B

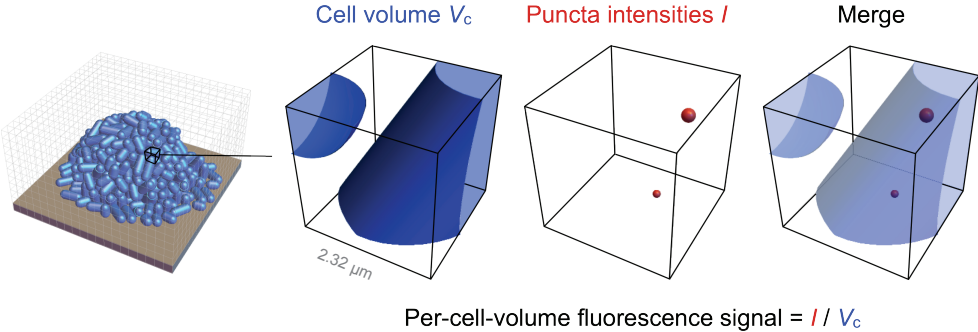

D

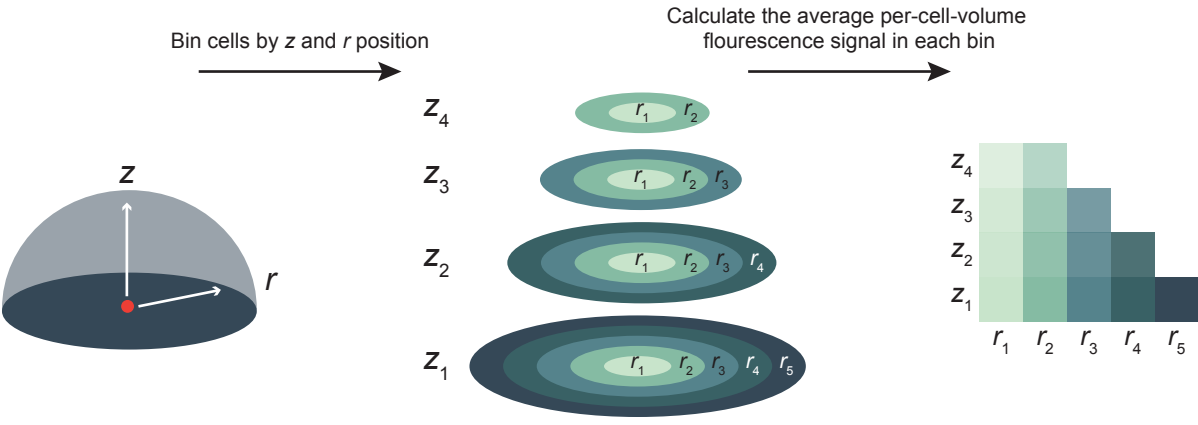

A

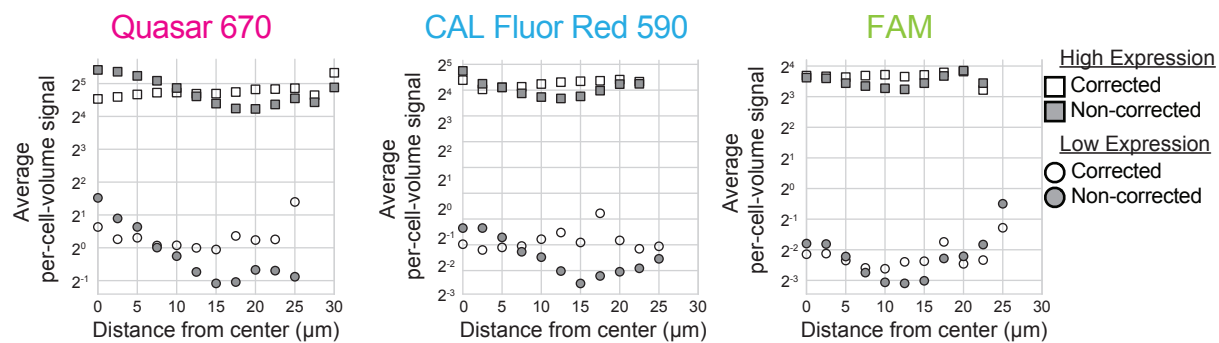

B

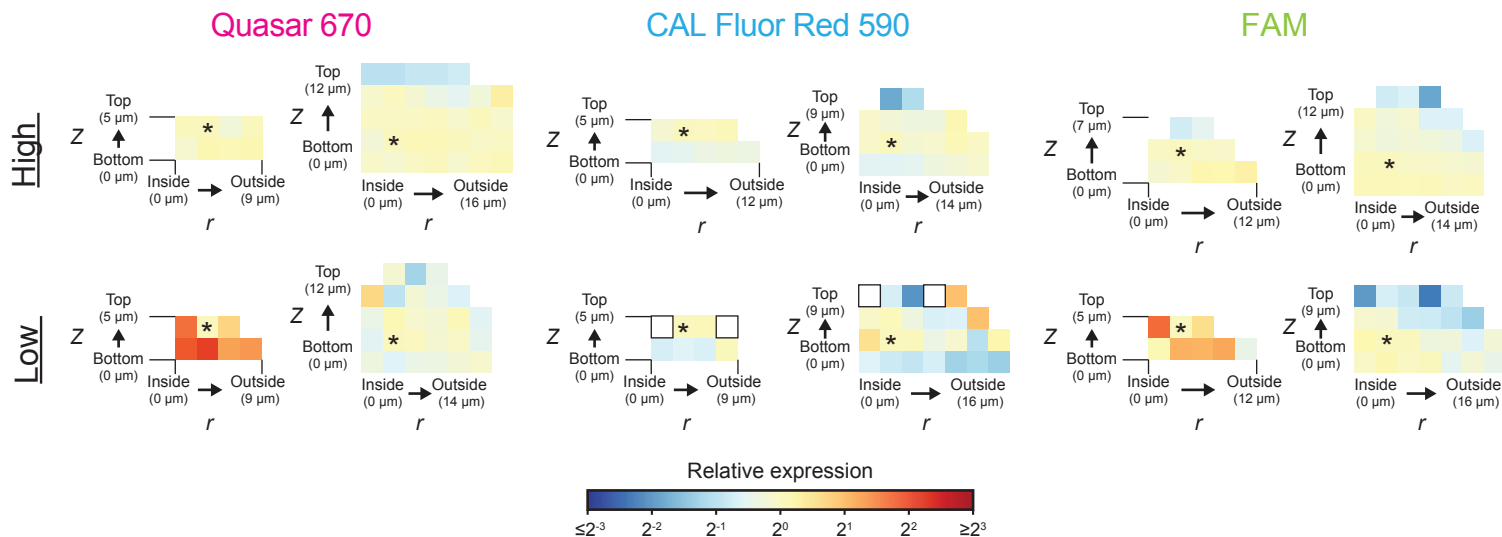

C

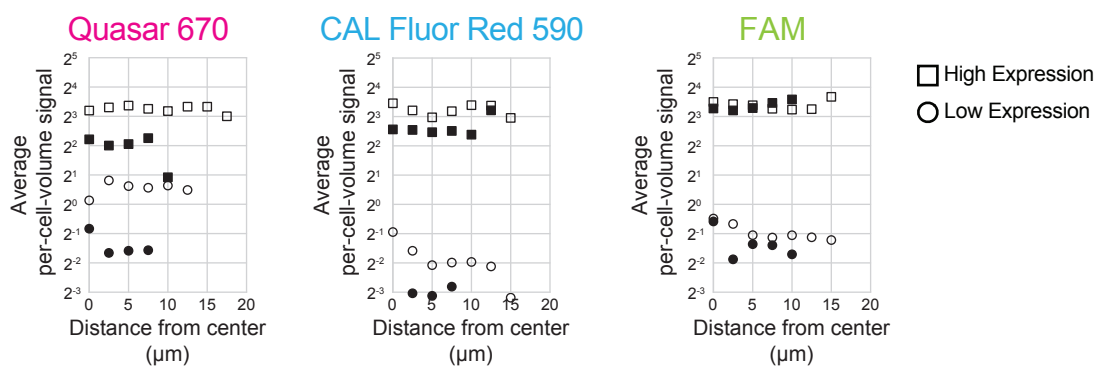

A

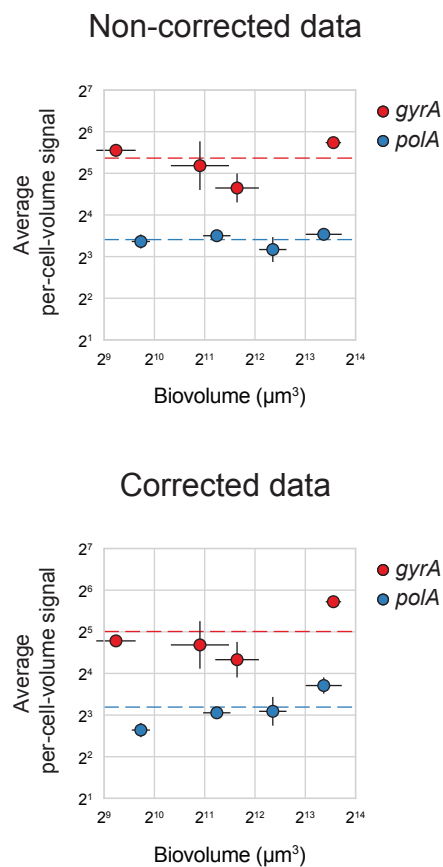

B

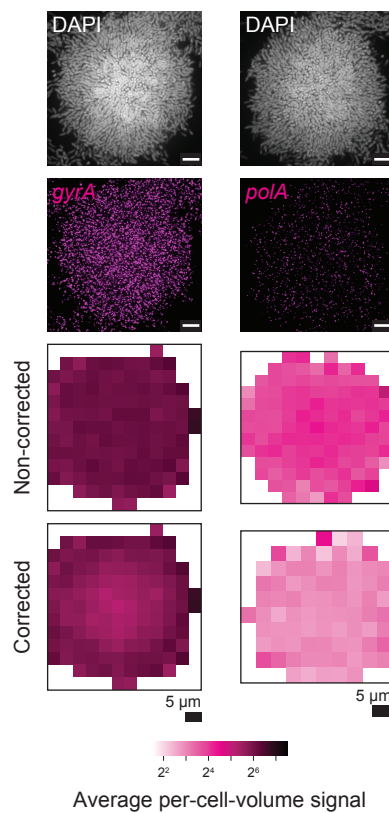

C

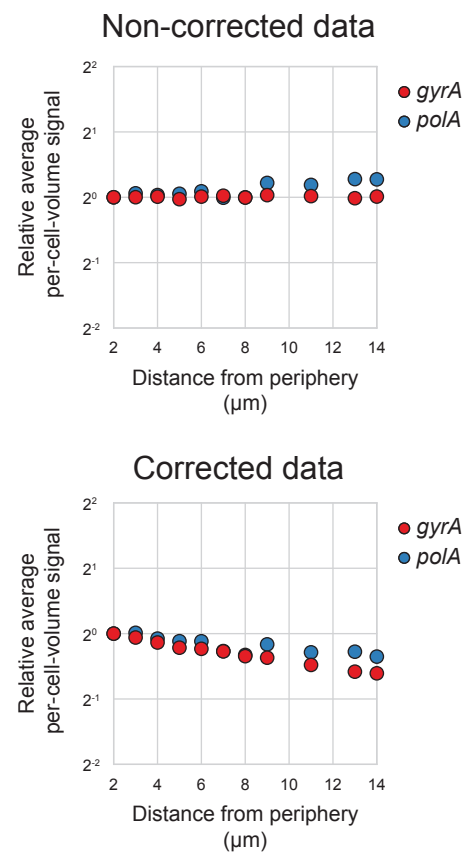

D

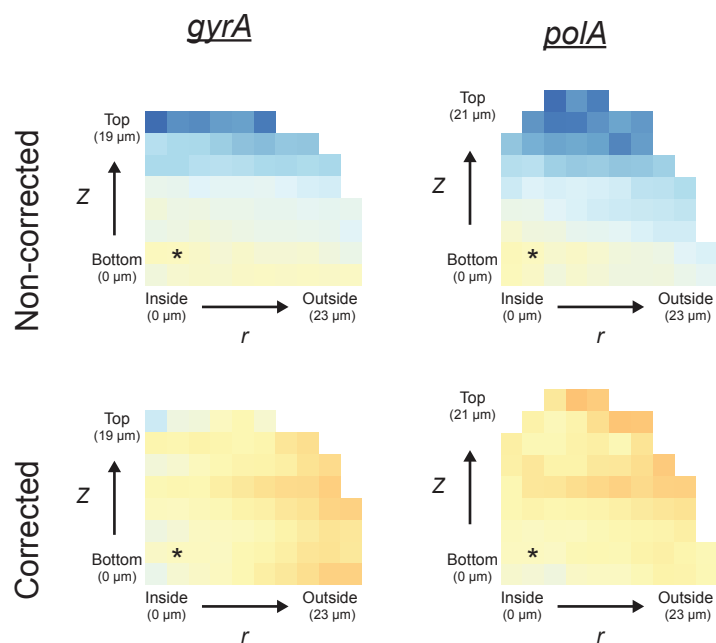

E

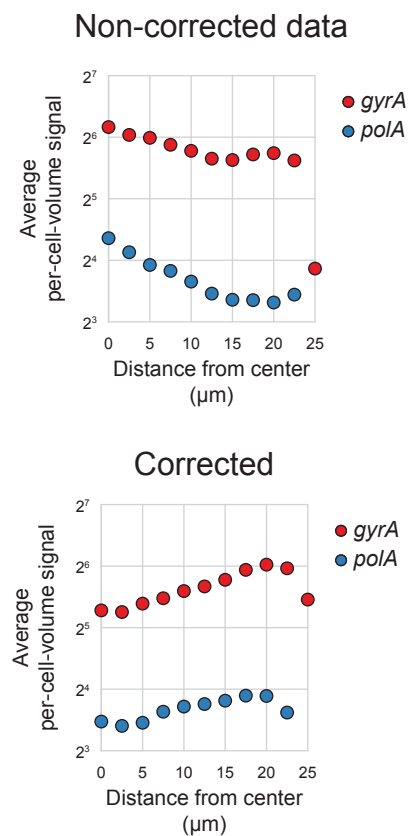

A

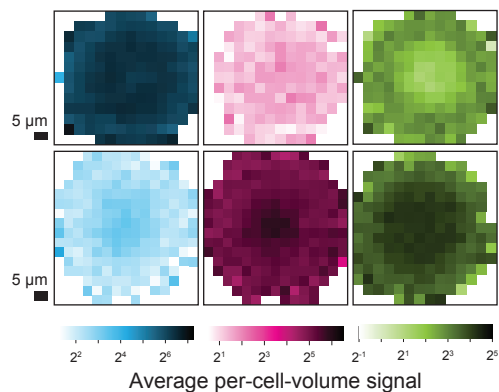

B

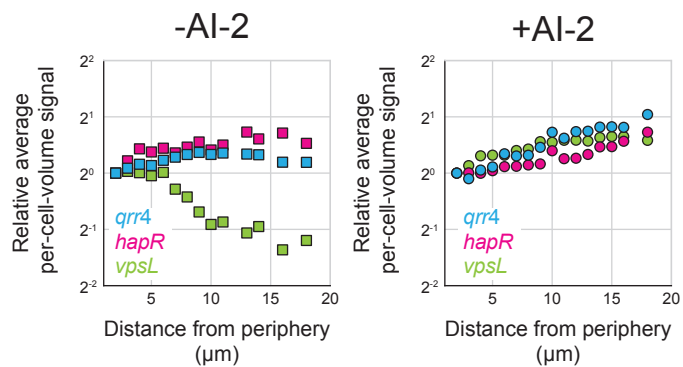

C

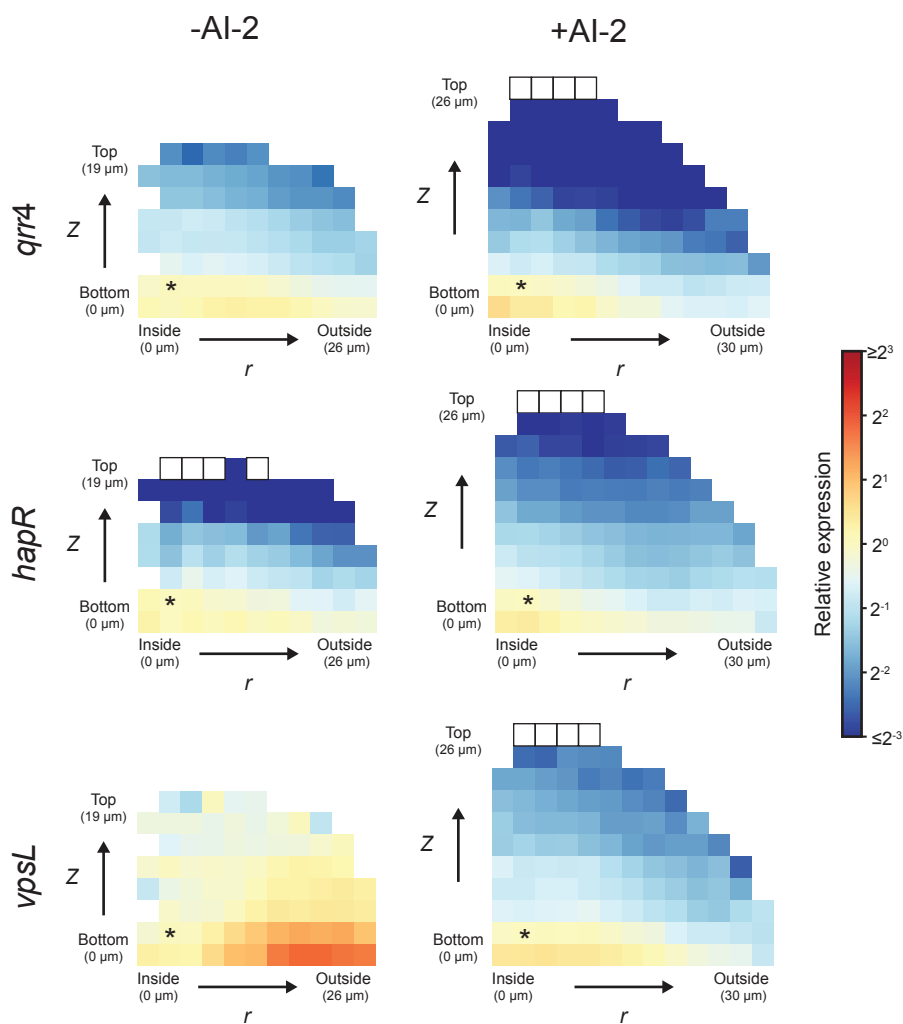

D

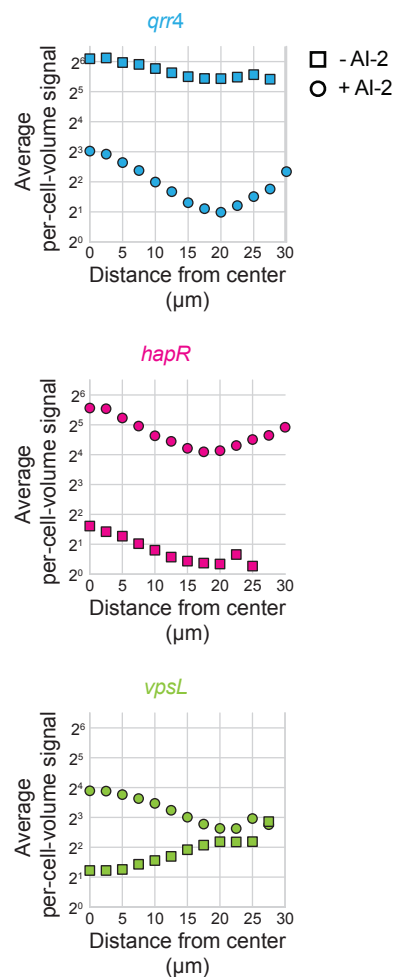

E

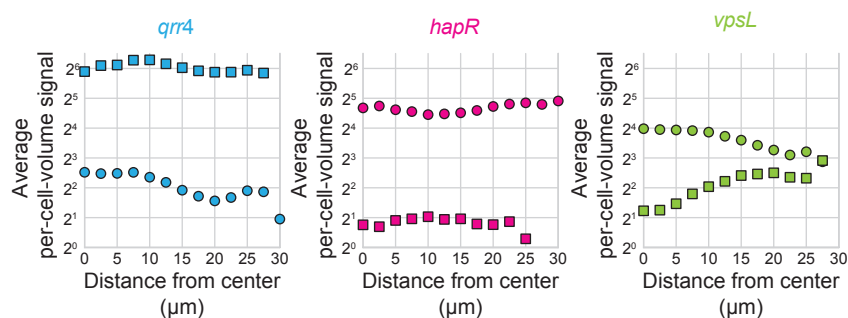

A

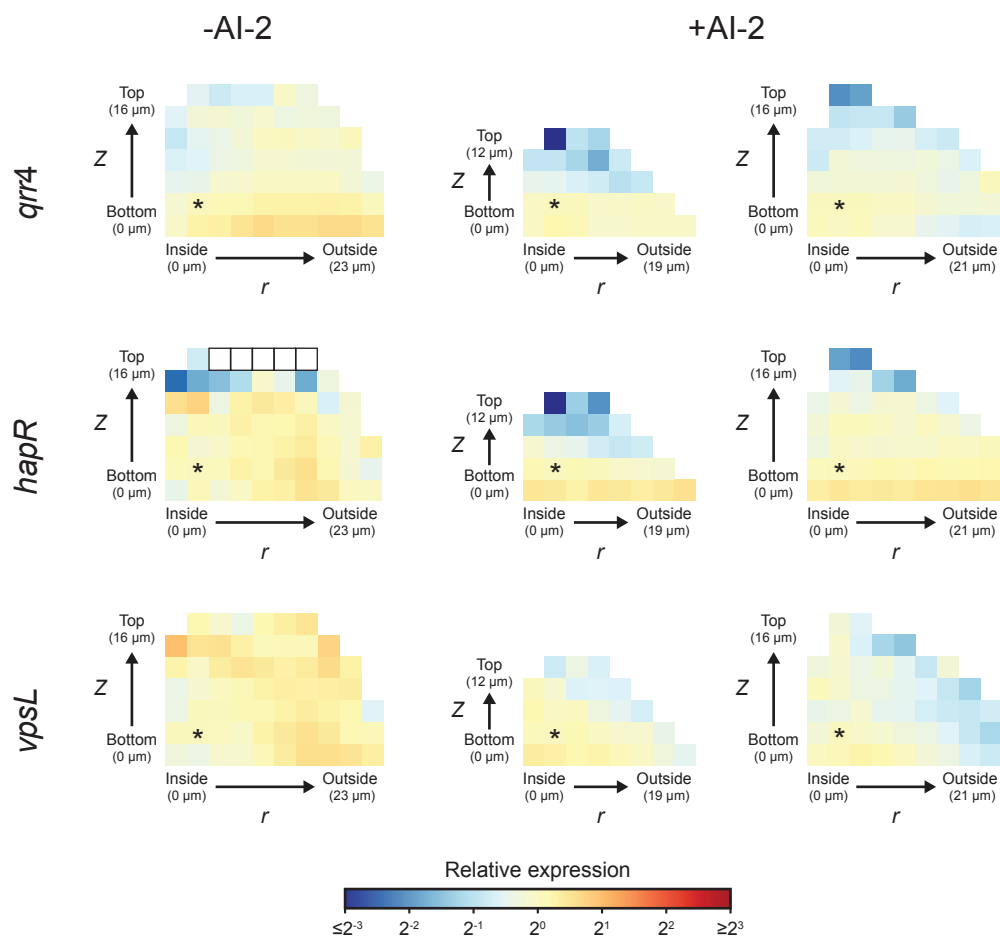

B

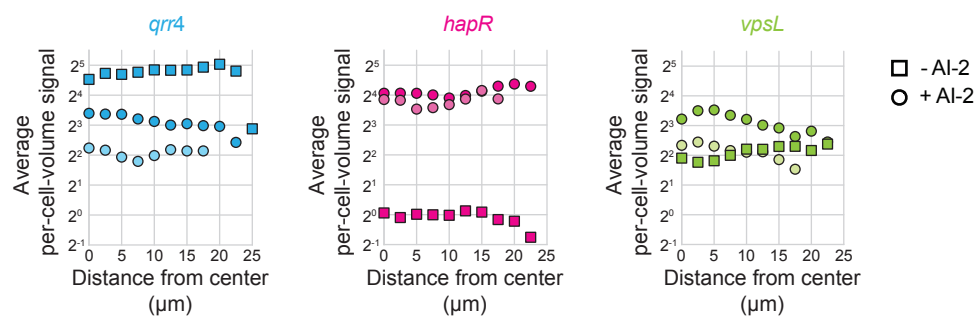

A

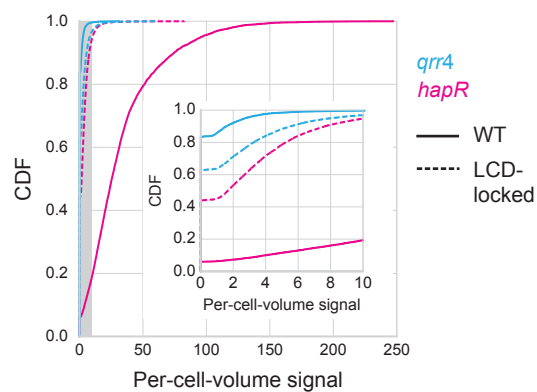

B

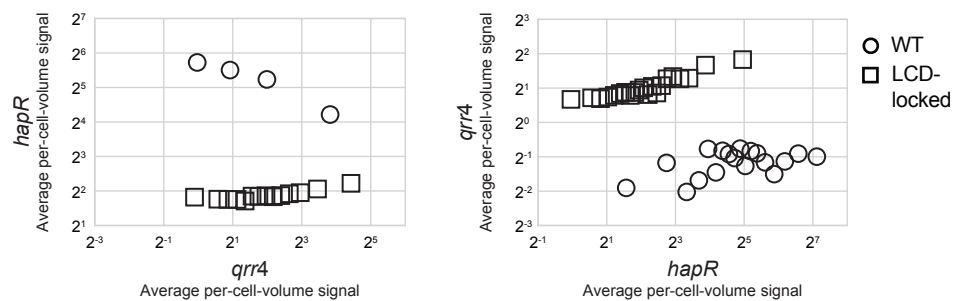

C

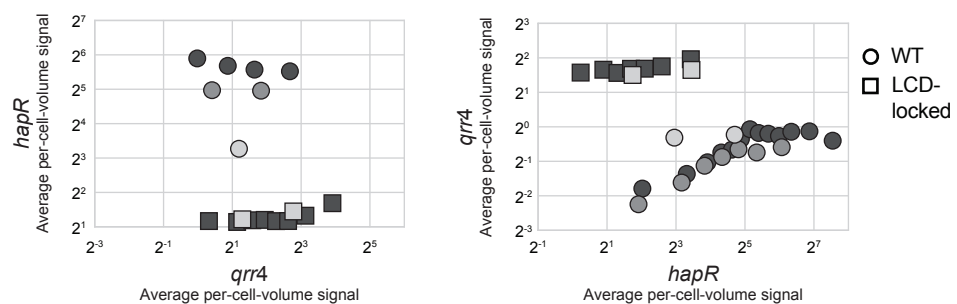

D

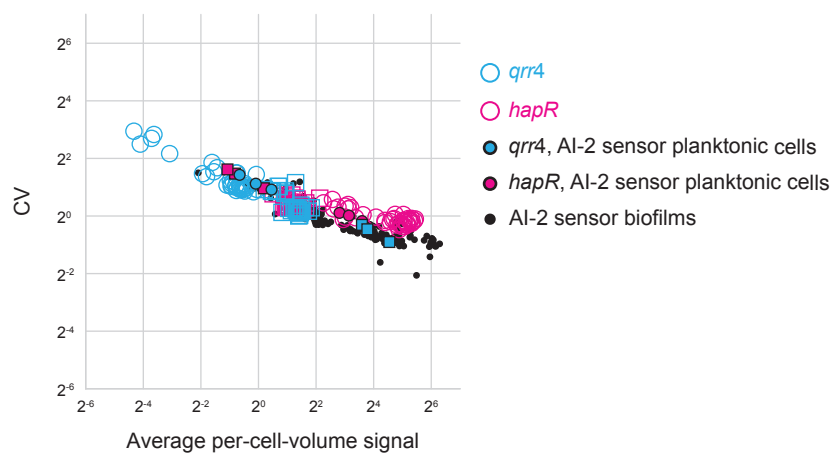

A

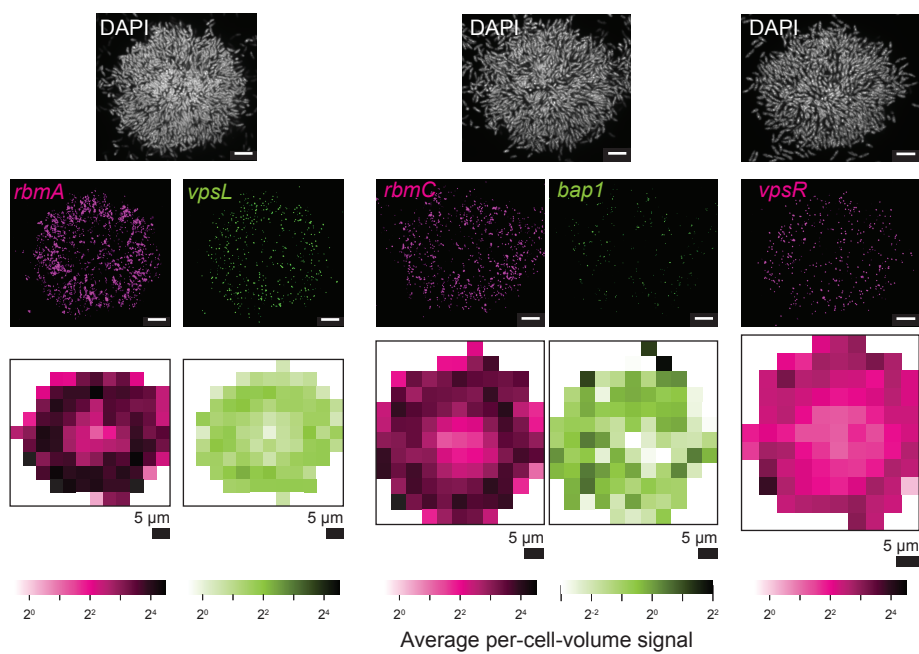

B

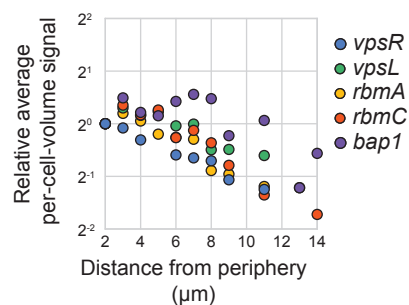

C

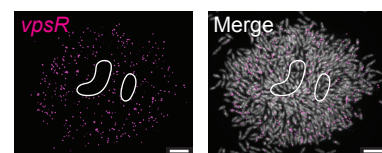

D

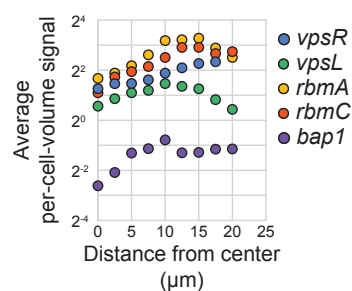

E

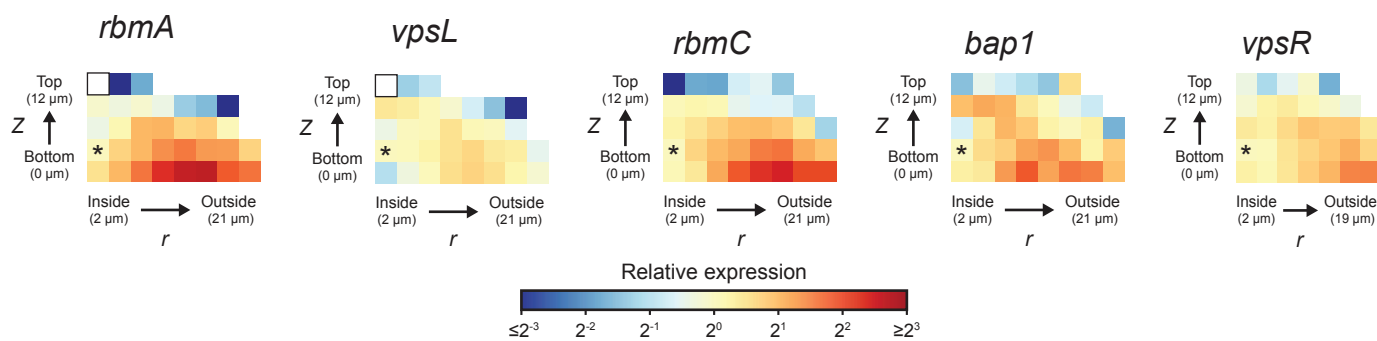

F

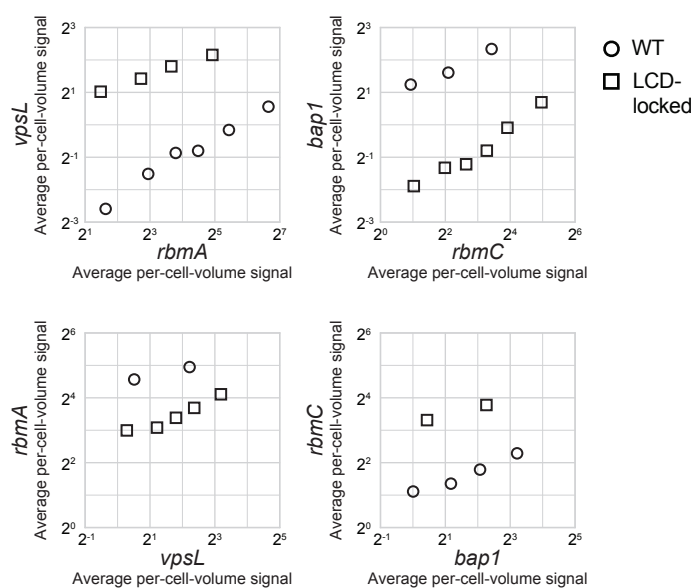

G

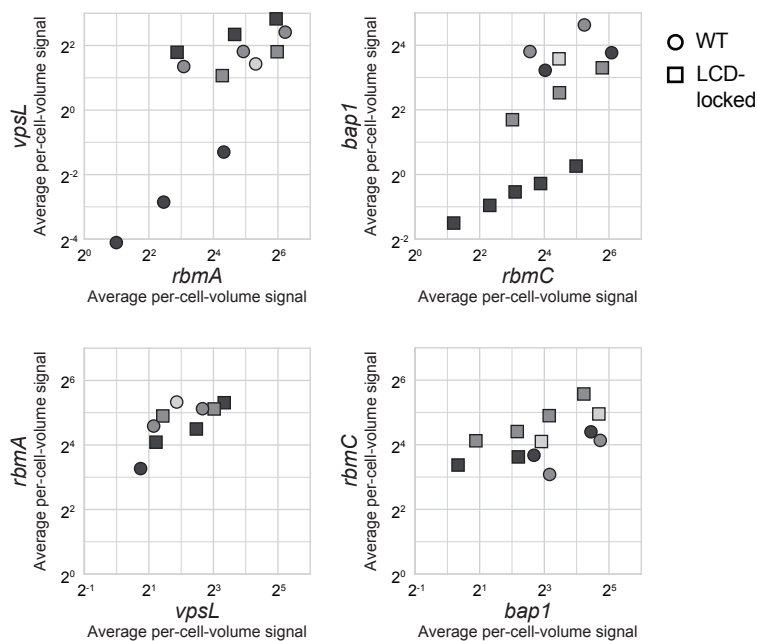

Supplement: Supplement 1 — S1 Fig. Confocal microscopy biases regarding constitutive fluorescent reporters and stains. (A) Maximum projection confocal microscopy images of mScarlet and mNG fluorescence over time in a representative V. cholerae biofilm harboring pTac-mScarlet and pTac-mNG. (B) Signal from DAPI staining in the first in-focus z slice and xz and yz cross sections of a mature V. cholerae biofilm. All scale bars represent 5 μm. S2 Fig. Quantitation of spatial per-cell-volume smFISH fluorescence signal. (A) Average per-cell-volume mNG smFISH fluorescence signal measured in V. cholerae planktonic and biofilm cells. 0.2% or 0.0375% arabinose was provided, as indicated by shaded blue boxes denoted High and Low Expression, respectively. 100 μM Nspd was included in all cases. mNG expression was measured by smFISH using probes labeled with one of three fluorophores, as indicated. Values for biofilm cells represent the mean normalized per-cell-volume fluorescence signal, calculated as described in (B), across n = 10–12 biofilms. The small, medium, and large circular symbols represent biofilms with approximate biovolumes of 29, 211, and 214 μm3, respectively. Error bars denote standard deviations, which are in some cases smaller than the sizes of the symbols used in the plots. Values for planktonic cells represent the average across all cells in single replicate experiments; error bars are excluded. (B) Schematic overview of quantitation of per-cell-volume fluorescence signal. Briefly, using BiofilmQ [43], biofilms are broken into cubes with side lengths of 2.32 μm and cells within these cubes are identified using the DAPI channel. The cell volume (Vc) for each cube is calculated as the fraction of the total cube volume occupied by cell mass. Puncta from smFISH fluorescence signal are detected as local maxima, and the integrated, background-subtracted punctum intensity calculated (I). The per-cell-volume fluorescence signal is subsequently calculated as the ratio of the puncta intensity and [file media-1.pdf]
