# Supplementary material for: Cell-scale gene-expression measurements in Vibrio cholerae biofilms reveal spatiotemporal patterns underlying development
Source: bioRxiv. 2025 Jan 16:2024.07.17.603784. Originally published 2024 Jul 17. Preprint. [Version 2] doi: 10.1101/2024.07.17.603784 (PMC11275835; doi:10.1101/2024.07.17.603784)

S2A, non-corrected data

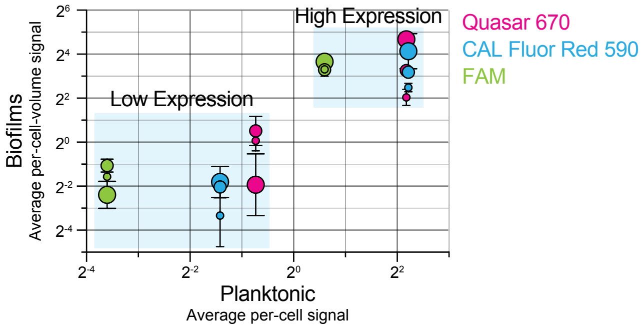

S3B, non-corrected data

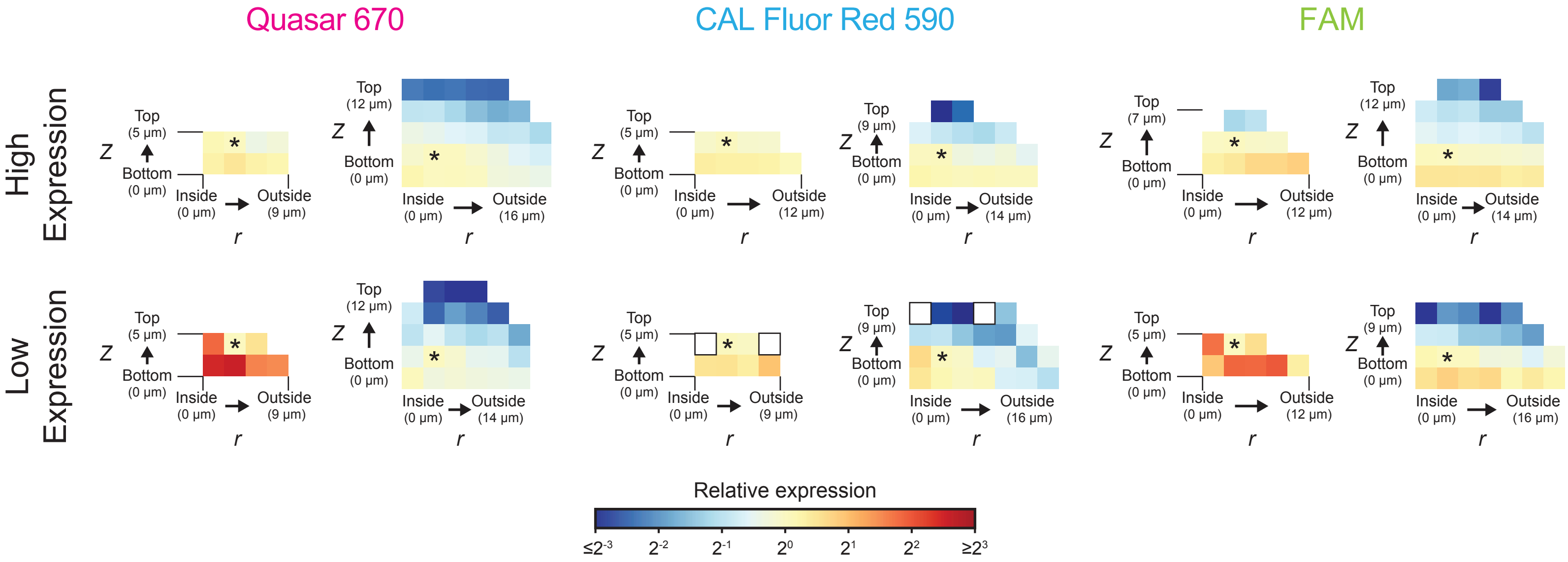

S3C, non-corrected data

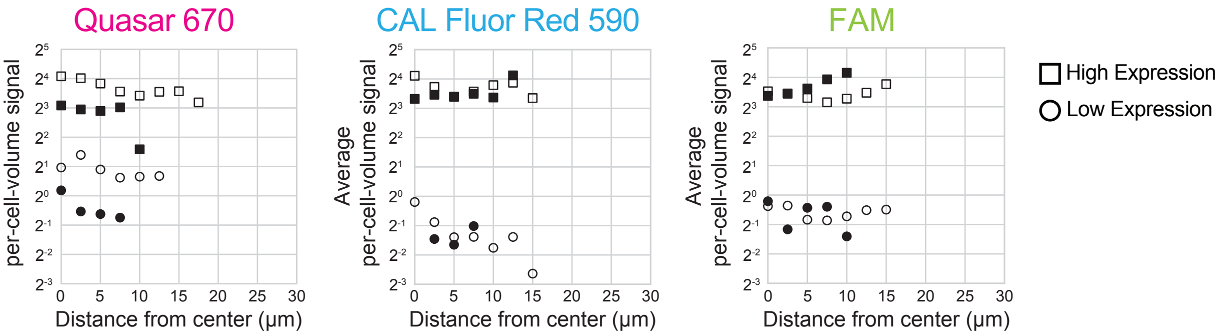

Fig 3A, non-corrected data

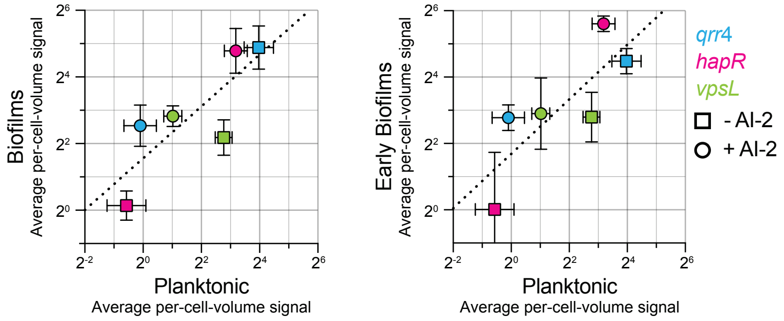

Fig 3B, non-corrected data

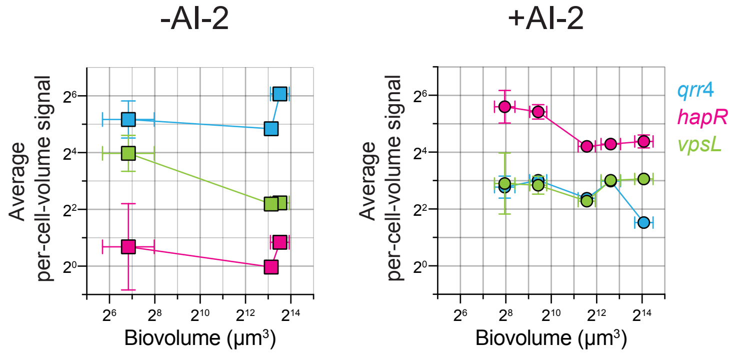

S6A, non-corrected data

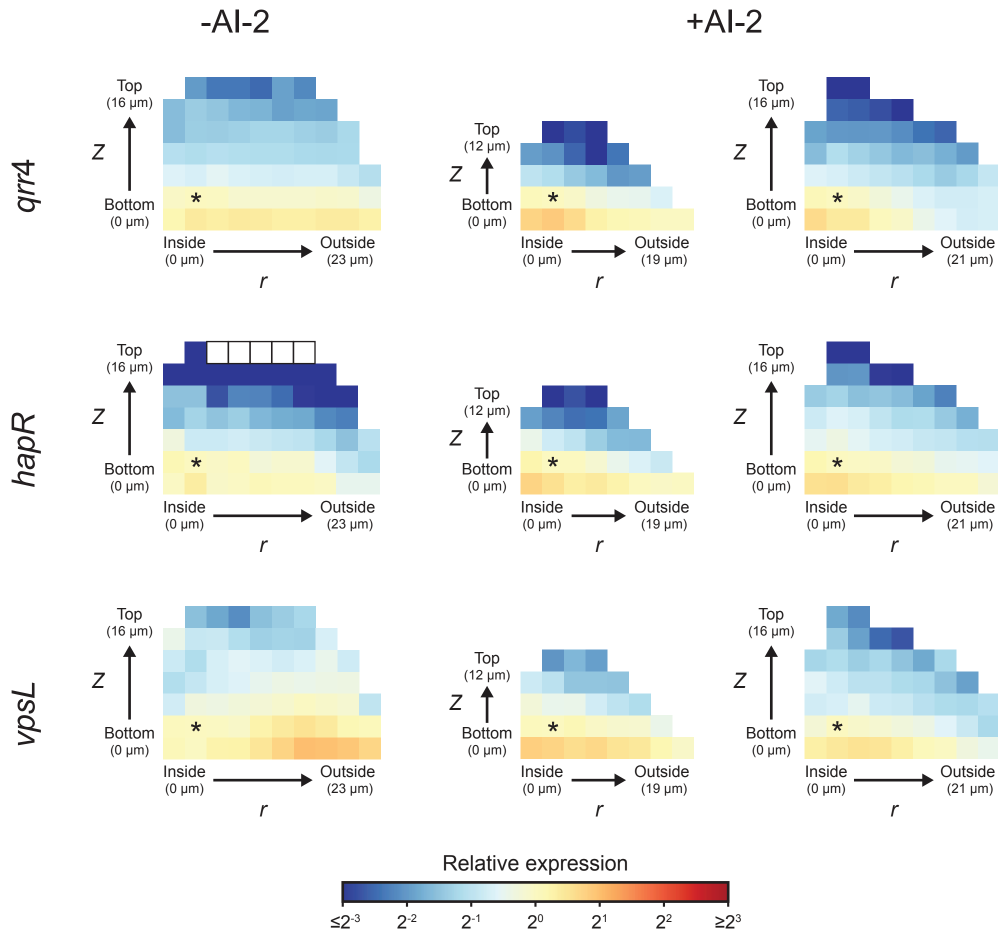

S6B, non-corrected data

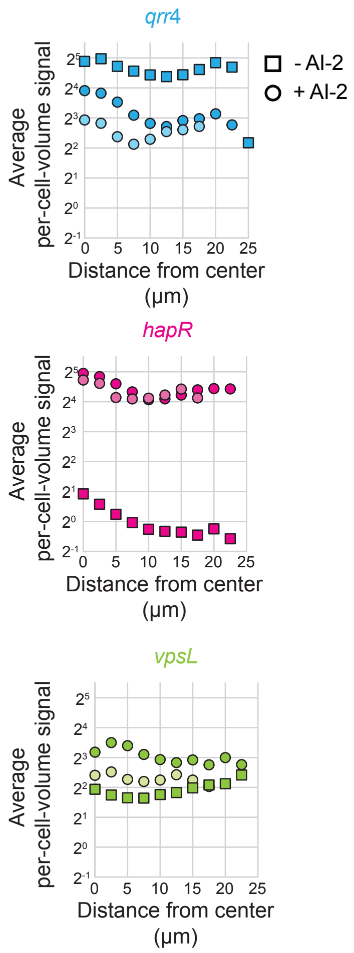

Fig 4A, non-corrected data

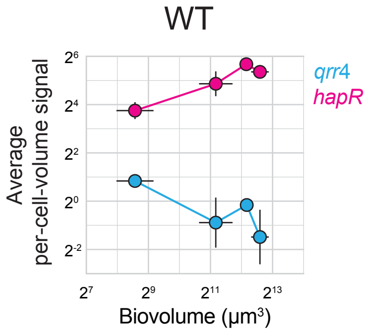

Fig 4B, non-corrected data

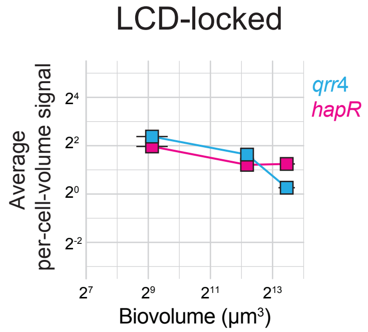

Fig 4C, non-corrected data

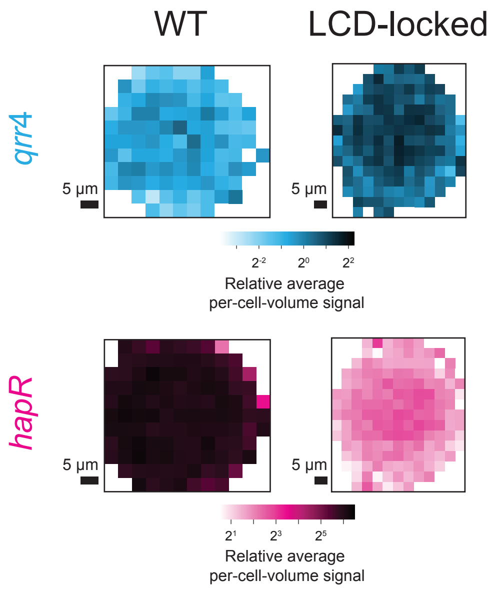

Fig 4D, non-corrected data

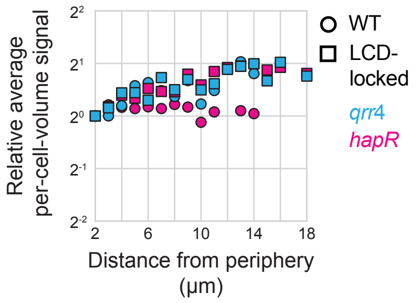

Fig 4E, non-corrected data

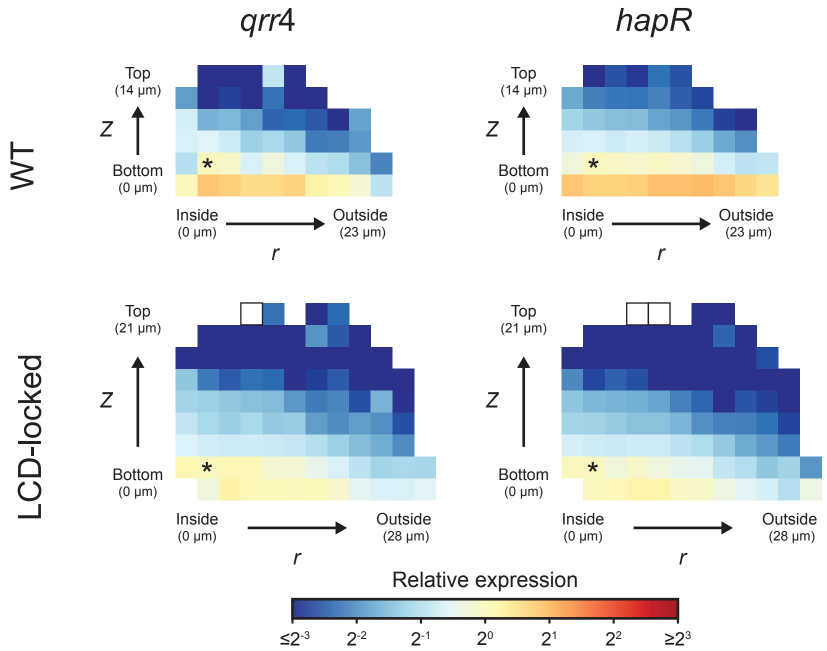

Fig 4F non-corrected data

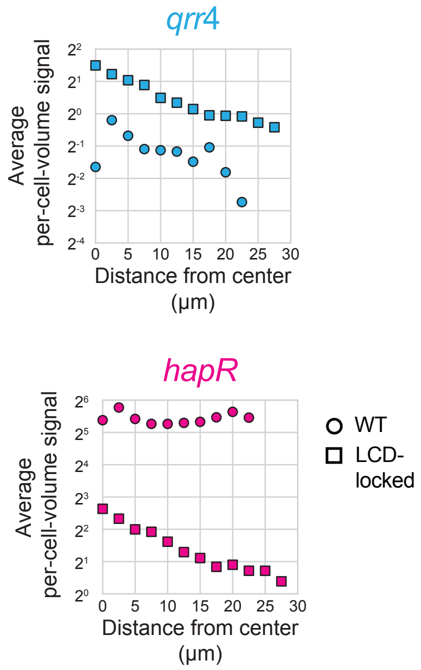

Fig 5A, non-corrected data

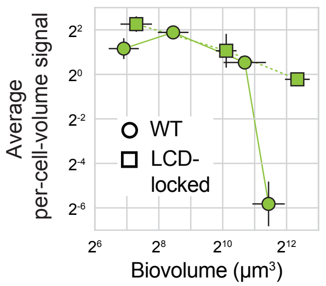

Fig 5B, non-corrected data

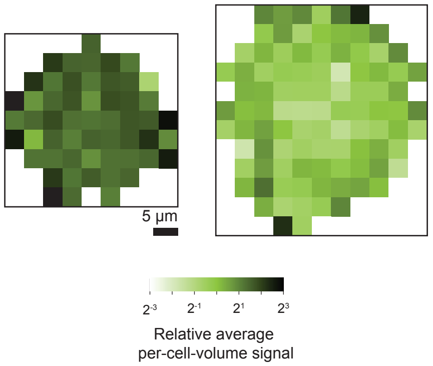

Fig 5C, non-corrected data

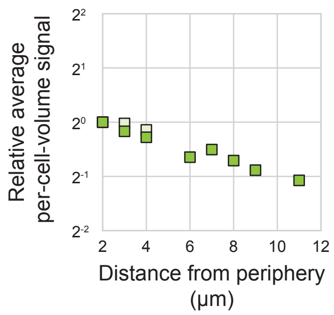

Fig 5D, non-corrected data

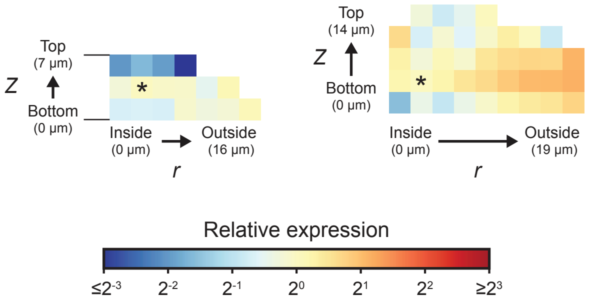

Fig 5E, non-corrected data

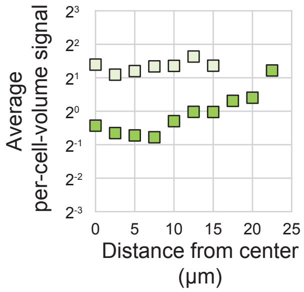

Fig 6A, non-corrected data

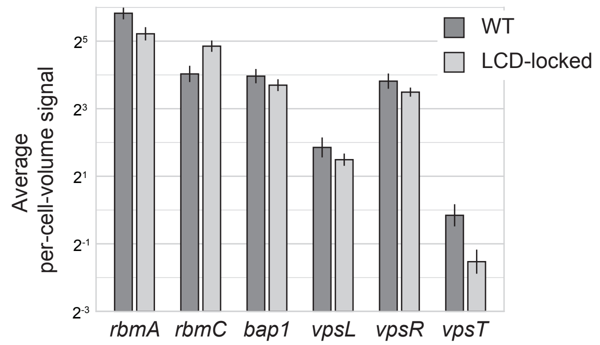

Fig 6B, non-corrected data

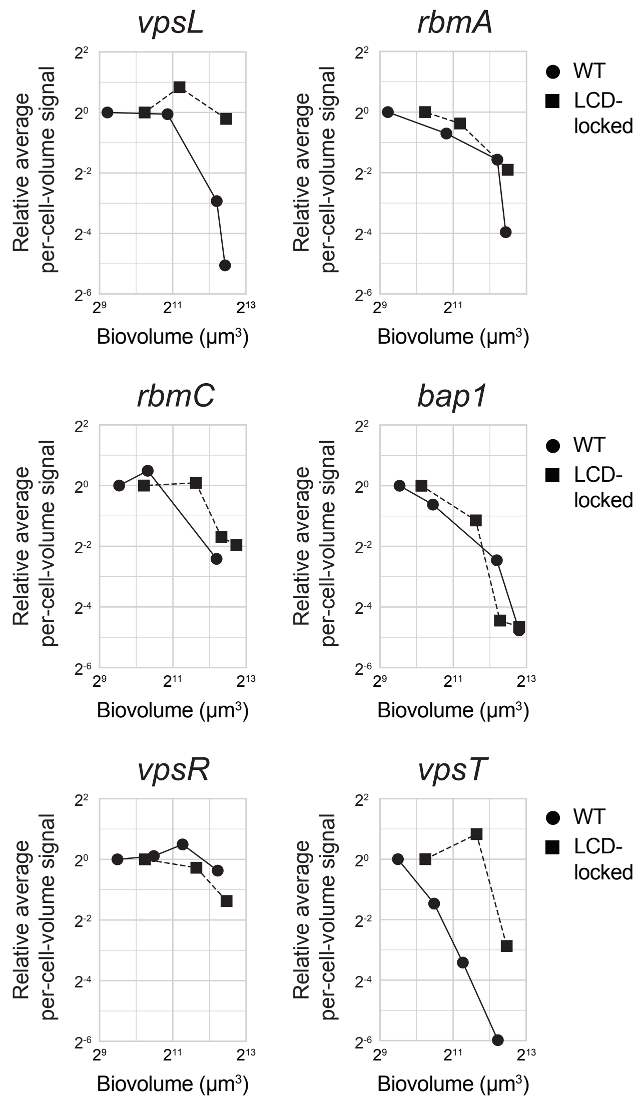

Fig 6C, non-corrected data

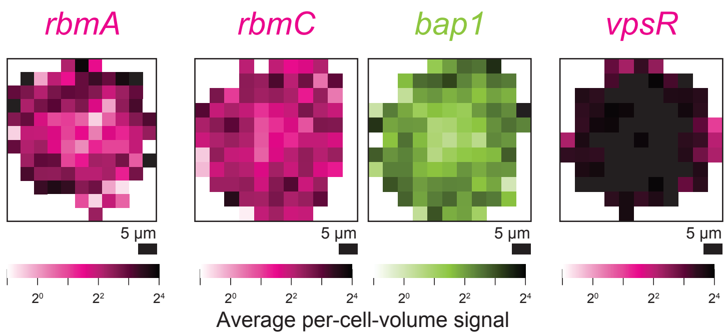

Fig 6D, non-corrected data

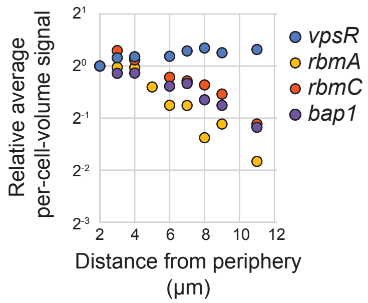

Fig 6E, non-corrected data

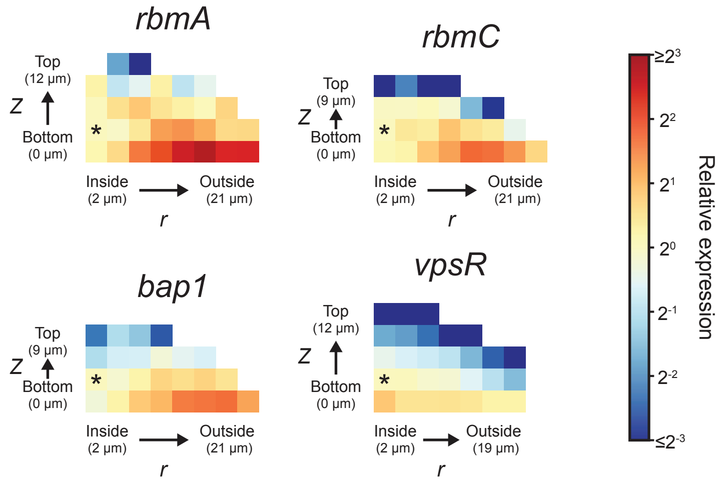

Fig 6F, non-corrected data

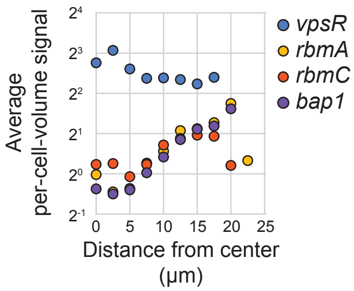

S8A, non-corrected data

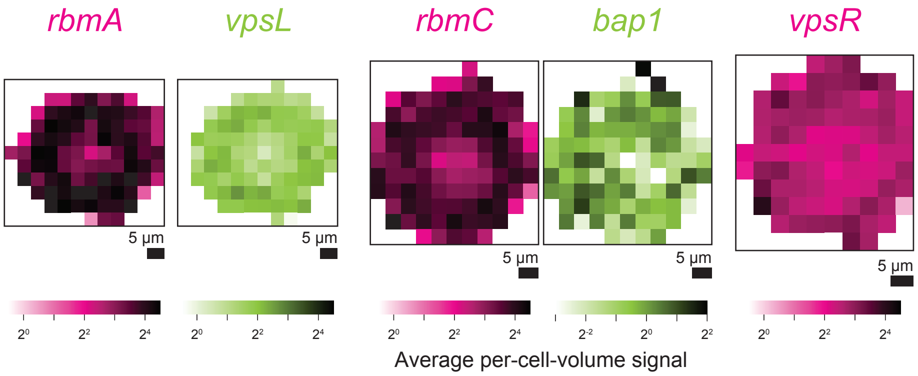

S8B, non-corrected data

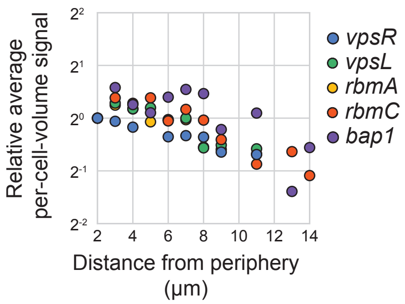

S8D, non-corrected data

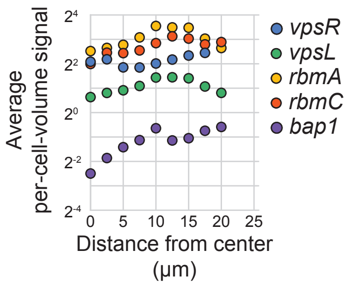

S8E, non-corrected data

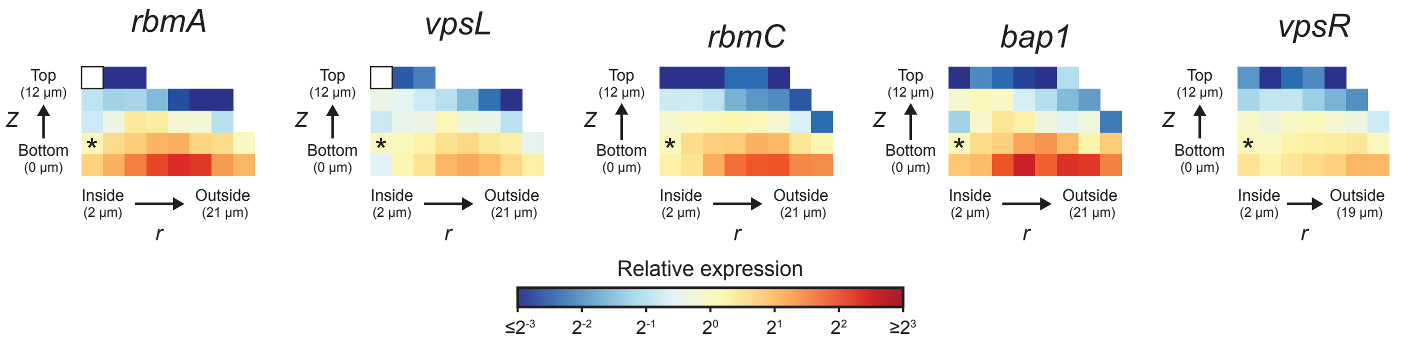

Supplement: Supplement 2 — S1 Data. Companion plots generated from non-corrected data. For each plot, the corresponding main text or supplemental figure is indicated. [file media-2.pdf]
