# Supplementary material for: Single-cell gene-expression measurements in Vibrio cholerae biofilms reveal spatiotemporal patterns underlying development
Source: bioRxiv. 2024 Jul 17:2024.07.17.603784. Preprint. [Version 1] doi: 10.1101/2024.07.17.603784 (PMC11275835; doi:10.1101/2024.07.17.603784)
Supplement: Supplementary file 1 [file NIHPP2024.07.17.603784v1-supplement-4.pdf]

Fig. S1

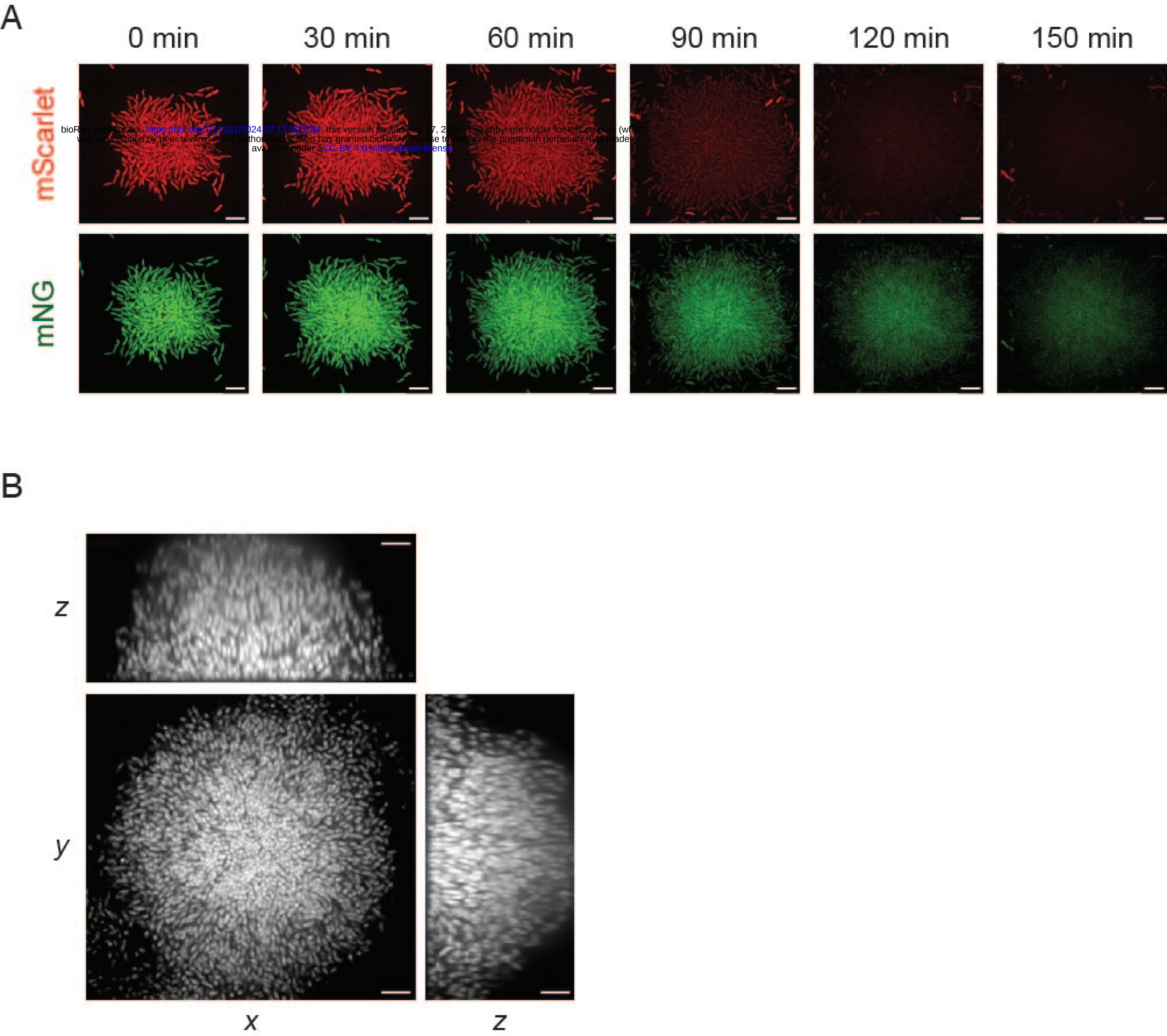

bioRxiv preprint doi: https://doi.org/10.1101/2024.07.17.603784; this version posted July 17, 2024. The copyright holder for this preprint (which was not certified by peer review) is the author/funder, who has granted bioRxiv a license to display the preprint in perpetuity. It is made available under aCC-BY 4.0 International license.

Fig. S2

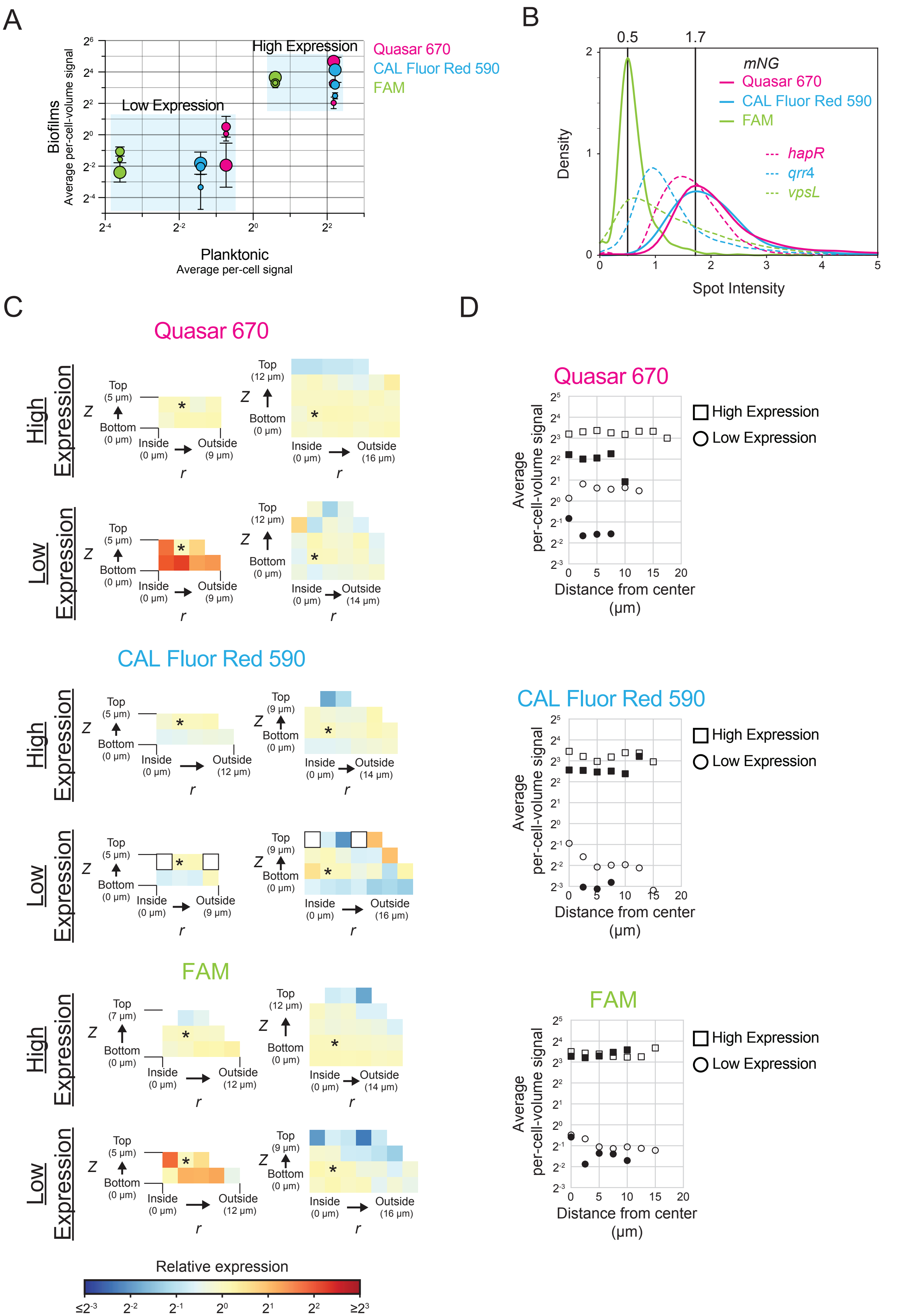

A
Biofilms
Average per-cell-volume signal
Planktonic
Average per-cell signal
High Expression
Low Expression
Quasar 670
CAL Fluor Red 590
FAM
B
0.5
1.7
Density
Spot Intensity
mNG
Quasar 670
CAL Fluor Red 590
FAM
hapR
qrr4
vpsL
C
Quasar 670
CAL Fluor Red 590
FAM
High Expression
Low Expression
Top
Bottom
Inside
Outside
Relative expression
D
Average per-cell-volume signal
Distance from center (μm)
High Expression
Low Expression

Fig. S3

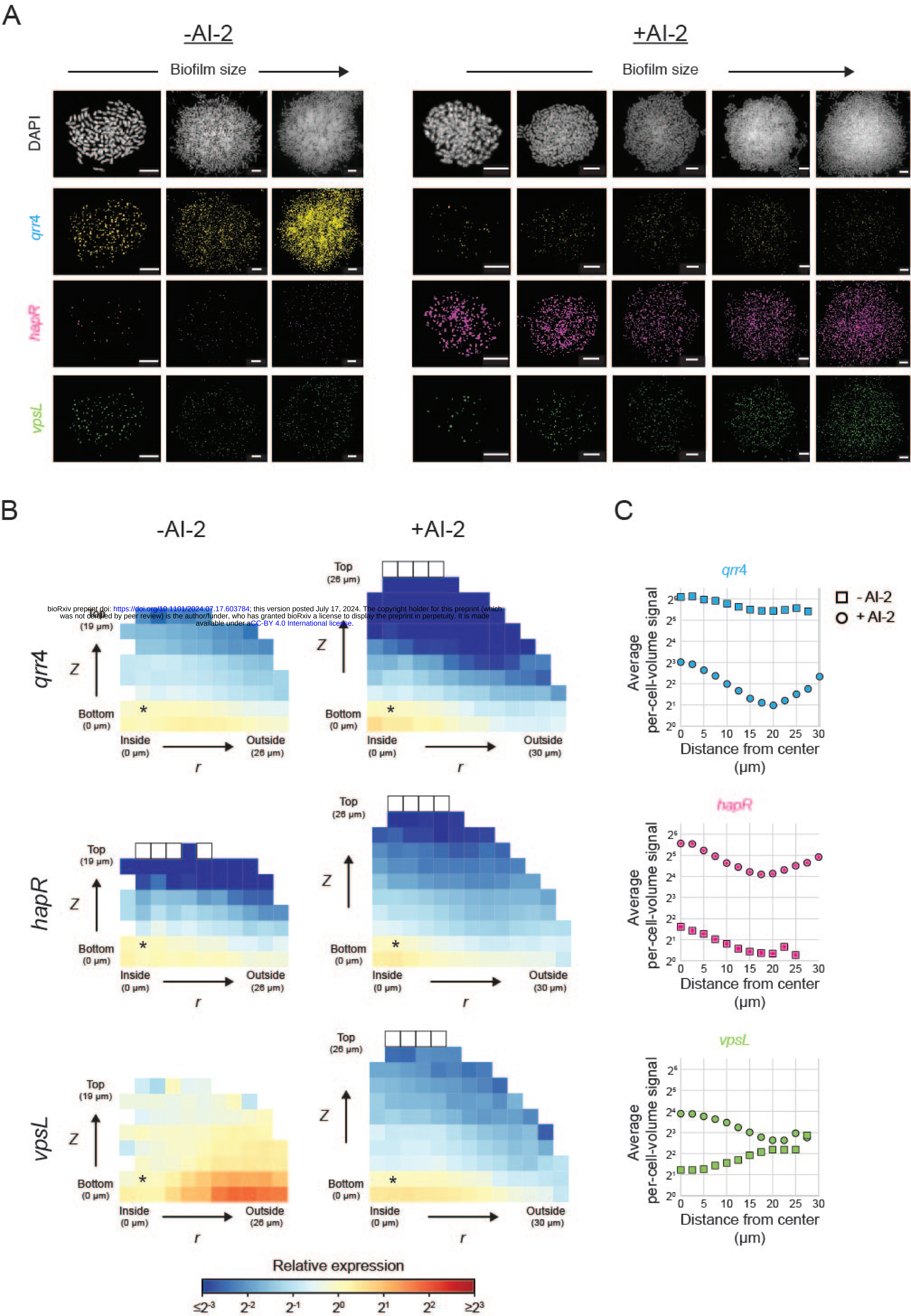

bioRxiv preprint doi: https://doi.org/10.1101/2024.07.17.603784; this version posted July 17, 2024. The copyright holder for this preprint (which was not certified by peer review) is the author/funder, who has granted bioRxiv a license to display the preprint in perpetuity. It is made available under aCC-BY 4.0 International license.

Fig. S4

A

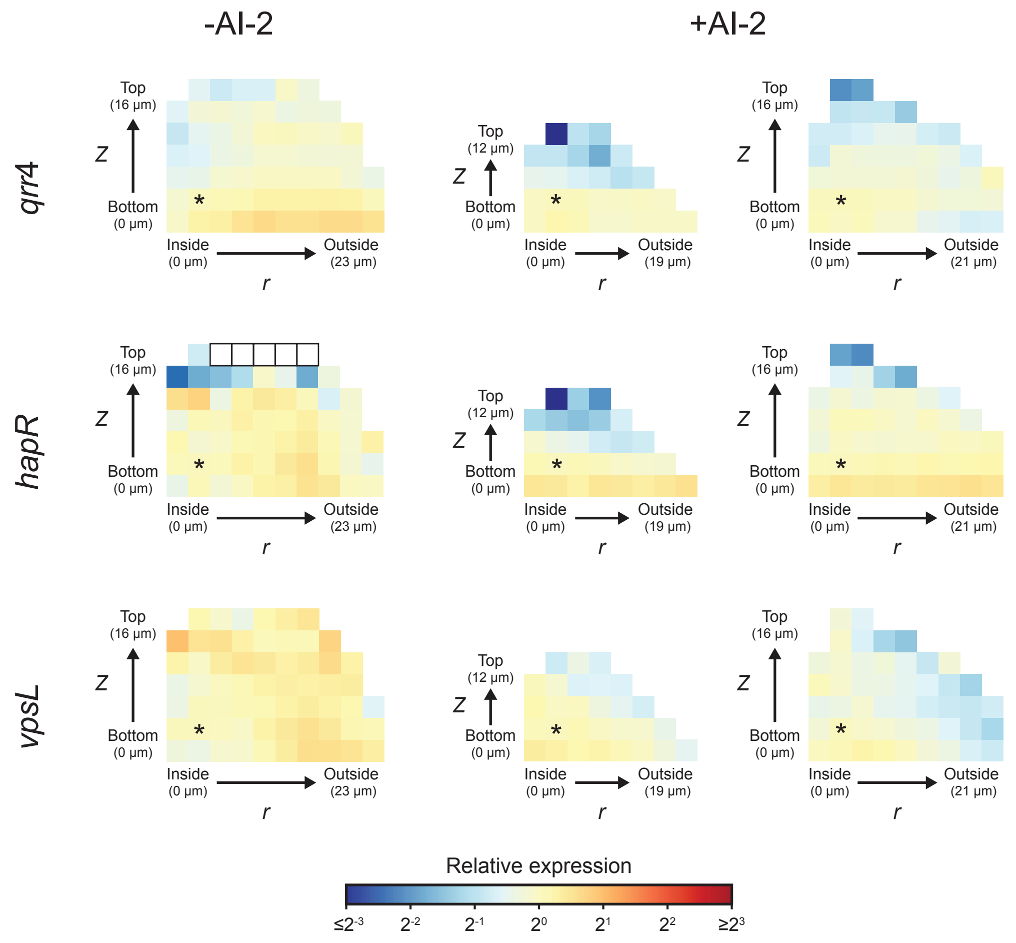
-AI-2
+AI-2
qrr4
hapR
vpsL
Top
(16 μm)
(12 μm)
Z
Bottom
(0 μm)
Inside
(0 μm)
Outside
(23 μm)
(19 μm)
(21 μm)
r
*
Relative expression
≤2^-3
2^-2
2^-1
2^0
2^1
2^2
≥2^3

B

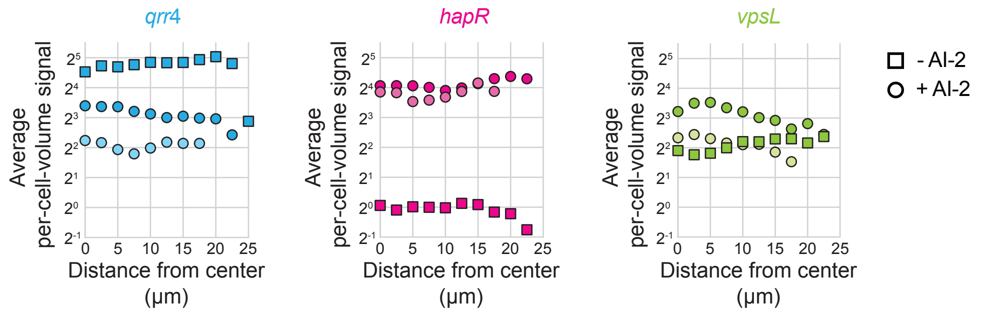
qrr4
hapR
vpsL
Average per-cell-volume signal
Distance from center (μm)
0 5 10 15 20 25
- AI-2
+ AI-2

Fig. S5

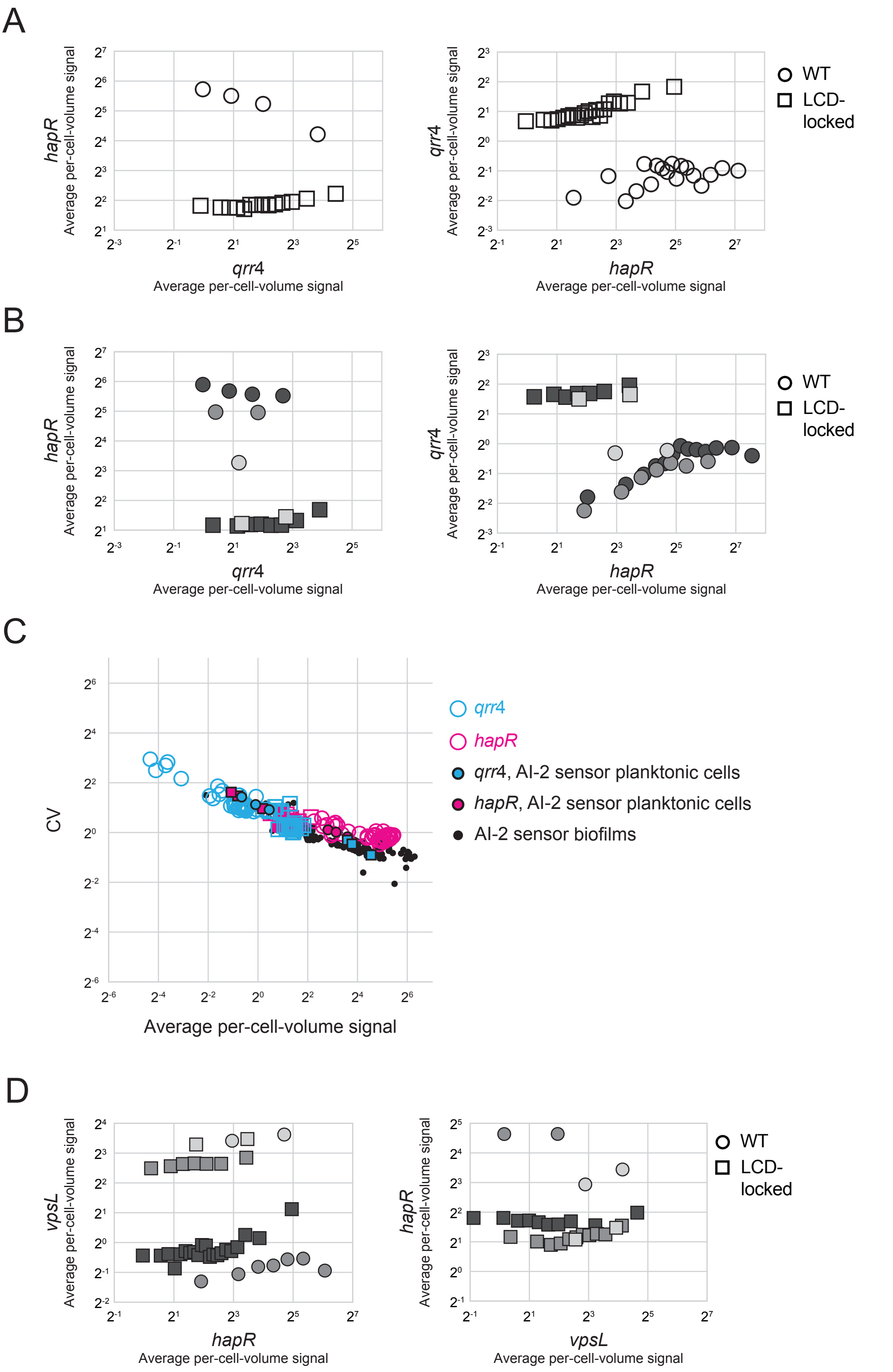
A
hapR
Average per-cell-volume signal
qrr4
Average per-cell-volume signal
qrr4
Average per-cell-volume signal
hapR
Average per-cell-volume signal
WT
LCD-locked
B
hapR
Average per-cell-volume signal
qrr4
Average per-cell-volume signal
qrr4
Average per-cell-volume signal
hapR
Average per-cell-volume signal
WT
LCD-locked
C
CV
Average per-cell-volume signal
qrr4
hapR
qrr4, AI-2 sensor planktonic cells
hapR, AI-2 sensor planktonic cells
AI-2 sensor biofilms
D
vpsL
Average per-cell-volume signal
hapR
Average per-cell-volume signal
hapR
Average per-cell-volume signal
vpsL
Average per-cell-volume signal
WT
LCD-locked

Fig. S6

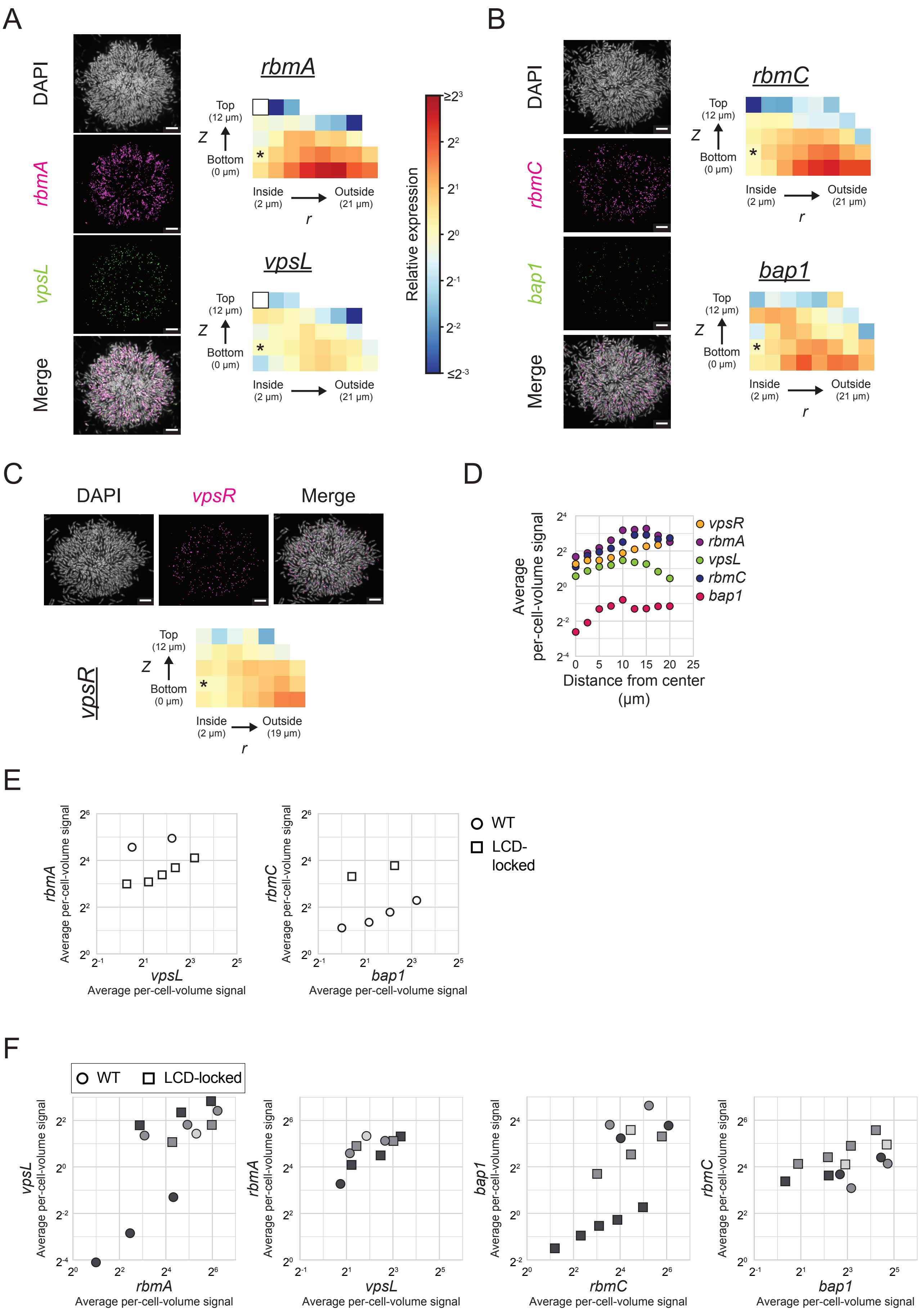

A
DAPI
rbmA
vpsL
Merge
rbmA
Top (12 µm)
Z
Bottom (0 µm)
Inside (2 µm)
Outside (21 µm)
r
vpsL
Relative expression
≥2³
≤2⁻³
B
rbmC
bap1
C
vpsR
Outside (19 µm)
D
Average per-cell-volume signal
Distance from center (µm)
E
WT
LCD-locked
Average per-cell-volume signal
F
