# Supplementary material for: Single-cell gene-expression measurements in Vibrio cholerae biofilms reveal spatiotemporal patterns underlying development
Source: bioRxiv. 2024 Jul 17:2024.07.17.603784. Preprint. [Version 1] doi: 10.1101/2024.07.17.603784 (PMC11275835; doi:10.1101/2024.07.17.603784)

Supplemental Data S1. Companion plots generated from non-corrected data. For each plot, the corresponding main text or supplemental figure is indicated.

Fig. S2A, non-corrected data

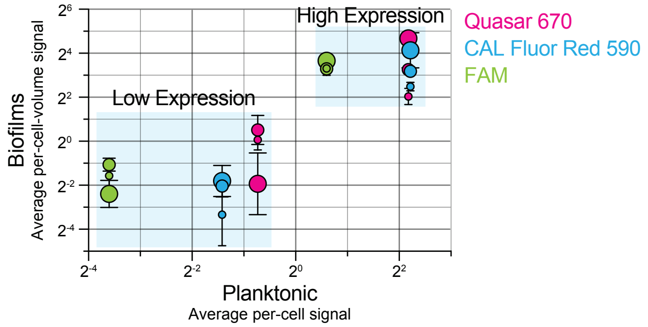

Fig. S2C, non-corrected data

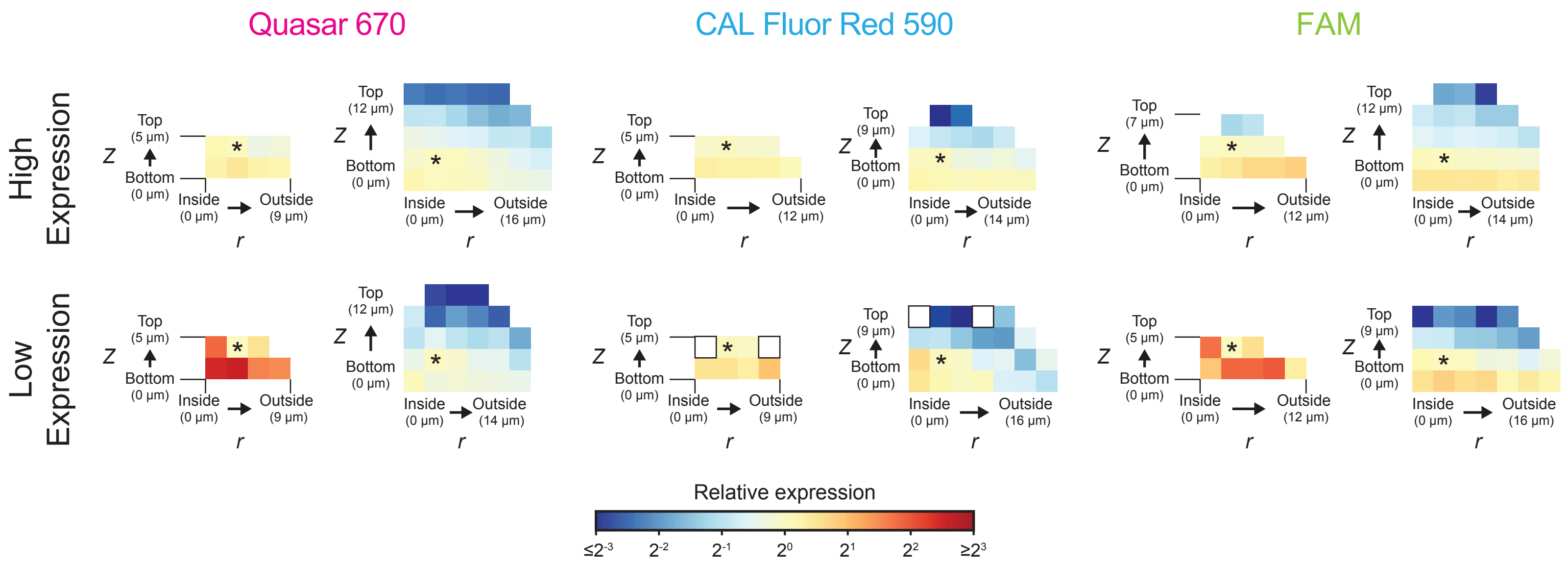

Fig. S2D, non-corrected data

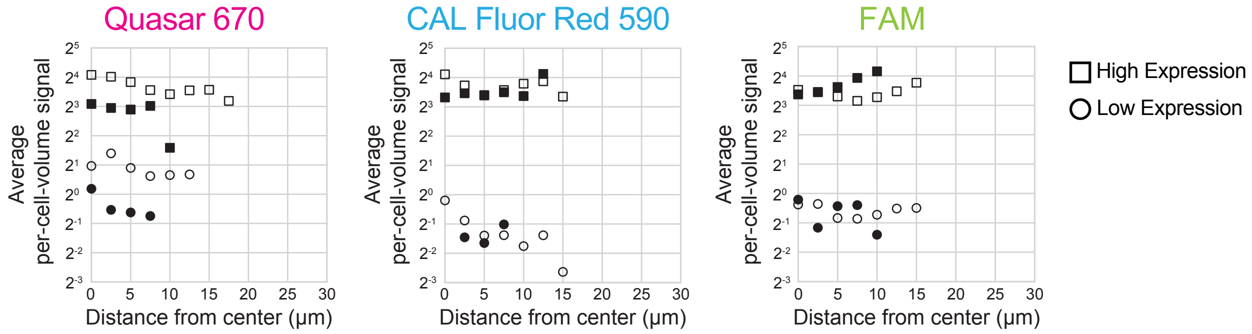

Fig. 3A, non-corrected data

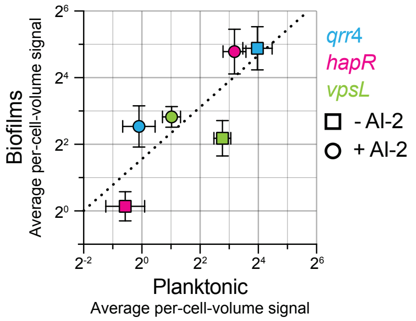

Fig. 3B, non-corrected data

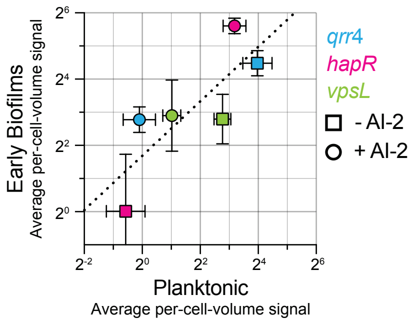

Fig. 3C, non-corrected data

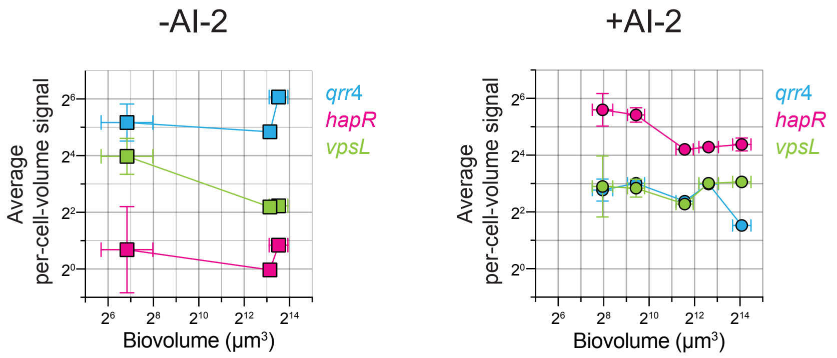

Fig. S4A, non-corrected data

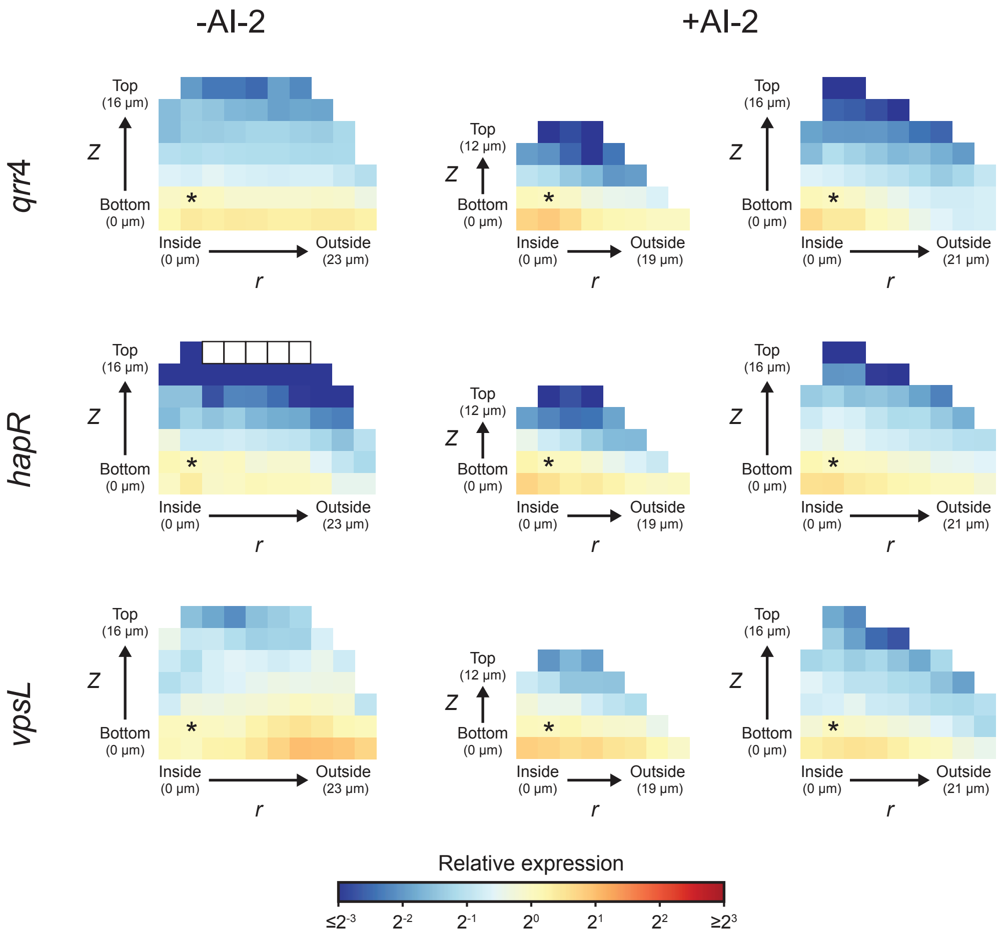

Fig. S4B, non-corrected data

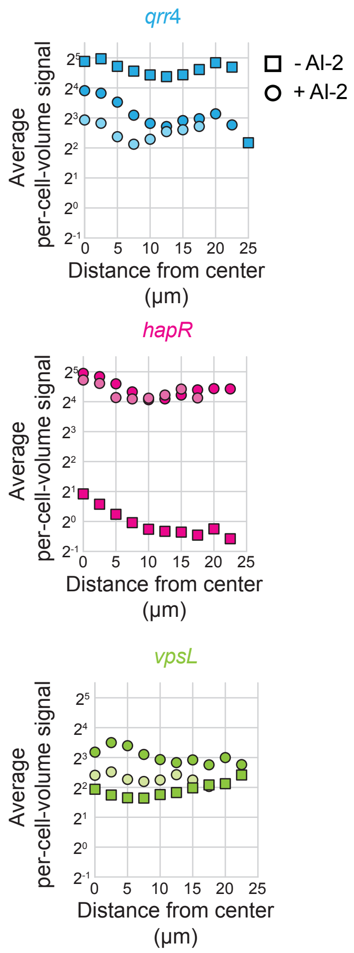

Fig. 4A, non-corrected data

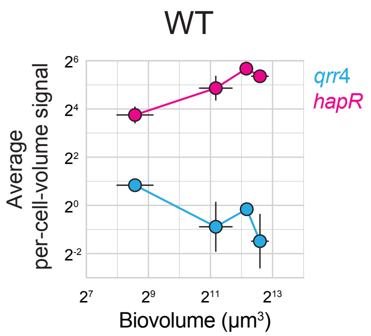

Fig. 4B, non-corrected data

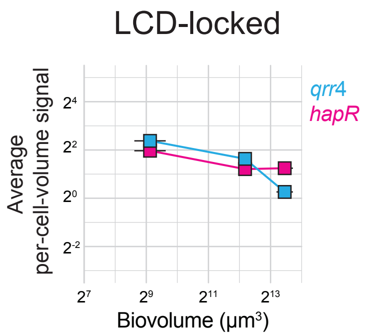

Fig. 4C, non-corrected data

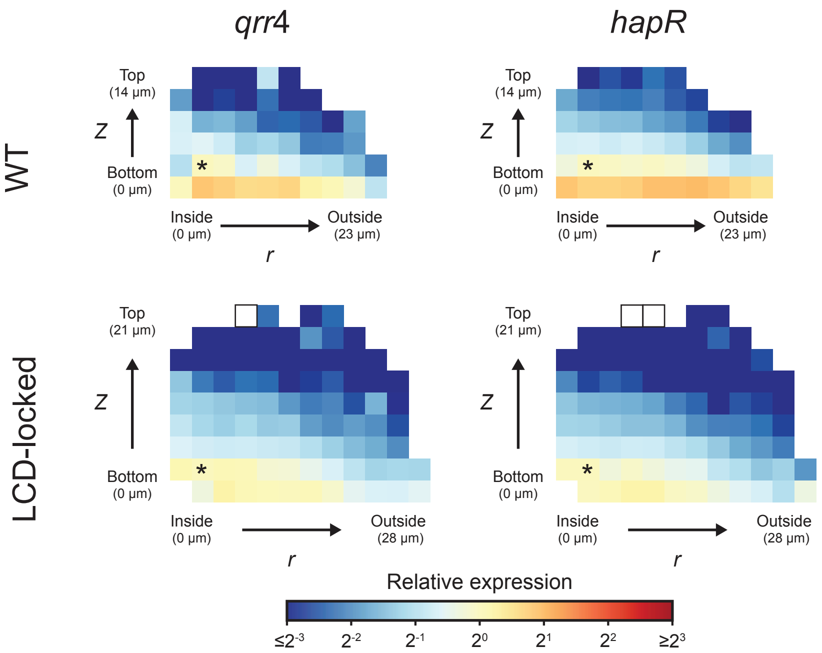

Fig. 4D, non-corrected data

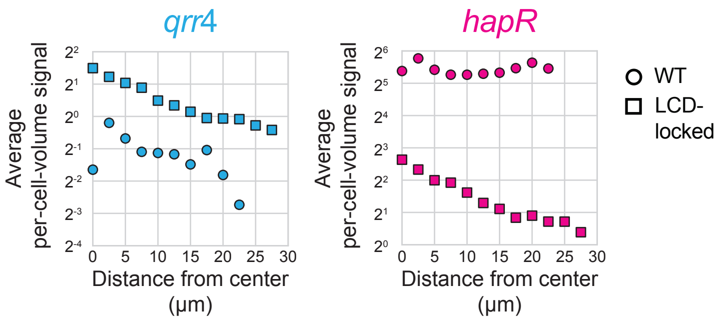

Fig. 5A, non-corrected data

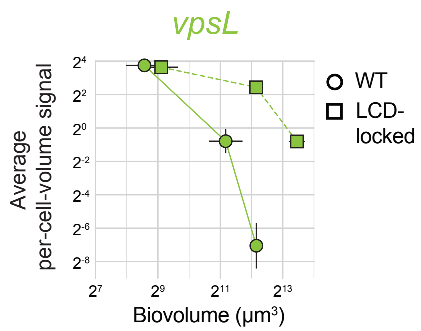

Fig. 5C, non-corrected data

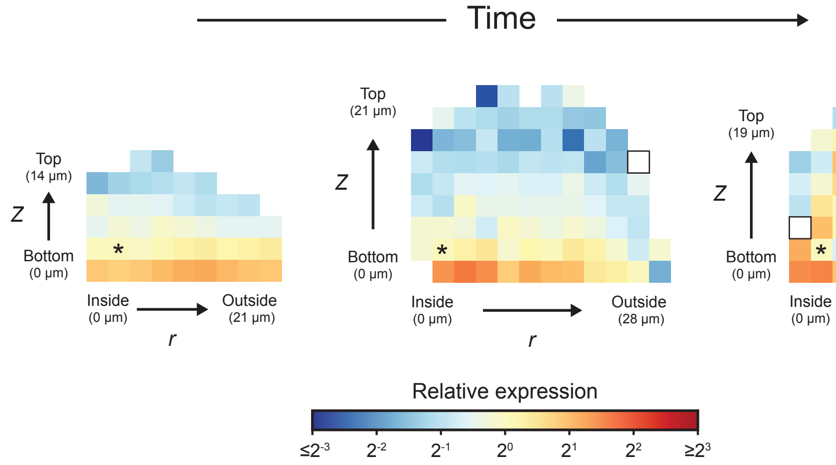

Fig. 5D, non-corrected data

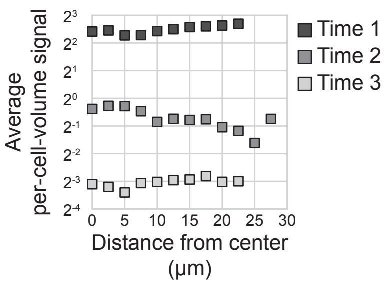

Fig. 5E, non-corrected data

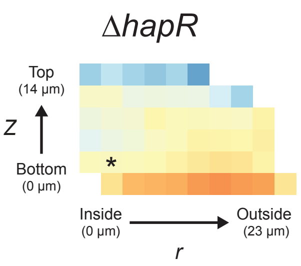

Fig. 5F, non-corrected data

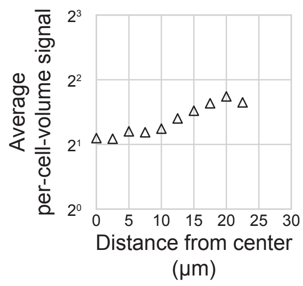

Fig. 6A, non-corrected data

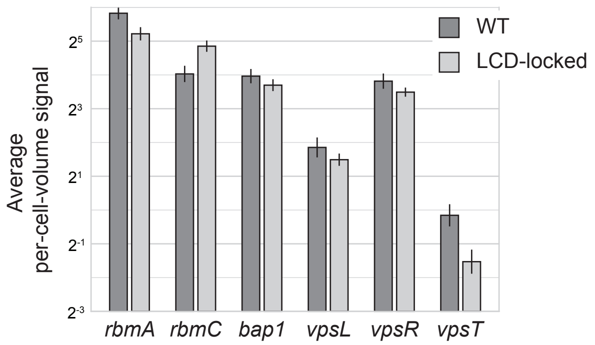

Fig. 6B, non-corrected data

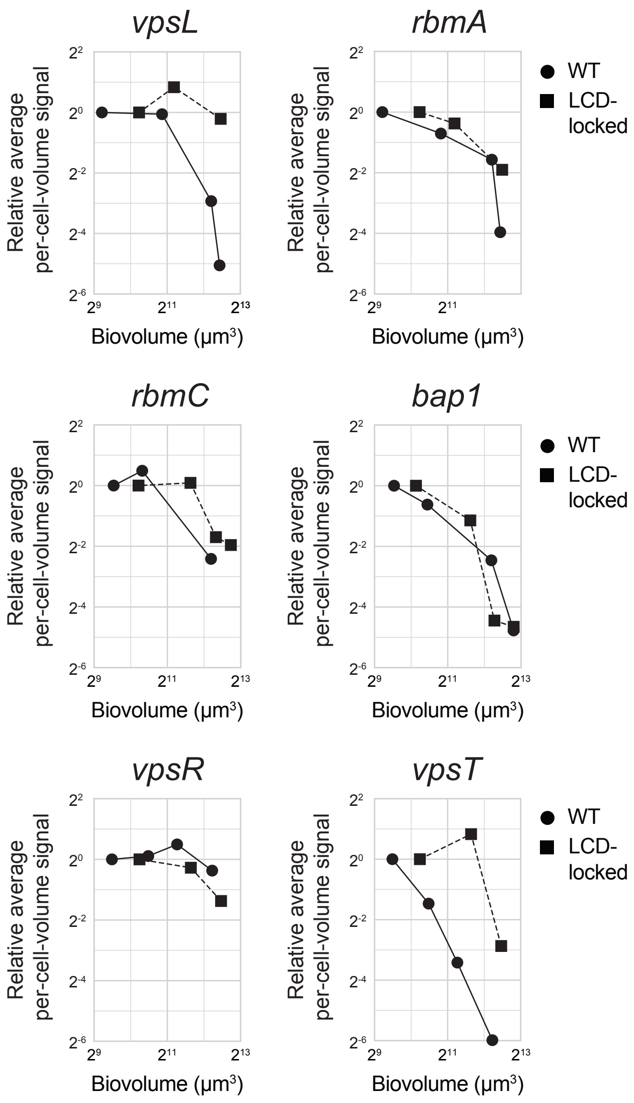

Fig. 6C, non-corrected data

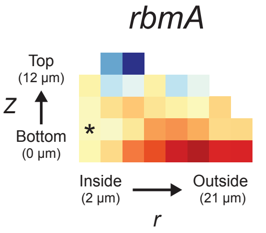

Fig. 6E, non-corrected data

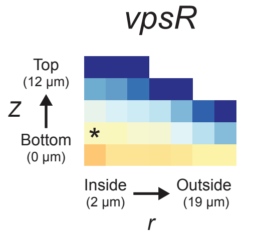

Fig. 6D, non-corrected data

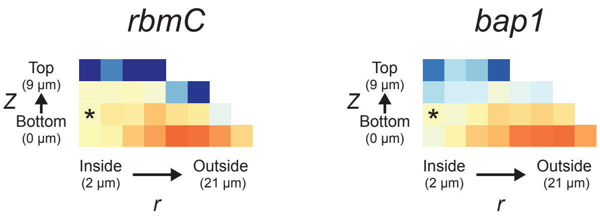

Fig. 6F, non-corrected data

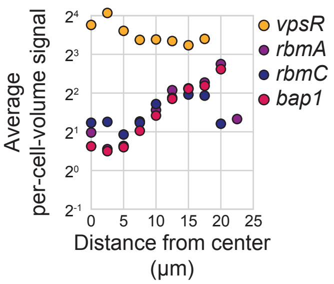

Fig. S6A, non-corrected data

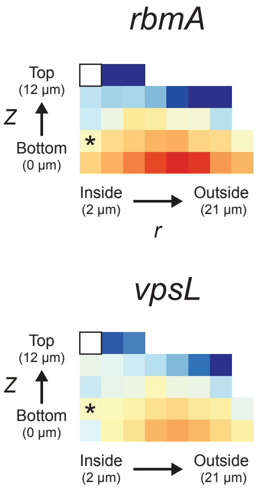

Fig. S6B, non-corrected data

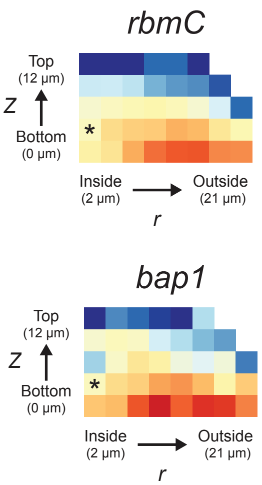

Fig. S6C, non-corrected data

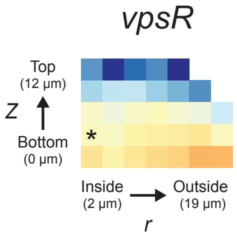

Fig. S6D, non-corrected data

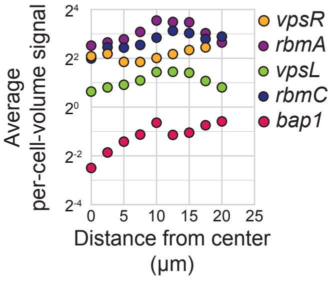

Fig. 7A, non-corrected data

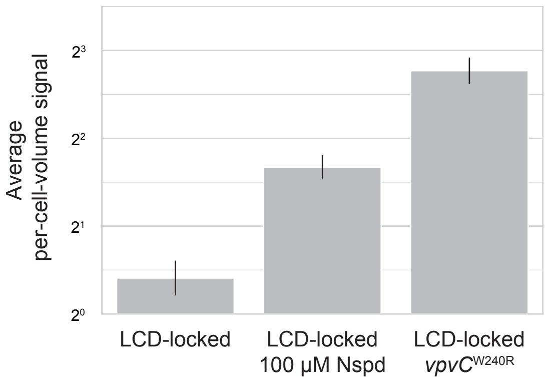

Fig. 7B, non-corrected data

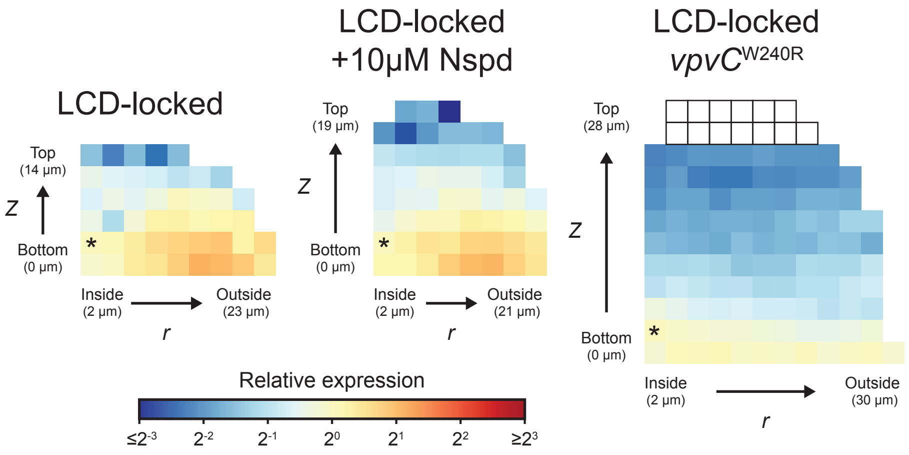

Fig. 7C, non-corrected data

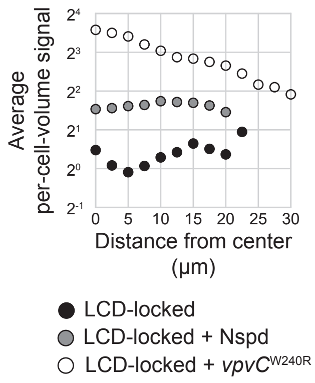

Supplement: Supplementary file 2 [file media-3.pdf]
